# The new (dis)order in RNA regulation

**DOI:** 10.1186/s12964-016-0132-3

**Published:** 2016-04-06

**Authors:** Aino I. Järvelin, Marko Noerenberg, Ilan Davis, Alfredo Castello

**Affiliations:** Department of Biochemistry, University of Oxford, South Parks Road, Oxford, OX1 3QU UK

**Keywords:** RNA-binding protein, Intrinsically disordered protein, Co- and post-transcriptional RNA regulation, RS repeat, RGG-box, GAR repeat, Basic patch, Poly-K patch, Arginine-rich motif, RNA granule

## Abstract

RNA-binding proteins play a key role in the regulation of all aspects of RNA metabolism, from the synthesis of RNA to its decay. Protein-RNA interactions have been thought to be mostly mediated by canonical RNA-binding domains that form stable secondary and tertiary structures. However, a number of pioneering studies over the past decades, together with recent proteome-wide data, have challenged this view, revealing surprising roles for intrinsically disordered protein regions in RNA binding. Here, we discuss how disordered protein regions can mediate protein-RNA interactions, conceptually grouping these regions into RS-rich, RG-rich, and other basic sequences, that can mediate both specific and non-specific interactions with RNA. Disordered regions can also influence RNA metabolism through protein aggregation and hydrogel formation. Importantly, protein-RNA interactions mediated by disordered regions can influence nearly all aspects of co- and post-transcriptional RNA processes and, consequently, their disruption can cause disease. Despite growing interest in disordered protein regions and their roles in RNA biology, their mechanisms of binding, regulation, and physiological consequences remain poorly understood. In the coming years, the study of these unorthodox interactions will yield important insights into RNA regulation in cellular homeostasis and disease.

**Electronic supplementary material:**

The online version of this article (doi:10.1186/s12964-016-0132-3) contains supplementary material, which is available to authorized users.

## Plain English summary

DNA is well known as the molecule that stores genetic information. RNA, a close chemical cousin of DNA, acts as a molecular messenger to execute a set of genetic instructions (genes) encoded in the DNA, which come to life when genes are activated. First, the genetic information stored in DNA has to be copied, or transcribed, into RNA in the cell nucleus and then the information contained in RNA must be interpreted in the cytoplasm to build proteins through a process known as translation. Rather than being a simple process, the path from transcription to translation entails many steps of regulation that make crucial contributions to accurate gene control. This regulation is in large part orchestrated by proteins that bind to RNA and alter its localisation, structure, stability, and translational efficiency. The current paradigm of RNA-binding protein function is that they contain regions, or domains, that fold tightly into an ordered interaction platform that specifies how and where the interaction with RNA will occur. In this review, we describe how this paradigm has been challenged by studies showing that other, hitherto neglected regions in RNA-binding proteins, which in spite of being intrinsically disordered, can play key functional roles in protein-RNA interactions. Proteins harbouring such disordered regions are involved in virtually every step of RNA regulation and, in some instances, have been implicated in disease. Based on exciting recent discoveries that indicate their unexpectedly pervasive role in RNA binding, we propose that the systematic study of disordered regions within RNA-binding proteins will shed light on poorly understood aspects of RNA biology and their implications in health and disease.

## Background

### Structural requirements for RNA-protein interactions

RNA-binding proteins (RBPs) assemble with RNA into dynamic ribonucleoprotein (RNP) complexes that mediate all aspects of RNA metabolism [1, 2]. Due to the prominent role that RBPs play in RNA biology, it is not surprising that mutations in these proteins cause major diseases, in particular neurological disorders, muscular atrophies and cancer [3–7]. Until recently, our understanding of how RBPs interact with RNA was based on a limited number of globular RNA-binding domains (RBDs), which include RNA-recognition motif (RRM), K-homology domain (KH), double-stranded RBD (dsRBD), zinc fingers (Znf), DEAD box helicase domain, and others (for recent reviews, see [8–10]). Each of these RBDs interacts with RNA following distinct mechanisms and differ in specificity and affinity for their target RNA. Promiscuous RNA binding is often mediated by interactions with the phosphate-sugar backbone, whereas sequence-specificity builds on interactions with the nucleotide base and shape complementarity between protein and RNA interfaces. While the most common RBDs interact with short (4–8 nt) sequences, others display lower or complete lack of sequence selectivity, recognising either the RNA molecule itself or secondary and three-dimensional structures [8, 11]. As the affinity and specificity of a single RBD is often insufficient to provide selective binding in vivo, RBPs typically have a modular architecture containing multiple RNA-interacting regions [8]. RNA-binding proteins are typically conserved, abundant, and ubiquitously expressed, reflecting the core importance of RNA metabolism in cellular physiology [12, 13].

## The coming of age for RNA-binding proteins — the emerging role of protein disorder

Early on, it was recognised that not all RNA-binding activities could be attributed to classical RBDs. Computational predictions based on transcriptome complexity suggested that 3-11 % of a given proteome should be dedicated to RNA binding, whereas only a fraction of this number could be identified by homology-based searches for classical RBDs [14, 15]. Moreover, there were several reports of RNA-binding activities within protein domains lacking similarities to any classical RBD [16, 17]. A number of studies showed that intrinsically disordered regions, lacking any stable tertiary structure in their native state, could contribute to RNA binding. For example, the flexible linker regions that separate the two RRMs of the poly(A)-binding protein (PABP) and polypyrimidine tract binding protein 1 (PTBP1), not only orientate the domains with respect each other, but also mediate RNA binding [18]. Flexible regions in RBPs rich in serine and arginine (S/R) and arginine and glycine (R/G) were found to contribute, or even to account for, RNA-binding activities [19, 20]. Furthermore, early computational analyses revealed that proteins involved in transcription and RNAs processing are enriched in disordered protein regions [21, 22], hinting on a broader role for protein disorder in RNA metabolism.

**Fig. 1:**
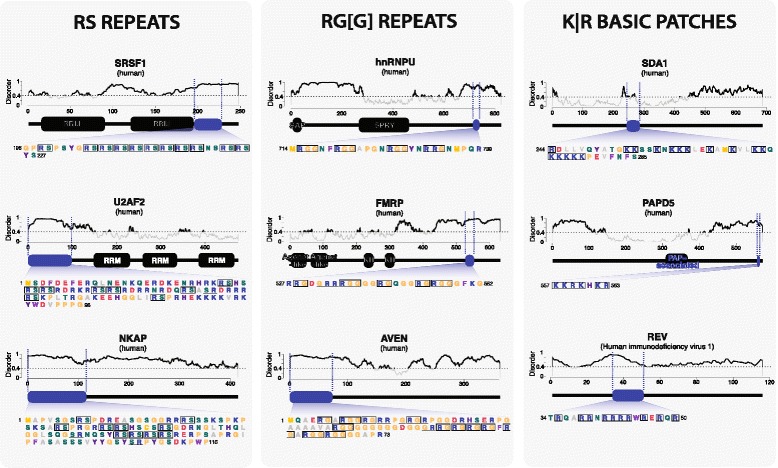
Three classes of disordered protein regions involved in direct RNA-interactions. Blue oval indicates the disordered region of each protein involved in RNA binding. Sequence is shown below the protein model, and typical sequence characteristics are indicated by boxes. Disorder profile was calculated using IUPred [172]. Values above 0.4 are considered disordered

Recently, the development of proteome-wide approaches for comprehensive determination of the RBP repertoire within the cell (RBPome) has substantially increased the number of known unorthodox RBPs. *In vitro* studies in yeast identified dozens of proteins lacking classical RBDs as putative RBPs, including metabolic enzymes and DNA-binding proteins [23, 24]. Two recent studies that employed in vivo UV crosslinking, poly(A)-RNA capture, and mass spectrometry, identified more than a thousand proteins interacting with RNA, discovering hundreds of novel RBPs [25, 26]. Strikingly, both known and novel RBPs were significantly enriched in disordered regions compared with the total human proteome. Approximately 20 % of the identified mammalian RBPs (~170 proteins) were disordered by over 80 % [25, 27]. Apart from the disorder-promoting amino acids such as serine (S), glycine (G), and proline (P), these disordered regions were enriched in positively (K,R) and negatively (D, E) charged residues as well as tyrosine (Y) [25], amino acids often found at RNA-interacting surfaces in classical RBDs [8]. Disordered amino acid sequences in RBPs form recognisable patterns that include previously reported motifs such as RG-and RS-repeats as well as new kinds of motifs, such as K or R-rich basic patches (Fig. 1). As with classical RBDs, disordered regions also occur in a modular manner in RBPs, repeating multiple times in a non-random manner across a given protein and, in some instances, combining with globular domains [25]. Taken together, these observations suggest that disordered regions 1) contribute to RBP function; 2) combine in a modular manner with classical RBDs suggesting functional cooperation; and 3) may play diverse biological roles, including RNA binding. Supporting this, a recent report has shown that globular RBDs are on average well conserved in number and sequence across evolution, while disordered regions of RBPs have expanded correlating with the increased complexity of transcriptomes [13]. What is the contribution and functional significance of protein disorder in RNA-protein interactions? Below, we will discuss what is known about disordered regions in RNA binding and metabolism, as well as physiology and disease, based on accumulating literature (Table 1, Additional file 1: Figure S1).

**Table 1:** Examples of RNA binding proteins where a disordered, non-classical region is involved in direct RNA binding. Additional details for each protein are presented in Additional file 1: Figure S1. Disorder prediction was calculated using IUPred [172]

Protein						Properties of disorder involved in RNA binding						
ID	Name	Aliases	Species	Canonical domains	Function	Class	Sequence	Disorder assignment	Target RNA preference	Regulation at disordered region	Interaction with other biomolecules	Ref
SRSF1	Serine/arginine-rich splicing factor 1	ASF, SF2, SF2P33, SFRS1	Homo sapiens	2xRRM	RNA splicing. Essential for heart development.	RS	^196−^GPRSPSYGRSRSRSR SRSRSRSRSNSRSRS YS^−227^	Experimental	-	Serine phosphorylated. Becomes more structured upon phosphorylation. Alternatively spliced.	Protein	[36, 39, 173–175]
U2AF2	Splicing factor U2AF 65 kDa subunit	U2AF65	Homo sapiens	3xRRM	RNA splicing.	RS	^1−^MSDFDEFERQLNENK QERDKENRHRKRSHS RSRSRDRKRRSRSRD RRNRDQRSASRDRRR RSKPLTRGAKEEHGG LIRSPRHEKKKKVRK YWDVPPPG^−98^	Predicted	No specificity	Serine phosphorylation, lysine acetylation, lysine hydroxylation ^a^	Protein	[19, 176]
NKAP	NF-kappa-B-activating protein	-	Homo sapiens	None	RNA splicing, transcriptional repression.	RS	^1−^MAPVSGSRSPDREAS GSGGRRRSSSKSPKP SKSARSPRGRRSRSH SCSRSGDRNGLTHQL GGLSQGSRNQSYRSR SRSRSRERPSAPRGI PFASASSSVYYGSYS RPYGSDKPWP^−115^	Predicted	poly (U)	Lysine acetylation ^a^	Protein	[43]
Nucleo-capsid protein	-	Nucleoprotein, NC, N	Severe acute respiratory syndrome coronavirus (SARS-CoV)	None	Major structural component of virions that associates with genomic RNA to form a long, flexible, helical nucleo-capsid.	Other, RS, polyK/ other	^1−^MSDNGPQSNQRSAPR ITFGGPTDSTDNNQN GGRNGARPKQRRPQ^−44^, ^182−^QASSRSSSRSRGNSR NSTPGSSRGNSPARM ASGGGETALALLLLDR LNQLESKVSGKGQQQ QGQTV^−247^, ^366−^PTEPKKDKKKKTDEA QPLPQRQKKQPTVTL LPAADMDDFSRQLQN SMSGASADSTQ^−422^	Experimental	poly (U) ssRNA	-	-	[46, 177]
ALYREF	Aly/REF export factor 2	Alyref	Mus musculus	1xRRM	RNA export.	RG	^22−^VNRGGGPRRNRPAIA RGGRNRPAPYSR^−48^	Experimental	-	TAP displaces RNA from ALYREF	Protein	[54, 55, 57, 178]
Aven	Cell death regulator Aven	-	Homo sapiens	None	Positive translational regulator.	RG	^1−^MQAERGARGGRGRRP GRGRPGGDRHSERPG AAAAVARGGGGGGGG DGGGRRGRGRGRGFR GARGGRGGGGAPR^−73^	Predicted	RNA G-quadruplex	Methylated (no influence on RNA binding; influences protein interactions and polysome association). Alternative transcript (mouse)	Protein	[179, 180]
Caprin-1	-	GPIAP1, GPIP137, M11S1, RNG105	Homo sapiens, Xenopus	None	Regulation of localised translation, synaptic plasticity, cell proliferation and migration.	RG	^612−^RGGSRGARGLMNGYR GPANGFRGGYDGYRP SFSNTPNSGYTQSQF SAPRDYSGYQRDGYQ QNFKRGSGQSGPRGA PRGRGGPPRPNRGMP QMNTQQV^−708^ (human), ^578−^RGMARGGQRGNRGMM NGYRGQSNGFRGG^−605^ (Xenopus)	Predicted	-	The end of the human sequence (RGGPPRPNRGMPQMNTQQV) is in an alternative isoform ^a^	-	[181, 182]
DDX4	Probable ATP-dependent RNA helicase DDX4	Vasa	Homo sapiens	None	RNA helicase.	RG	^1−^MGDEDWEAEINPHMS SYVPIFEKDRYSGEN GDNFNRTPASSSEMD DGPSRRDHFMKSGFA SGRNFGNRDAGECNK RDNTSTMGGFGVGKS FGNRGFSNSRFEDGD SSGFWRESSNDCEDN PTRNRGFSKRGGYRD GNNSEASGPYRRGGR GSFRGCRGGFGLGSP NNDLDPDECMQRTGG LFGSRRPVLSGTGNG DTSQSRSGSGSERGG YKGLNEEVITGSGKN SWKSEAEGGES^−236^	Experimental	Single-stranded DNA.	Arginine methylation. Alternative isoforms ^a^	-	[130]
EWS	RNA-binding protein EWS	EWSR1	Homo sapiens	1xRRM	Transcription, splicing.	RG	^288−^PGENRSMSGPDNRGR GRGGFDRGGMSRGGR GGGRGGMGSAGERGG FNKPGGPMDEGPDLD LGPPVDP^−354^, ^450−^PMNSMRGGLPPREGR GMPPPLRGGPGGPGG PGGPMGRMGGRGGDR GGFPPRG^−501^, ^545−^APKPEGFLPPPFPPP GGDRGRGGPGGMRGG RGGLMDRGGPGGMFR GGRGGDRGGFRGGRG MDRGGFGGGRRGGPG GPPGPLMEQMGGRRG GRGGPGKMDKGEHRQERRDRPY^−656^	Predicted	G-quadruplex (RGG3, not RGG1 or RGG2)	Alternative splicing ^a^. Arginine dimethylation at RGG repeats affects protein sub cellular localization	DNA (via RGG3). All three RGG repeats bind SMN protein.	[183–187]
FMRP	Fragile X mental retardation protein 1	FMR1	Homo sapiens, mouse	2xKH	Regulation of translation (repressor).	RG	^527−^RRGDGRRRGGGGRGQ GGRGRGGGFKG^−552^	Experimental	G quartets, G-quadruplex	Arg methylation. Alternative splicing at regions flanking the RGG-box alters FMRP’s capacity to bind RNA, to be methylated, and associate with polysomes.	C-terminal part of this protein that also includes the RG region is involved in protein-protein interactions.	[68–70, 72, 75–78, 152, 188, 189]
FUS	RNA-binding protein FUS	TLS	Homo sapiens, Drosophila melanogaster	1xRRM	Splicing, poly-adenylation.	RG	^213−^RGGRGRGG^−220^, ^241−^PRGRGGGRGGRGG^−253^, ^377−^RGGGNGRGGRGRGGP MGRGGYGGGGSGGGG RGG^−409^, ^472−^RRGGRGGYDRGGYRG RGGDRGGFRGGRGGG DRGG^−505^	Predicted	G-quadruplex	Arginine methylation.	-	[190–193]
hnRNP U	Heterogeneous nuclear ribonucleoprotein U	HNRPU, SAFA, U21.1	Homo sapiens	None	RNA stability, U2 snRNP maturation, DNA binding.	RG	^714−^MRGGNFRGGAPGNRG GYNRRGNMPQR^−739^	Predicted	Poly (U and poly (G) homopolymers, UGUGG	-	DNA	[20, 51]
ICP27	Infected cell protein 27, Immediate-early protein IE63	-	Herpes simplex virus	None	RNA export.	RG	^138−^RGGRRGRRRGRGRGG^−152^	Predicted	poly (G) and poly (U) homopolymers, GC-rich sequences	Methylated	-	[194–196]
LAF1	-	DDX3	C. elegans	None	RNA helicase.	RG	^1−^MESNQSNNGGSGNAA LNRGGRYVPPHLRGG DGGAAAAASAGGDDR RGGAGGGGYRRGGGN SGGGGGGGYDRGYND NRDDRDNRGGSGGYG RDRNYEDRGYNGGGG GGGNRGYNNNRGGGG GGYNRQDRGDGGSSN FSRGGYNNRDEGSDN RGSGRSYNNDRRDNG GDG^−168^	Experimental	-	Region 43–106 containing RG-repeat is alternative.	-	[142]
NXF1	Nuclear RNA export factor 1	TAP	Mus musculus, homo sapiens	None	Nuclear export.	RG	^2−^ADEGKSYSEHDDERV NFPQRKKKGRGPFRW KYGEGNRRSGRGGSG IRSSRLEEDDGDVAM SDAQDGPRVRYNPYT TRPNRRGDTWHDRDR IHVTVRRDRAPPERG GAGTSQDGTSKN^−118^	Predicted	Non-specific	-	Protein. Overlaps a nuclear localisation and export signals.	[55, 197, 198]
Nucle- olin	-	NCL, Protein C23	Hamster	4xRRM	Chromatin decondensation, pre-rRNA transcription, ribosome assembly.	RG	^630−^MEDGEIDGNKVTLDW AKPKGEGGFGGRGGG RGGFGGRGGGRGGGR GGFGGRGRGGFGGRG GFRGGRGGGGGGGDF KPQGKKTKFE^−714^	Experimental. Suggested to form a flexible β-spiral.	None	-	Protein (in human)	[199, 200]
RBMX	RNA-binding motif protein, X chromosome	HNRPG, RBMXP1	Homo sapiens, Xenopus laevis	1xRRM	Regulation of transcription, splicing.	RG	^333−^DLYSSGRDRVGRQER GLPPSMERGYPPPRD SYSSSSRGAPRGGGR GGSRSDRGGGRSR^−390^	Predicted	C-terminal regions binds structured (hairpin) RNA	Identical C-terminal sequence is mouse RBMX is alternatively spliced.	-	[201–206]
Foamy virus Gag	-	-	Human foamy virus	None	Viral genome binding, capsid formation.	RG	^485−^RPSRGRGRGQN^−495^	Predicted	-	-	-	[207–210]
TERF2	Telomeric repeat-binding factor 2	TRBF2, TRF2	Homo sapiens	None	Presynaptic plasticity, axonal mRNA transport, telomere maintenance	RG	^43−^MAGGGGSSDGSGRAAGRRASRSSGRARRGRHEPGLGGPAERGAG^- 86^	Predicted	G-rich, TERRA	Arginine methylation	Protein	[211–214]
XTUT7	-	-	Xenopus laevis	Zinc finger	RNA polyuridylat-ion, translational repression.	Basic patch (poly R)	^453−^MRRNRVRRRNNENAG NQRY^−471^	Predicted	-	-	-	[215]
Tat	Transactivating regulatory protein	-	Human immuno-deficiency virus (HIV)	None	transcriptional activator, transcription elongation.	Basic patch (poly R)	^49−^RKKRRQRRR^−57^	Experimental	Structured RNA (HIV-1 Trans-activation response element, TAR)	Arginine methylation (with impact on RNA binding). Lysine acetylation (impact on TAR binding, through an effect on Tat-TAR-CyclinT1 ternary complex formation).	Protein	[85, 88–91, 93, 216–223]
Rev	Regulator of expression of viral proteins	-	Human immuno-deficiency virus (HIV)	None	RNA export.	Basic patch (poly R)	^34−^TRQARRNRRRRWRER QR^−50^	Experimental	Structured RNA (HIV-1 Rev response element, RRE)	Arginine methylation.	Protein	[96–101, 103, 104, 153, 154, 224]
Tat	Transactivating regulatory protein	S ORF, bTat	Bovine immunodeficiency virus	None	Transcriptional activator	Basic patch (polyR)	^70−^RGTRGKGRRIRR^−81^	Experimental	Structured RNA (TAR)	-	Protein	[91]
Coat protein	-	-	Alfalfa mosaic virus	None	Capsid protein, viral RNA. Translation initiation.	Basic patch (poly K)	^6−^KKAGGKAGKPTKRSQ NYAALRK^−27^	Experimental	-	-	-	[225, 226]
PAPD5	Non-canonical poly (A) RNA polymerase PAPD5	-	Homo sapiens	None	RNA oligoadenylation, RNA stability	Basic patch (poly K)	^557−^KKRKHKR^−563^	Predicted	May have a preference for structured RNA	Alternative splicing ^a^	-	[109]
SDAD1	Protein SDA1 homolog	-	Homo sapiens	None	Protein transport, ribosomal large subunit export from nucleus.	Basic patch (poly K)	^244−^RDLLVQYATGKKSSK NKKKLEKAMKVLKKQ KKKKKPEVFNFS^−285^	Predicted	-	-	-	[58]
HMGA1	High mobility group protein HMG-I/HMG-Y	-	Homo sapiens	None	-	(e) AT	^21−^TEKRGRGRPRK^−31^	Experimental	Binds structured RNA.	Arginine methylation.	DNA	[121, 124, 125, 127]
Tip5	Bromodomain adjacent to zinc finger domain protein 2A	BAZ2A	Homo sapiens	None	Epigenetic rRNA gene silencing.	(e) AT	^650−^GKRGRPRNTEK^−660^, ^670−^KRGRGRPPKVKIT^−682^	Experimental	Exhibits preferential binding towards dsRNA	-	DNA	[127, 227, 228]
PTOV1	Prostate tumor-overexpressed gene 1 protein	ACID2, PP642	Homo sapiens	None	Regulation of transcription.	(e) AT	^1−^MVRPRRAPYRSGAGG PLGGRGRPPRPLVVR AVRSRSWPASPRG^−43^	Predicted	Exhibits preferential binding towards dsRNA	Alternative splicing ^a^	DNA	[127]
GPBP1	-	Vasculin, GPBP, SSH6	Homo sapiens	None	Transcription factor, positive regulation of transcription	e (AT)	^38−^NRYDVNRRRHNSSDG FDSAIGRPNGGNFGR KEKNGWRTHGRNG^−80^	Predicted	Exhibits preferential binding towards dsRNA	Alternative splicing ^a^	DNA	[127]
SRSF2	Serine/arginine-rich splicing factor 2	SFRS2	Homo sapiens	1xRRM	RNA splicing.	Other (GRP)	^1−^MSYGRPPP^−8^, ^93−^GRPPDSHHS^−101^	Experimental	UCCA/UG, UGGA/UG	-		[229, 230]
Tra2-β1	Transformer-2 protein homolog beta	TRA2B, SFRS10	Homo sapiens	1xRRM	RNA splicing.	Other	^110−^NRANPDPNCC^−119^, ^194−^SITKRPHT^−201^	Experimental	GAAGAA (primary), AGAAG (primary), GACUUCAACA AGUC (structured)	-	-	[40, 231–233]
hnRNPA1	Heterogeneous nuclear ribonucleoprotein A1	HNRPA1	Human, Xenopus tropical	2xRRM	hnRNP particle formation, nucleo-cytoplasmic transport, splicing.	Other/RG	^186−^MASASSSQRGRSGSG NFGGGRGGGFGGNDN FGRGGNFSGRGGFGG SRGGGGYGGSGDGYN GFGNDGGYGGGGPGY SGGSRGYGSGGQGYG NQGSGYGGSGSYDSY NNGGGGGFGGGSGSN FGGGGSYNDFGNYNN QSSNFGPMKGGNFGG RSSGPYGGGGQYFAK PRNQGGYGGSSSSSS YGSGRRF^−372^	Predicted	-	Region containing the RG- and FG-repeat peptides is alternatively spliced. RG-region may mediate RNA binding. The entire region is involved in hnRNPA1 aggregation and includes a nuclear targeting sequence.	-	[136, 234–237]
LUZP4	Leucine zipper protein 4	CT-28,	Homo sapiens	None	Nuclear export.		^51−^RQNHSKKESPSRQQSKAHRHRHRRGYSRCR^−80^, ^238−^LVDTQSDLIATQRDLIATQKDLIATQRDLIATQRDLIVTQRDLVATERDL^−287^	Predicted	-	Alternative splicing affecting the first, R-rich region ^a^	Protein	[197]
ORF57	52 kDa immediate-early phosphoprotein, mRNA export factor ICP27 homolog	-	Herpes-virus saimiri	None	Viral RNA regulation.	Other	^64−^RQRSPITWEHQSPLS RVYRSPSPMRFGKRP RISSNSTSRSCKTSW ADRVREAAAQRR^−120^	Experimental	Viral RNA: GAAGAGG, CAGUCGCGAAGAGG	RNA binding region partially overlaps with ALYREF binding site.	Protein	[178]
APC	Adenomatous polyposis coli protein	-	Mus musculus	None	Microtubule binding, negative regulator of Wnt signaling.	Other	^2223−^SISRGRTMIHIPGLR NSSSSTSPVSKKGPP LKTPASKSPSEGPGA TTSPRGTKPAGKSEL SPITRQTSQISGSNK GSSRSGSRDSTPSRP TQQPLSRPMQSPGRN SISPGRNGISPPNKL SQLPRTSSPSTASTK SSGSGKMSYTSPGRQ LSQQNLTKQASLSKN ASSIPRSESASKGLN QMSNGNGSNKKVELS RMSSTKSSGSESDSS ERPALVRQSTFIKEA PSPTLRRKLEESASF ESLSPSSRPDSPTRS QAQTPVLSPSLPDMS LSTHPSVQAGGWRKL PPNLSPTIEYNDGRP TKRHDIARSHSESPS RLPINRAGTWKREHS KHSSSLPRVSTWRRT GSSSSILSASSE^−2579^	Predicted	G-rich motif	-	-	[238]
CTCF	Transcriptional repressor CTCF	-	Homo sapiens	11x Zn finger (3 according to Pfam)	-	Other	^575−^DNCAGPDGVEGENGG ETKKSKRGRKRKMRS KKEDSSDSENAEPDL DDNEDEEEPAVEIEP EPEPQPVTPAPPPAK KRRGRPPGRTNQPKQ NQPTAIIQVEDQNTG AIENIIVEVKKEPDA EPAEGEEEEAQPAAT DAPNGDLTPEMILSM MDR^−727^	Predicted	-	Serine phosphorylation ^a^	-	[239]
Df31	Decondensation factor 31	Anon1A4	D. melanogaster	None	Regulation of higher-order chromatin structure, maintenance of open chromatin.	Other	^1−^MADVAEQKNETPVVE KVAAEEVDAVKKDAV AAEEVAAEKASITEN GGAEEESVAKENGAA DSSATEPTDAVDGEK ASEPTVSFAADKDEK KDEDKKEDSAADGED TKKESSEAVLPAVEN GSEEVTNGDSTDAPA IEAVKRKVDEAAAKA DEAVATPEKKAKLDE ASTKDEVQNGAEASE VAA^−183^	Experimental	Non-specific but does not bind ssDNA or dsDNA. Preferentially binds snoRNA.	-	-	[127, 240]
Ezh2	Histone-lysine N-methyltransferase EZH2	Enx1h	Mus musculus	None	Polycomb group protein. Involved in H3 methylation (H3K9me and H3K27me).	Other	^342−^RIKTPPKRPGGRRRG RLPNNSSRPSTPTI^−370^	Predicted	May have a preference for RNA stem loops.	1st Thr is phosphorylated in a cell cycle dependent manner. Phosphorylation increases RNA binding.	This region overlaps a region involved in protein-protein interactions in human, however, RNA and protein binding regions may be distinct from one another.	[241–243]
Nrep	Neuronal regeneration-related protein	P311	Mus musculus	None	Axonal regeneration, cell differentiation.	Other	^27−^KGRLPVPKEVNRKKM EETGAASLTPPGSRE FTSP^−60^	Experimental	-	-	Protein	[244]
Gemin5	Gem-associated protein 5	-	Homo sapiens	None	snRNP assembly, splicing, IRES-mediated translation initiation.	Other	^1297−^PNSSVWVRAGHRTLS VEPSQQLDTASTEET DPETSQPEPNRPSEL DLRLTEEGERMLSTF KELFSEKHASLQNSQ RTVAEVQETLAEMIR QHQKSQLCKSTANGP DKNEPEVEAEQ^−1412^, ^1383−^EMIRQHQKSQLCKSTANGPDKNEPEVEAEQPLCSSQSQCKEEKNEPLSLPELTKRLTEANQRMAKFPESIKAWPFPDVLECCLVLLLIRSHFPGCLAQEMQQQAQELLQKYGNTKTYRRHCQTFCM^−1508^	Experimental	-	-	-	[245]
Nup153	-	-	Homo sapiens	None	Component of the nucleopore, RNA trafficking.	Other	^250−^KTSQLGDSPFYPGKT TYGGAAAAVRQSKLR NTPYQAPVRRQMKAK QLSAQSYGVTSSTAR RILQSLEKMSSPLAD AKRIPSIVSSPLNSP LDRSGIDITDFQAKR EKVDSQYPPVQRLMT PKPVSIATNRSVYFK PSLTPSGEFRKTNQR I^−400^	Predicted	Single-stranded RNA with little sequence preference	Serine and threonine phosphorylation ^a^	-	[246, 247]
SCML2	Sex comb on midleg-like protein 2	-	Homo sapiens	None	Binds Polycomb Repressive Complex 1 and histones. Involved in epigenetic silencing.	Other	^256−^SPSEASQHSMQSPQK TTLILPTQQVRRSSR IKPPGPTAVPKRSSS VKNITPRKKGPNSGK KEKPLPVICSTSAAS^−330^	Predicted	No specificity, but discriminates between RNA and DNA.	Alternative isoform ^a^ , Serine phosphorylation ^a^	-	[248]
KDM4D	Lysine-specific demethylase 4D	JMJD2D	Homo sapiens	None	Demethylates lysine 9 on histone H3.	Other	^348−^MEPRVPASQELSTQK EVQLPRRAALGLRQL PSHWARHSPWPMAAR SGTRCHTLVCSSLPR RSAVSGTATQPRAAA VHSSKKPSSTPSSTP GPSAQIIHPSNGRRG RGRPPQKLRAQELTL QTPAKRPLLAGTTCT ASGPEPEPLPEDGAL MDKPVPLSPGLQHPV KASGCSWAPVP^−523^	Experimental	-	-	-	[249]
-	-	-	Synthetic	None	Bind HIV RNA (RRE)	Other/polyR	SRSSRRNRRRRRRR, NHRRRRRQRRRRRR, SPCRSRRSGSSRRRRRRR	Experimental	Structured RNA (HIV-1 Rev response element, RRE)	-	-	[105]

^a^ According to uniprot, from a large-scale study but no detailed experimental confirmation available

## Review

### Disordered RS repeats put RNA splicing in order

Disordered, arginine and serine (RS) repeat containing regions occur in a number of human proteins referred to as SR proteins and SR-like proteins (reviewed in [28, 29]). SR proteins are best known for their roles in enhancing splicing but have been ascribed functions in other RNA processes from export, translation, and stability to maintenance of genome stability (e.g. [30, 31] for reviews). There are twelve SR proteins in human that contain 1–2 classical RRMs and an RS repetitive motif of varying length [30]. Classical SR proteins bind exonic splicing enhancers in nascent RNA through their RRMs and promote splicing of adjacent introns [32, 33]. The RS repeat enhances splicing in a length-dependent manner [34]. RS repeats are predicted to be intrinsically disordered [35] (Table 1), but phosphorylation promotes a transition towards a less flexible, arch-like structure with an influence on RNA binding in the serine/arginine-rich splicing factor 1 (SRSF1) [36] (Fig. 1). RS repeats have been shown to directly bind RNA during multiple steps of splicing [19, 37–39] and to contribute to binding affinity of RRMs for RNA by inducing a higher affinity form of the RRM [40]. RS repeats can also mediate protein-protein interactions [28, 33], hence their association with RNA can also be indirect. RS-mediated protein binding seems to be compatible with RNA binding [33, 41], suggesting that protein and RNA binding could take place simultaneously or sequentially. RNA-binding by RS repeats seems to be rather non-specific, as motif shortening, replacement of arginine for lysine, amino acid insertion, and replacement for a homologous sequences are well tolerated [19, 37, 38]. In summary, there is compelling evidence that disordered RS protein motifs play an important role in RNA splicing, and that the interaction between these repeats and RNA occurs mostly in a sequence-independent manner. Nevertheless, it remains to be determined how many of the SR proteins interact with RNA through the RS repeats, and whether the differences in RS repeat length have a direct effect on RNA binding affinity or specificity.

Certain members of the SR-related protein family lack RRMs and are involved in diverse RNA metabolic processes [42]. For example, NF-kappa-B-activating protein (NKAP) (Fig. 1) is an SR-related protein, with a newly discovered role in RNA splicing [43], but originally known for its roles in NF-kappa-B activation [44] and as a transcriptional repressor of Notch-signalling in T-cell development [45]. This protein binds RNA through its RS repeat, in cooperation with an RBD at its C-terminal region. A transcriptome-wide study showed this protein targets diverse classes of RNAs, including pre-mRNAs, ribosomal RNAs and small nuclear RNAs [43]. RNA-binding RS repeat sequences can also be found in viral proteins, such as the nucleocapsid of severe acute respiratory syndrome coronavirus (SARS-CoV), causative agent of the alike-named disease. This protein employs RS-rich disordered region, in cooperation with other RNA-binding regions, to capture viral RNA and package it into virions [46]. Taken together, these reports suggest that RS repeats have broader roles in RNA-binding than previously anticipated.

## RG-rich repeats — The swiss-army knife of protein-RNA interactions

A commonly occurring disordered RNA–binding motif in RBPs consists of repeats of arginine and glycine, termed RGG-boxes or GAR repeats. These sequences are heterogeneous both in number of repeats and in their spacing. A recent analysis divided these RG-rich regions into di- and tri-RG and -RGG boxes, and identified instances of such repeats in order of tens (di- and tri-RGG) to hundreds (tri-RG) and nearly two thousand (di-RG) proteins [47]. Proteins containing such repeats are enriched in RNA metabolic functions [47]. However, it is not currently clear whether the different repeat architectures provide distinct functional signatures.

The RGG box was first identified in the heterogeneous nuclear ribonucleoprotein protein U (hnRNP-U, also known as SAF-A) as a region sufficient and required for RNA binding (Table 1, Fig. 1). hnRNP-U lacks canonical RBDs, but has semi-structured SAP domain involved in DNA binding [48–50]. hnRNP-U has been found to target hundreds of non-coding RNAs, including small nuclear (sn)RNAs involved in RNA splicing, and a number of long non-coding (lnc)RNAs, in an RGG-box-dependent manner [51]. RGG-mediated interaction of hnRNP-U with the lncRNAs Xist [52] and PANDA [53] has been implicated in epigenetic regulation.

RG[G] -mediated RNA binding also plays a role in nuclear RNA export, as illustrated by the nuclear RNA export factor 1 (NXF1). While NXF1 harbours an RRM capable of binding RNA [54], most of the in vivo RNA-binding capacity is attributed to the RGG-containing, N-terminal region [55] (Table 1). The arginines in this motif play a key role in the interaction with RNA, which has been shown to be sequence-independent but necessary for RNA export [55]. NXF1 overall affinity for RNA is low [55, 56], and requires the cooperation with the export adapter ALY/REF [57]. ALY/REF also bears an N-terminal disordered arginine-rich region that resembles an RGG-box [57] and mediates both RNA binding [54, 58, 59] and the interaction with NXF1 [60]. The activation of NXF1 is proposed to be triggered by the formation of a ternary complex between ALY/REF and NXF1, in which their RG-rich disordered regions play a central role. Analogous sequences has been identified in viral proteins and also facilitate viral RNA export by bypassing canonical nuclear export pathways (Table 1).

**Fig. 2:**
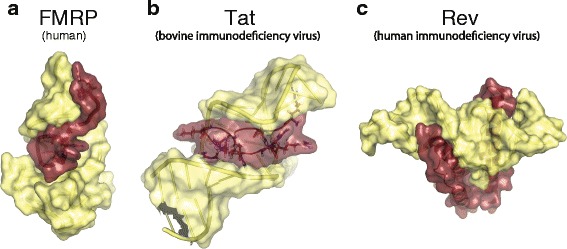
Structural examples RNA-bound disordered regions. **a** The RGG-peptide of the human FMRP bound to a *in vitro*-selected guanine-rich sc1 RNA determined by NMR (PDB 2LA5) [78] **b** Basic patch of disordered bovine immunodeficiency virus (BIV) Tat forms a β–turn when interacting with its target RNA, TAR. Structure determined by NMR (PDB 1MNB) [91] **c** Dimer of the basic patch containing Rev protein of the human immunodeficiency virus (HIV) in complex with target RNA, RRE, determined by crystallography [102] (PDB 4PMI). Red, peptide; yellow, RNA. Illustrations were created using PyMol

Fragile X mental retardation protein (FMRP) is another RBP with a well-characterised, RNA-binding RGG-box (Fig. 1). Involved in translation repression in the brain [61], loss of FMRP activity leads to changes in synaptic connectivity [62], mental retardation [63–65], and may also promote onset of neurodegenerative diseases [66]. In addition to its RGG-box, FMRP contains two KH domains that contribute to RNA binding. The RGG-box of FMRP has been shown to interact with high affinity with G-quadruplex RNA structures [67–77]. The RGG-box is unstructured in its unbound state [70, 78], but folds upon binding to a guanine-rich, structured G-quadruplex in target RNA [78] (Fig. 2). Both arginines and glycines play a key role in the function of the RGG-box and replacement of these amino acids impair RNA binding [78]. The arginine residues used to interact with RNA vary depending on the target RNA [70, 76, 78]. The FMRP RGG-box targets its own mRNA at an G-quadruplex structure that encodes the RGG-box [69]. This binding regulates alternative splicing of FMRP mRNA proximal to the G-quartet, suggesting it may auto-regulate the balance of FRMP isoforms [74]. Surprisingly, a recent transcriptome-wide study of polysome-associated FMRP found no enrichment for predicted G-quadruplex structures in the 842 high-confidence target mRNAs [79]. Another study identified FMRP binding sites enriched in specific sequence motifs, where the KH2 domains emerged as the major specificity determinants [80]. These results suggest that the role of RGG-box in this RBP could be limited to increase the overall binding affinity of the protein, supporting the sequence-specific interactions mediated by the KH2 domains. However, we cannot exclude the possibility of differential UV crosslinking efficiency of the KH2 domains and the RGG-box, which could result in biased binding signatures in CLIP studies.

A number of other RBPs use an RGG-repeat region to target G-rich and structured RNA targets and are implicated in neurological disease as well as cancer (Table 1). These RG-rich regions can mediate both unselective and specific interactions with RNA and can be involved in varied RNA metabolic processes.

## Catching the RNA with a basic arm

Basic residues often cluster in RBPs to form basic patches that can contribute to RNA-binding. Analysis of mammalian RNA-binding proteomes showed that such motifs are abundant among unorthodox RBPs [25, 27]. Basic patches are normally composed of 4–8 lysines (K) or, less frequently, arginines (R), forming a highly positive and exposed interface with potential to mediate molecular interactions [25]. Basic patches can occur at multiple positions within an RBP forming islands that often flank globular domains. This suggests functional cooperation between natively structured and unstructured regions [25]. Many RBPs contain alternating basic and acidic tracts that form highly repetitive patterns with unknown function [25]. Since acidic regions are not thought to interact with RNA [58], they may be involved in other intra- or intermolecular interactions, or contribute to accessibility and compaction of the region [81].

Arginine rich motifs (ARMs) (Table 1) are probably best characterised in viral proteins. These motifs tend to be disordered, and when bound to RNA, range from completely disordered to ordered but flexible. Although simple in terms of amino acid composition, ARMs seem to be able to target RNAs quite diversely and often specifically [82]. Lentiviral Tat proteins (Trans-Activator of Transcription) are key regulator of viral biological cycle by promoting viral gene expression upon binding to an RNA structure present at the 5’ end of the nascent viral RNA (called the trans-activation response element, TAR) [83]. Human immunodeficiency virus (HIV) Tat ARM is intrinsically disordered in its free-state [84–87]. Only one key arginine, flanked by basic amino acids, is required for specific interaction with TAR [88, 89]. Differences in the flanking basic amino acids contribute to selectivity between TARs from different viruses [90]. ARMs can accommodate different binding conformations depending on their target RNA. For example, bovine immunodeficiency virus (BIV) Tat ARM forms a beta-turn conformation upon binding to TAR [91] (Fig. 2c). Jembrana disease virus (JDV) Tat ARM can bind both HIV and BIV TARs, as well as its own TAR, but does so adopting different conformations and using different amino acids for recognition [92]. The RNA-binding disordered region of HIV Tat also mediates protein-protein interactions required for nuclear localisation [93]. Structural flexibility required to engage in diverse simultaneous or sequential RNA and protein interactions might explain why the native ARM-RNA interactions do not display very high affinity [92].

Similar to Tat proteins, lentiviral Rev auxiliary protein binds a structured RNA element (the Rev response element, RRE) present in partially-spliced and unspliced viral RNAs to facilitate nuclear export of viral RNA [94, 95]. The HIV Rev ARM was experimentally shown to be intrinsically disordered when unbound in physiological conditions [96–98] (Table 1, Fig. 1). Disorder-to-structure transition correlates with RNA binding and the RRE-bound Rev folds into an alpha-helical structure that maintains some structural flexibility [96–100]. Rev oligomerises and binds the multiple stems of the RRE using diverse arginine contacts, which results in a high-affinity ribonucleoprotein that allows efficient nuclear export of unspliced HIV RNAs [101–103]. Interestingly, Rev can also bind in an extended conformation to *in vitro* selected RNA aptamers [104], highlighting the role of RNA secondary and tertiary structure in the conformation that Rev adopts. The RRE can also be recognised by several different *in vitro* selected R-rich peptides that include additional serine, glycine, and glutamic acid residues [105–107] — these peptides are predicted to be disordered (Table 1). A simple, single nucleotide base changes in the RRE can direct affinity towards a particular ARM [108]. These features highlight the structural malleability of the Rev ARM, and suggest that some structural flexibility is relevant for in vivo binding.

The basic amino acid lysine can form disordered poly-lysine peptides that interact with RNA. 47 proteins identified in the human RNA-binding proteome have a long poly-K patch but lack known RBDs, suggesting these motifs are good candidates for RNA binding [25]. The K-rich C-terminal tail of protein SDA1 homolog (SDAD1) is composed of 45 amino acids, including 15 K, one R, two glutamines (Q) and two asparagines (N) (Table 1, Fig. 1). It binds RNA in vivo with similar efficiency as a canonical domain such as RRM [58]. The human non-canonical poly(A) polymerase PAPD5, that is involved in oligoadenylating aberrant rRNAs to target them for degradation [109, 110], also lacks canonical RBDs, but its C-terminal basic patch is directly involved in binding RNA (Fig. 1, Table 1). Removal or mutation of this sequence results in impaired RNA binding and reduced catalytic activity [109].

Basic tails in RBPs share physicochemical similarities with analogous sequences in DNA-binding proteins (DBPs) [111]. In DNA-binding context, basic patches are known to endow faster association with DNA due to increased ‘capture radius’ as well as to promote hopping and sliding movements along DNA molecules [112–118]. DNA binding through basic tails seems to be sequence-independent [119] and structural studies have shown that basic residues are projected into the minor grove of the double stranded DNA helix, establishing numerous electrostatic interactions with the phosphate-sugar backbone [116, 120]. Basic patches in RBPs may modulate RNA searching and binding avidity in a similar manner.

One open question is whether basic tails can distinguish between DNA and RNA. The AT-hook, defined as G-R-P core flanked by basic arginine and/or lysine residues, binds DNA and is found in many nuclear, DNA-binding proteins [121, 122]. However, this motif has been recently shown also to bind RNA [123–126]. Furthermore, an extended AT-hook (Table 1), occurring in tens of mouse and human proteins, binds RNA with higher affinity than DNA [127]. This motif from Prostate Tumor Overexpressed 1 (PTOV1) was shown to bind structured RNA, in agreement with the previously known property of basic tails to bind in the minor groove of double stranded DNA [116, 120]. Therefore, different types of disordered sequences may be able to recognise both RNA and DNA, albeit they may have preference for one.

## A role for disordered regions of RBPs in retaining RNA in membraneless granules

RNA processing and storage is often undertaken in the context of dynamic, membraneless organelles that vary in size, composition, and function. These organelles include the nucleolus, PML bodies, nuclear speckles and cajal bodies in the nucleus as well as P–bodies, stress and germ granules in the cytoplasm [128–130]. RNA granule formation relies on a spatiotemporally controlled transition from disperse “soluble” RNA and protein state to a condensed phase [131, 132]. The lack of a membrane allows a direct, dynamic and reversible exchange of components between the cytoplasm and the granule [131]. The rate of exchange and localisation of a protein within a granule can be markedly different depending on granule composition and the intrinsic properties of the protein [133–136]. RNA granules have roles in RNA localisation, stability, and translation, and perturbations in their homeostasis are hallmarks of numerous neurological disorders [137, 138].

Several recent studies have shown that disordered, low complexity regions in a number RBP have a capacity to form such granules [131, 139–141]. Different low complexity regions can promote RNA granule formation. For example, the disordered RG-rich sequence of LAF-1 (DDX3) was demonstrated to be both necessary and sufficient to promote P-granule formation in *C. elegans* [142]. Similarly, the RG/GR and FG/GF disordered tail of human RNA helicase DDX4 (aka Vasa) aggregates in vivo and *in vitro* [130]. Furthermore, the [G/S]Y[G/S] and poly glutamine (polyQ) motifs, which are present in a broad spectrum of RBPs, are necessary and sufficient to cause aggregation *in vitro* and in vivo [139, 140, 143–146]. It remains unclear how RNA binding by these sequences influences granule formation. Illustrating this idea, the RG-rich region of LAF-1 displays direct RNA–binding activity in addition to granule formation capacity. While RNA is not required for LAF-1 driven aggregation, it increases the internal dynamics of these LAF-1 droplets, making them more fluid [142]. In yeast, formation of P-body-like granules by the Lsm4 disordered region requires the presence of RNA [147]. Notably, the biophysical properties of RBP droplets can be altered by the presence of different RNA species [148]. A recent work reports an additional layer of complexity in the interplay between nucleic acids and granules. While single-stranded DNA is retained in DDX4-induced granules, double-stranded DNA is excluded, suggesting some degree of nucleic acid selectivity [130]. Given the biophysical similarities between DNA and RNA, it is possible that granules formed by analogous low complexity sequences also retain single stranded over double stranded RNA.

Interestingly, different types of low complexity sequences may help to form different types of aggregates and ways to embed RNA. A recent study showed that while low complexity sequences promote formation of both P-bodies and stress granules in yeast, these granules differ in their dynamic properties, P-bodies displaying more dynamic/fluid phase transition than more solid-like stress granules [147]. Granule structure, composition, and age can affect the biophysical properties of the granules [135, 136]. There is considerable overlap in the composition of different RNA granules [149]. Different proportions of such components may lead to the existence of a continuum of granule types with increasingly distinct physicochemical properties. In summary, it is clear that protein disorder has a role in formation of RNA granules. The importance of direct interaction between disordered regions and RNA in the context of granules remains to be determined.

## Modulating interactions between disordered regions and RNA

**Fig. 3:**
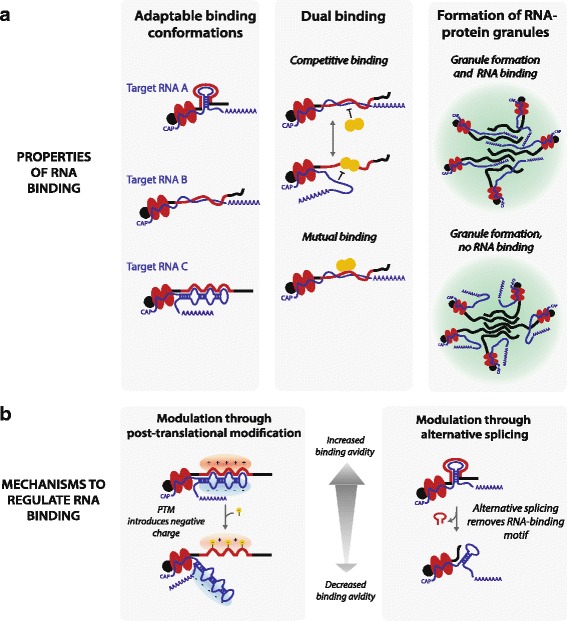
Models for properties of protein disorder in RNA binding. **a** Attributes of disordered protein regions in RNA interactions. **b** Post-translational modification and alternative splicing can modulate RNA-binding

Post-translational modifications can modulate protein’s interaction properties [150]. A number of disordered RNA-binding regions are known to be post-translationally modified (Table 1, Additional file 1: Figure S1) and some of these modifications can modulate RNA-binding affinity or cause local structural changes. For example, methylation of arginines of the RNA-binding RGG-box in the RNA export adapter ALY/REF reduces its affinity for RNA [151]. Arginine methylation of the RGG-box of the translational regulator FMRP affects interaction with target RNA as well as its polyribosome association [76, 152]. Also the RNA-binding basic patch of HIV protein Rev is methylated, which changes its interaction dynamics with its target RNA [153, 154]. Serine phosphorylation at the RNA-binding RS repeats of SRSF1 and DDX23 has been shown to induce a partial structuring of this region, which may impact their RNA-binding properties [36]. Assembly of RNA granules can also be modified by phosphorylation or methylation of the low complexity region [130, 155, 156]. In summary, occurrence of post-translational modifications at disordered regions represents an additional layer of regulation of RNA binding and metabolism (Fig. 3).

In other contexts, it is known that alternative splicing can alter the sequence and function of proteins. Several global analyses have reported that short, regulatory sequences such as sites for post-translational modifications and protein-protein interactions are often subjected to alternative splicing [157–159]. Could protein-RNA interactions be regulated in a similar manner? A number alternative isoform variants catalogued in large-scale studies affect RNA-binding disordered regions (Table 1, Additional file 1: Figure S1). As an illustrative example, alternative splicing of mouse ALY/REF selectively includes or excludes the RNA-binding RG-rich region, resulting in changes in its targeting to nuclear speckles and an increased cytoplasmic distribution [57, 60]. Alternative splicing affecting a region adjacent to the FMRP RGG-box influences the protein’s RNA-binding activity [160], reduces its ability to associate with polyribosomes [161], and can also impact RGG-box methylation [162]. Another splice isoform results in ablation of the RGG-box as a result of a translational frameshift, which induces nuclear distribution of the protein [163]. Also RNA granule formation can be differentially regulated in different tissues though selective splicing isoforms including or excluding granule-forming low complexity regions [164]. Although to our knowledge a genome-wide analysis is still outstanding, these anecdotal examples hint that alternative splicing may operate to alter disorder-RNA interactions in a global manner (Fig. 3).

RNA-binding activity can also be modulated by competitive or cooperative interactions (Table 1, Fig. 3). The ability of some disordered regions to mediate protein-protein or protein-DNA interactions in addition to protein-RNA interactions could provide additional means to regulate RBP function. Therefore, disordered regions, although neglected for decades, have the potential to emerge as dynamic mediators of RNA biology.

## Conclusions

### Why disorder?

We have discussed the contribution of RS-, RG-, and K/R-rich, disordered regions to RNA interactions, and given examples of how they participate in co- and post-transcriptional regulation of RNA metabolism; how defects in these interactions can lead to disease; and how disorder in RBPs can be utilised by viruses during their infection cycle. Disordered regions are emerging as malleable, often multifunctional RNA-binding modules whose interactions with RNA range from non-specific to highly selective with defined target sequence or structural requirements (Fig. 3). How specificity is generated for RNA sequences or structures by disordered RNA-binding regions remains to be determined. Specific interactions with defined RNA structures have been demonstrated in some instances. It seems likely that specificity and affinity can be increased by oligomerisation and through the combinatorial modular architecture of RBPs. Disorder may be a spatially cost-effective way of encoding general affinity for RNA and/or structural flexibility to enable co-folding in presence of the target RNA, thus allowing multiple binding solutions not easily achievable by structured domains. Because disorder-mediated interaction with RNA typically relies on physicochemical properties of short stretches of sequence, they can be easily regulated through post-translational modifications. Disorder may also endow special properties such as propensity to form RNA granules and interact with other RBPs. Here we have grouped the RNA - binding disordered regions based on their amino acid composition. It is possible that other functional RNA-binding motifs with unobvious sequence patterns remain to be discovered.

## Outstanding questions

Much remains to be learnt about disorder-mediated protein-RNA interactions. How do disordered regions interact with RNA? How many functionally relevant disorder-RNA interactions exist? Can more refined motifs be identified among the different classes of RNA-binding disordered regions? Are there further subclasses of motifs within RS-, RG-, basic, and other RNA-binding disordered regions with distinct binding characteristics? How is RNA binding regulated post-translationally, by alternative splicing, or by competitive interactions with other biomolecules? How do mutations in disordered regions involved in RNA binding cause disease? Fundamental principles of disorder-RNA interactions are likely to have close parallels to what has been elucidated for protein-protein and protein-DNA interactions, where disorder-mediated regulation has received much more attention over the past decade [111, 165–170]. Thus, the conceptual framework to start answering questions on the role of protein disorder in RNA binding already has a firm foundation.

## Concluding statement

Structure-to-function paradigm [171] has persisted long in the field of protein-RNA interactions. In this review, we have highlighted the important role that disordered regions play in RNA binding and regulation. Indeed, the recent studies on mammalian RNA-binding proteomes place disordered regions at the centre of the still expanding universe of RNA-protein interactions. It is thus time to embark on a more systematic quest of discovery for the elusive functions of disordered protein regions in RNA biology.

## Additional file

Additional file 1: Figure S1. Properties of RNA-binding, disordered proteins. Disorder and charge profiles for proteins listed in Table 1. The disordered, RNA-binding regions (RBR) are marked in blue in the left panel, and their sequence given in the right panel. Amino acid sequence, GO terms, and annotations for protein domains, isoforms, and post-translational modifications (PTMs) were extracted from UniProt [250]. Disorder was calculated using IUPred [172] using default values. Score above 0.4 indicates the region is intrinsically disordered (in physiological conditions). Charge was calculated using EMBOSS charge [251] using default values. PTMs: A, acetylation; M, methylation; P, phosphorylation; O, other. See Table 1 for literature references for each protein. (PDF 904 kb)

## Abbreviations

ARM: arginine-rich motif
dsRBD: double-stranded RNA-binding domain
GAR repeat: glycine-arginine-rich repeat
KH domain: K-homology domain
RBD: RNA-binding domain
RBP: RNA-binding protein
RGG-box: arginine-glycine-glycine-box
RRM: RNA recognition motif
RS repeat: arginine-serine repeat
