# Supplementary material for: The new (dis)order in RNA regulation
Source: Cell Commun Signal. 2016 Apr 6;14:9. doi: 10.1186/s12964-016-0132-3 (PMC4822317; doi:10.1186/s12964-016-0132-3)
Supplement: Additional file 1: Figure S1. — Properties of RNA-binding, disordered proteins. Disorder and charge profiles for proteins listed in Table 1. The disordered, RNA-binding regions (RBR) are marked in blue in the left panel, and their sequence given in the right panel. Amino acid sequence, GO terms, and annotations for protein domains, isoforms, and post-translational modifications (PTMs) were extracted from UniProt [250]. Disorder was calculated using IUPred [172] using default values. Score above 0.4 indicates the region is intrinsically disordered (in physiological conditions). Charge was calculated using EMBOSS charge [251] using default values. PTMs: A, acetylation; M, methylation; P, phosphorylation; O, other. See Table 1 for literature references for each protein. (PDF 904 kb) [file 12964_2016_132_MOESM1_ESM.pdf]

Additional Figure 1. Properties of RNA-binding, disordered proteins. Disorder and charge profiles for proteins listed in Table 1. The disordered, RNA-binding regions (RBR) are marked in blue in the left panel, and their sequence given in the right panel. Amino acid sequence, GO terms, and annotations for protein domains, isoforms, and post-translational modifications (PTMs) were extracted from UniProt [250]. Disorder was calculated using IUPred [172] using default values. Score above 0.4 indicates the region is intrinsically disordered (in physiological conditions). Charge was calculated using EMBOSS charge [251] using default values. PTMs: A, acetylation; M, methylation; P, phosphorylation; O, other. See Table 1 for literature references for each protein.

SRSF1

Serine/arginine–rich splicing factor 1

Alternative–splicing factor 1; Splicing factor, arginine/serine–rich 1; pre–mRNA–splicing factor SF2, P33 subunit

(Q07955)

(Homo sapiens)

PROTEIN

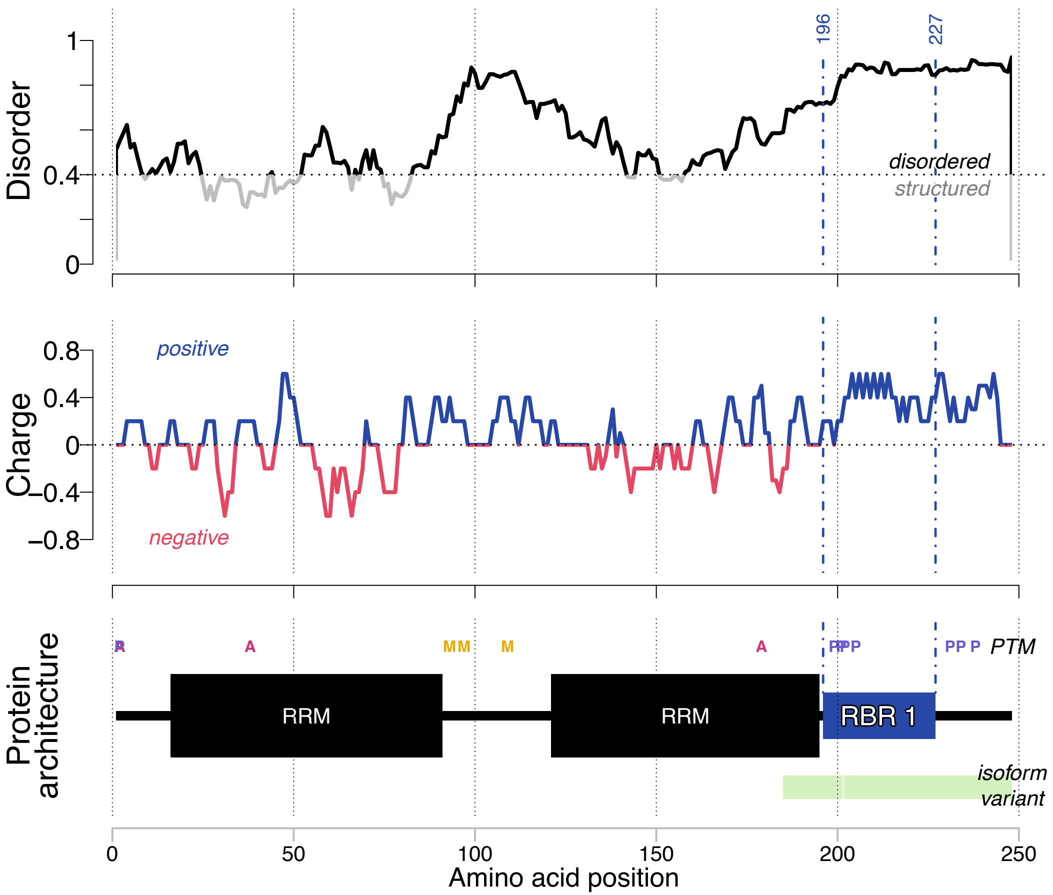

RNA BINDING DISORDERED PEPTIDE

RBR 1

196 GPRSPSYGRSRSRSRSRSRSRSNSRSRSYS 227

GO biological processes

cardiac muscle contraction, gene expression, in utero embryonic development, mRNA 3'–end p rocessing, mRNA 5'–splice site recognition, mRNA export from nucleus, mRNA processing, mRN A splice site selection, mRNA splicing, via spliceosome, regulation of mRNA splicing, via spliceosome, regulation of mRNA stability, regulation of transcription, DNA–templated, reg...

GO molecular functions

mRNA binding, nucleotide binding, poly(A) RNA binding, RNA binding

GO cellular compartments

catalytic step 2 spliceosome, cytoplasm, extracellular exosome, nuclear speck, nucleoplasm , nucleus

U2AF2

Splicing factor U2AF 65 kDa subunit

U2 auxiliary factor 65 kDa subunit; U2 snRNP auxiliary factor large subunit
(P26368)

(Homo sapiens)

PROTEIN

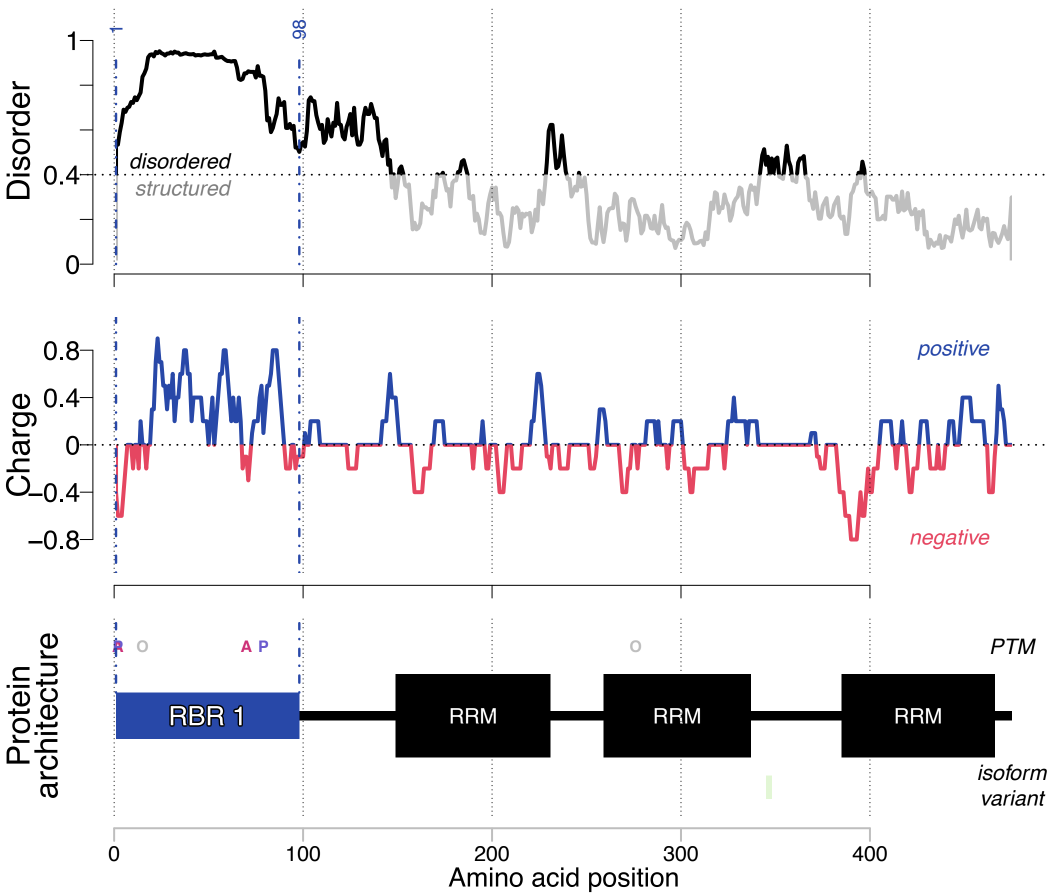

RNA BINDING DISORDERED PEPTIDE

RBR 1

1 MSDFDEFERQLNENKQERDKENRHRKRSHSRSRSRDRKRRSRSRDRRNRDQRSASRDRRR
RSKPLTRGAKEEHGGLIRSPRHEKKKKVRKYWDVPPPG 98

GO biological processes

gene expression, mRNA 3'–end processing, mRNA export from nucleus, mRNA processing, mRNA s plicing, via spliceosome, negative regulation of mRNA splicing, via spliceosome, positive regulation of protein targeting to mitochondrion, positive regulation of RNA splicing, reg ulation of mitochondrion degradation, RNA splicing, termination of RNA polymerase II trans...

GO molecular functions

enzyme binding, nucleotide binding, poly(A) RNA binding

GO cellular compartments

nuclear speck, nucleoplasm, spliceosomal complex

Note:
The primary sequence of RS repeat is not essential for functionality: heterologous RS domains present in functionally unrelated splicing factors can be used as substitutes. Replaci ng the region (1–94) with 7 RS repeats is sufficient to support splicing. Serine not required for splicing activity. Arg can be replaced by Lys but not Ala.

NKAP

NF–kappa–B–activating protein

(Q8N5F7)

(Homo sapiens)

PROTEIN

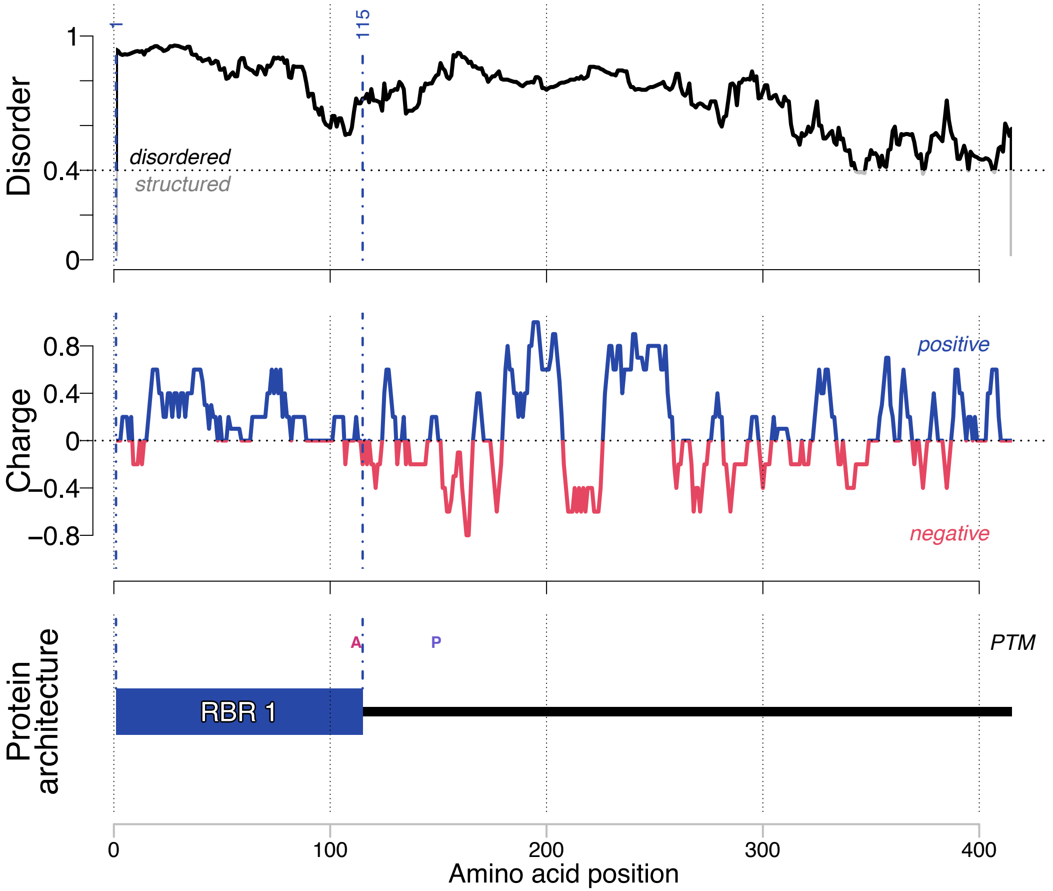

RNA BINDING DISORDERED PEPTIDE

RBR 1

1 MAPVSGSRSPDREASGSGGRRRSSSKSPKPSKSARSPRGRRSRSHSCSRSGDRNGLTHQL
GGLSQGSRNQSYRSRSRSRSRERPSAPRGIPFASASSSVYYGSYSRPYGSDKPWP 115

GO biological processes

granulocyte differentiation, hematopoietic stem cell proliferation, negative regulation of transcription from RNA polymerase II promoter, negative regulation of transcription, DNA–templated, Notch signaling pathway, positive regulation of alpha–beta T cell differentiati on, positive regulation of protein targeting to mitochondrion, stem cell maintenance, T ce...

GO molecular functions

chromatin binding, chromatin DNA binding, poly(A) RNA binding

GO cellular compartments

cytoplasm, nucleoplasm

Note:
An additional domain, DUF 926, contributes to RNA binding in addition to the region highlighted. This protein is overall quite disordered (mean 0.73), contains a basic, Lys rich, d isordered central region (required for nuclear speckle localisation).

Nucleocapsid protein

Nucleoprotein

Nucleocapsid protein
(P59595)

(Human SARS coronavirus)

PROTEIN

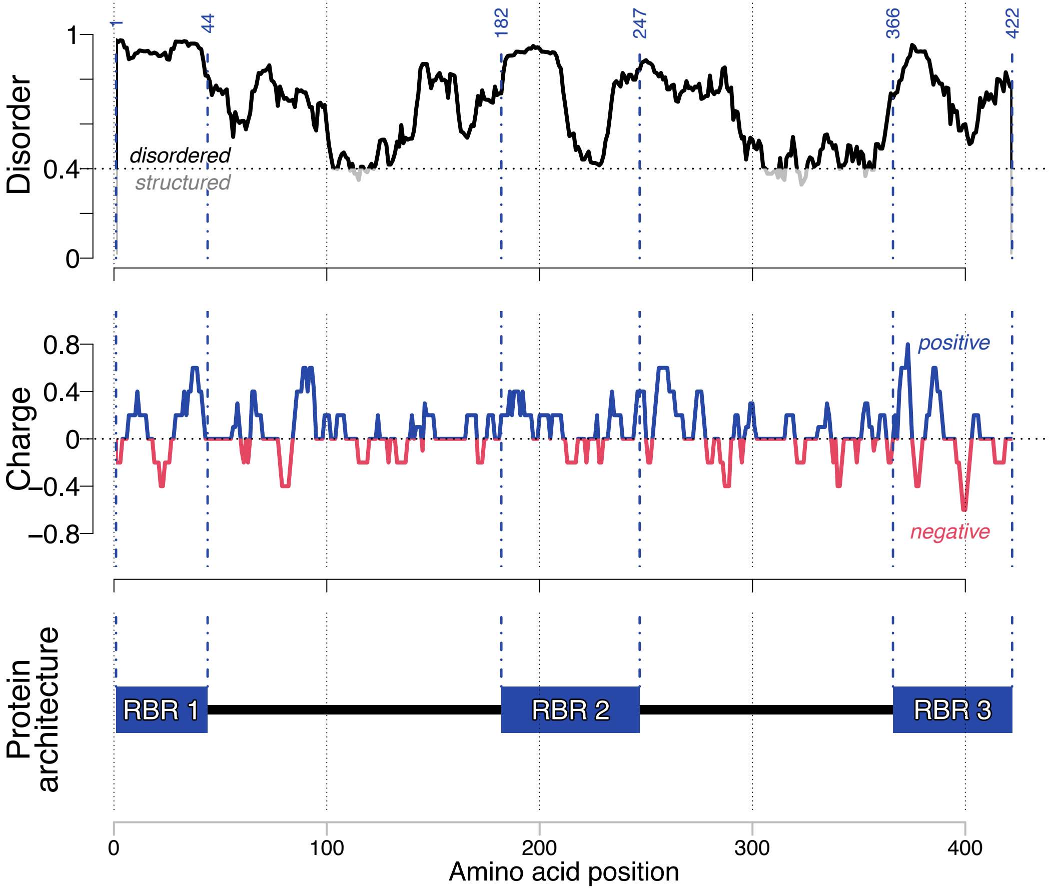

RNA BINDING DISORDERED PEPTIDE

RBR 1

1 MSDNGPQSNQRSAPRITFGGPTDSTDNNQNGGRNGARPKQRRPQ 44

RBR 2

182 QASSRSSSRSRGNSRNSTPGSSRGNSPARMASGGGETALALLLLDRLNQLESKVSGKGQQ
QQGQTV 247

RBR 3

366 PTEPKKDKKKKTDEAQPLPQRQKKQPTVTLLPAADMDDFSRQLQNSMSGASADSTQA 422

GO biological processes

–

GO molecular functions

RNA binding

GO cellular compartments

host cell endoplasmic reticulum−Golgi intermediate compartment, host cell Golgi apparatus, host cell perinuclear region of cytoplasm, viral nucleocapsid

Note:
This protein has 5 RNA−binding regions, of which two are structured. They are flanked by three disordered RNA binding regions.

ALYREF

Aly/REF export factor 2

Alyref; RNA and export factor–binding protein 2
(Q9JJW6)

(Mus musculus)

PROTEIN

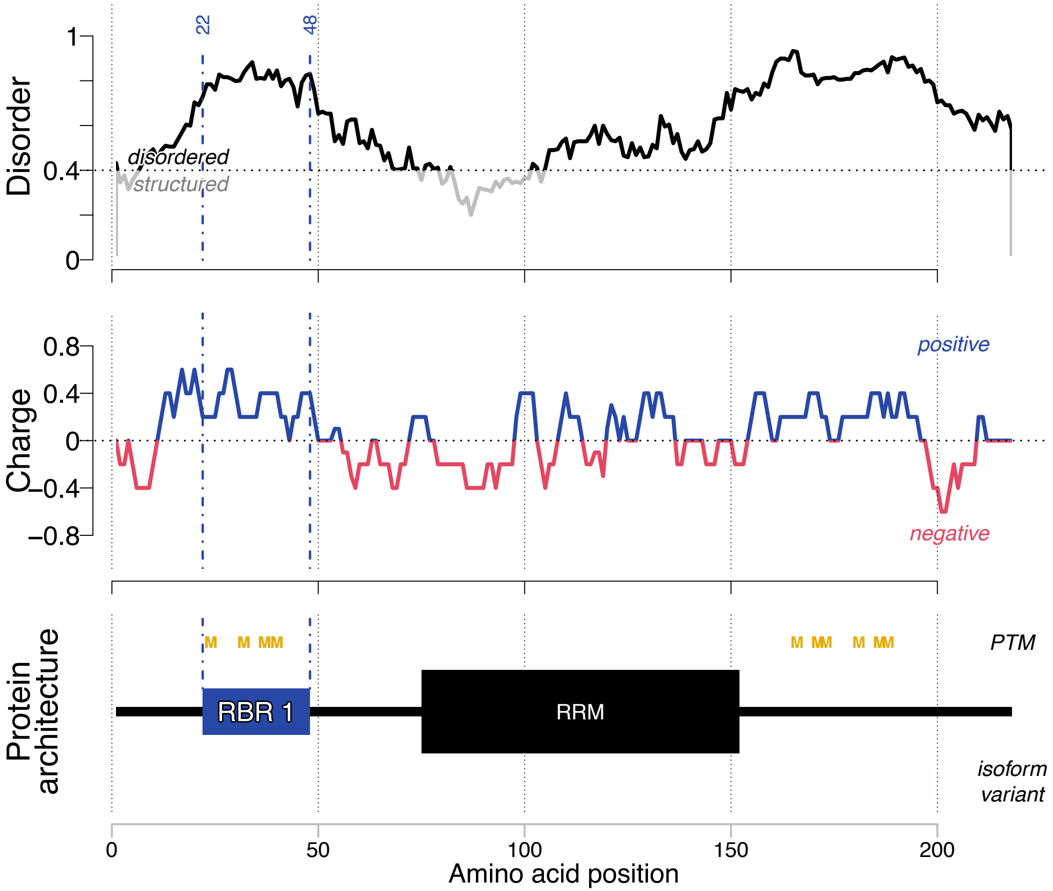

RNA BINDING DISORDERED PEPTIDE

RBR 1

22 VNRGGGPRRNRPAIARGGRNRPAPYSR 48

GO biological processes

mRNA export from nucleus, mRNA processing, mRNA transport, RNA splicing

GO molecular functions

mRNA binding, nucleotide binding, RNA binding, single−stranded DNA binding

GO cellular compartments

cytoplasm, spliceosomal complex

Aven
Cell death regulator Aven

(Q9NQS1)

(Homo sapiens)

PROTEIN

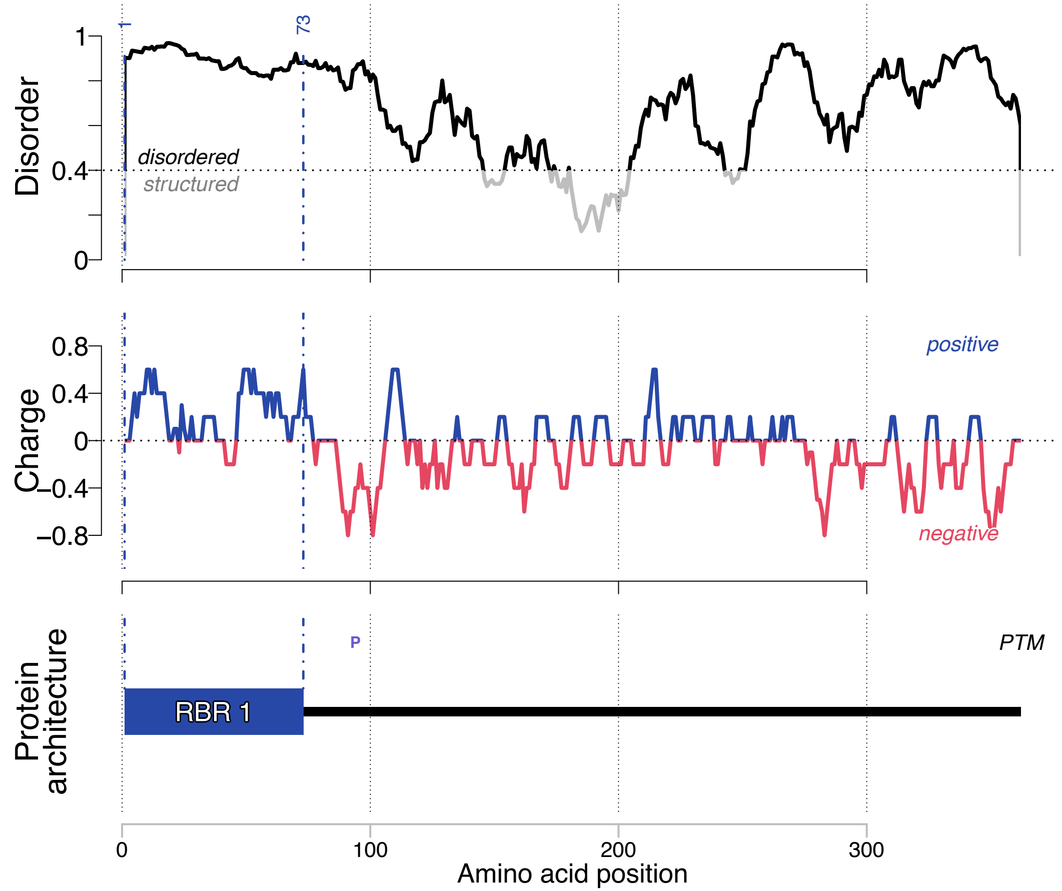

RNA BINDING DISORDERED PEPTIDE

RBR 1

1 MQAERGARGGRGRRPGRGRPGGDRHSERPGAAAAVARGGGGGGGGDGGGRRGRGRGRGFR GARGGRGGGGAPR 73

GO biological processes

apoptotic process, negative regulation of apoptotic process

GO molecular functions

–

GO cellular compartments

endomembrane system, intracellular, membrane

Note:
Negative regulation of apoptosis. Aven expression is increased in certain leukemias and osteosarcomas.

Caprin−1

Caprin−1

Cell cycle−associated protein 1; Cytoplasmic activation− and proliferation−associated protein 1; GPI−anchored membrane protein 1; GPI−anchored protein p137...
(Q14444)

(Homo sapiens)

PROTEIN

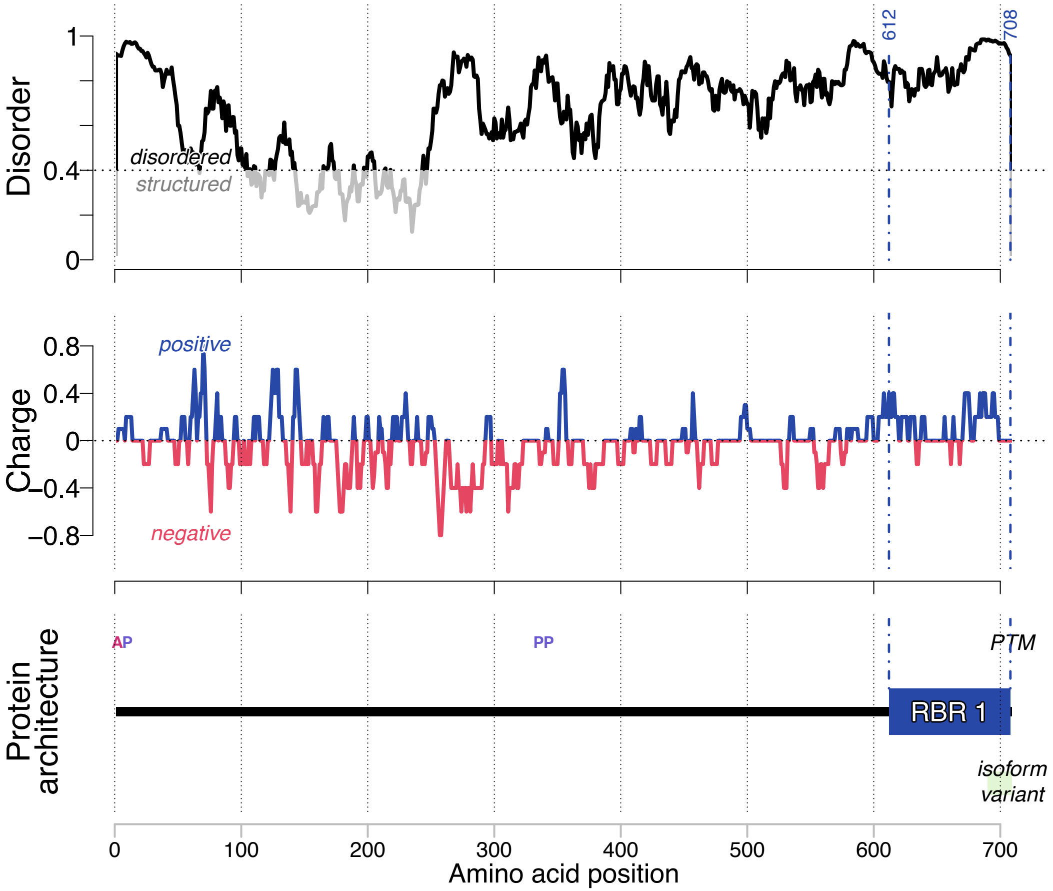

RNA BINDING DISORDERED PEPTIDE

RBR 1

612 RGGSRGARGLMNGYRGPANGFRGGYDGYRPSFSNTPNSGYTQSQFSAPRDYSGYQRDGYQ
QNFKRGSGQSGPRGAPRGRGGPPRPNRGMPQMNTQQV 708

GO biological processes

negative regulation of translation, positive regulation of dendrite morphogenesis, positiv
e regulation of dendritic spine morphogenesis

GO molecular functions

poly(A) RNA binding, RNA binding

GO cellular compartments

cytoplasm, cytoplasmic mRNA processing body, cytoplasmic stress granule, cytosol, dendrite
, integral component of plasma membrane, membrane

Note:
Study by Solomon et al. found that mutating the Args of the three RGGs present in the human sequence into AGGs strongly reduced RNA binding. The Xenopus sequence represents the fir
st part in the human sequence studied. Shiina et al. identified an additional, Lys−rich N−terminal sequence in the Xenopus protein as RNA−binding.

Caprin−1

RNA granule protein 105

(Q75T15)

(Xenopus laevis)

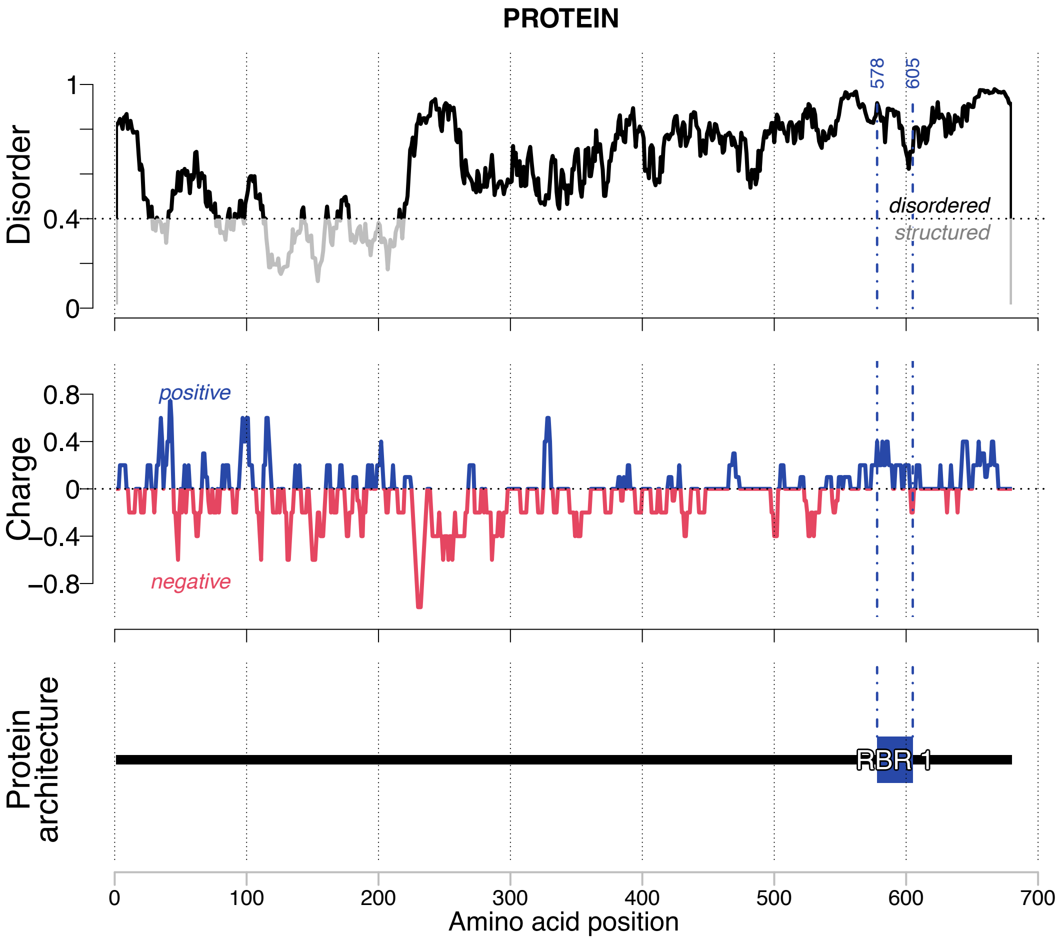

RNA BINDING DISORDERED PEPTIDE

RBR 1

578 RGMARGGQRGNRGMMNGYRGQSNGFRGG 605

GO biological processes

–

GO molecular functions

–

GO cellular compartments

–

Note:

Study by Solomon et al. found that mutating the Args of the three RGGs present in the human sequence into AGGs strongly reduced RNA binding. The Xenopus sequence represents the fir st part in the human sequence studied. Shiina et al. identified an additional, Lys−rich N−terminal sequence in the Xenopus protein as RNA−binding.

DDX4

Probable ATP–dependent RNA helicase DDX4

DEAD box protein 4; Vasa homolog

(Q9NQI0)

(Homo sapiens)

PROTEIN

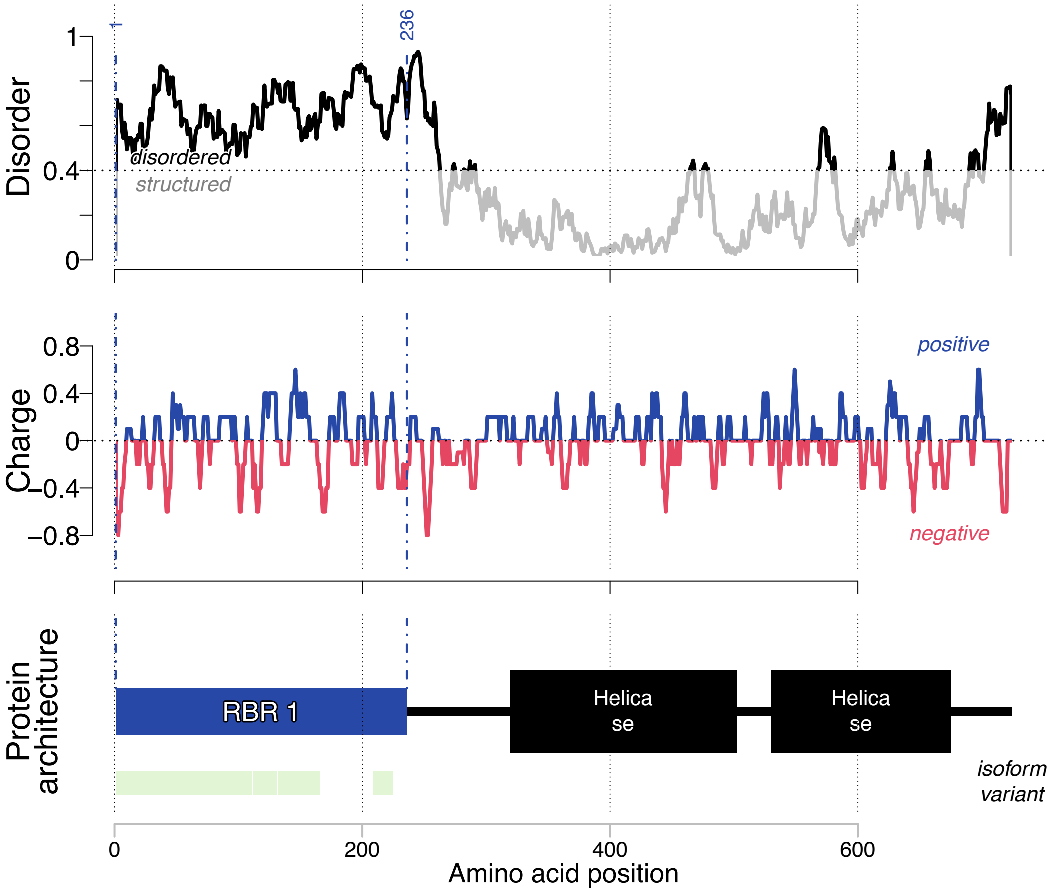

RNA BINDING DISORDERED PEPTIDE

RBR 1

1 MGDEDWEAEINPHMSSYVPIFEKDRYSGENGDNFNRTPASSSEMDDGPSRRDHFMKSGFA
SGRNFGNRDAGECNKRDNTSTMGGFGVGKSFGNRGFSNSRFEDGDSSGFWRESSNDCEDN
PTRNRGFSKRGGYRDGNNSEASGPYRRGGRGSFRGCRGGFGLGSPNNDLDPDECMQRTGG
LFGSRRPVLSGTGNGDTSQSRSGSGSERGGYKGLNEEVITGSGKNSWKSEAEGGES 236

GO biological processes

gene expression, male meiosis I, multicellular organismal development, piRNA metabolic process, regulation of protein localization, RNA secondary structure unwinding, sperm motility, spermatogenesis

GO molecular functions

ATP binding, ATP–dependent RNA helicase activity, nucleic acid binding

GO cellular compartments

chromatoid body, cytoplasm, nucleus, P granule, perinuclear region of cytoplasm, pi–body, piP–body

Note:

Involved in assembly and maintenance of certain types of RNA granules. Nott et al. (2015) assessed Ddx4 binding to nucleotides using single and double stranded DNA. The sequence may bind RNA in a similar manner. GF/GF repeats are common in this sequence. The disordered region drives formation of DDX4 granules, methylation destabilises these droplets.

EWS

RNA–binding protein EWS

EWS oncogene; Ewing sarcoma breakpoint region 1 protein
(Q01844)

(Homo sapiens)

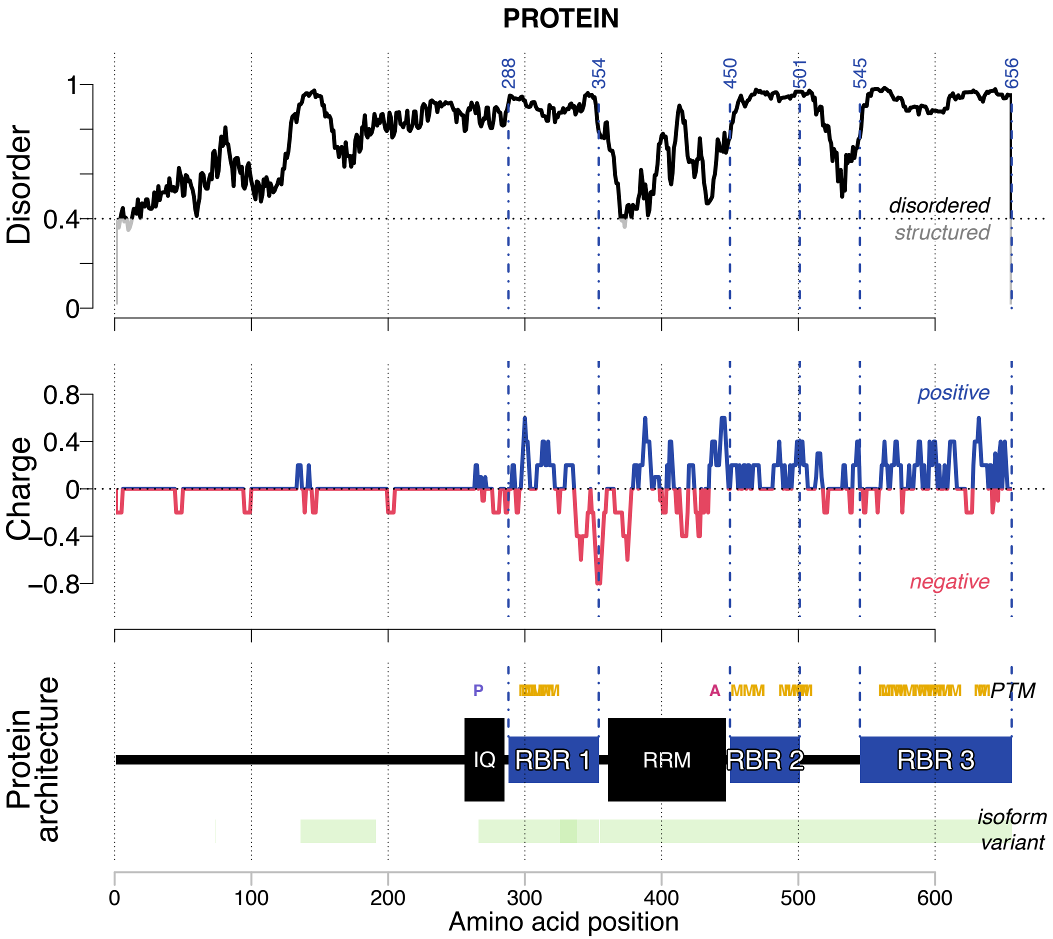

RNA BINDING DISORDERED PEPTIDE

RBR 1

288 PGENRSMSGPDNRGRGRGGFDRGGMSRGGRGGGRGGMGSAGERGGFNKPGGPMDEGPDLD
LGPPVDP 354

RBR 2

450 PMNSMRGGLPPREGRGMPPPLRGGPGGPGGPGGPMGRMGGRGGDRGGFPPRG 501

RBR 3

545 APKPEGFLPPPFPPPGGDRGRGGPGGMRGGRGGLMDRGGPGGMFRGGRGGDRGGFRGGRG
MDRGGFGGGRRGGPGGPPGPLMEQMGGRRGGRGGPGKMDKGEHRQERRDRPY 656

GO biological processes

regulation of transcription, DNA–templated, transcription, DNA–templated

GO molecular functions

identical protein binding, nucleotide binding, poly(A) RNA binding, RNA binding, zinc ion binding

GO cellular compartments

cytoplasm, nucleolus, nucleoplasm, nucleus, plasma membrane

Note:
Regulates FAS/CD95–mediated apoptosis. Note that PG/G is also common in RGG2 and RGG3.

FMRP

Fragile X mental retardation protein 1

(Q06787)

(Homo sapiens)

PROTEIN

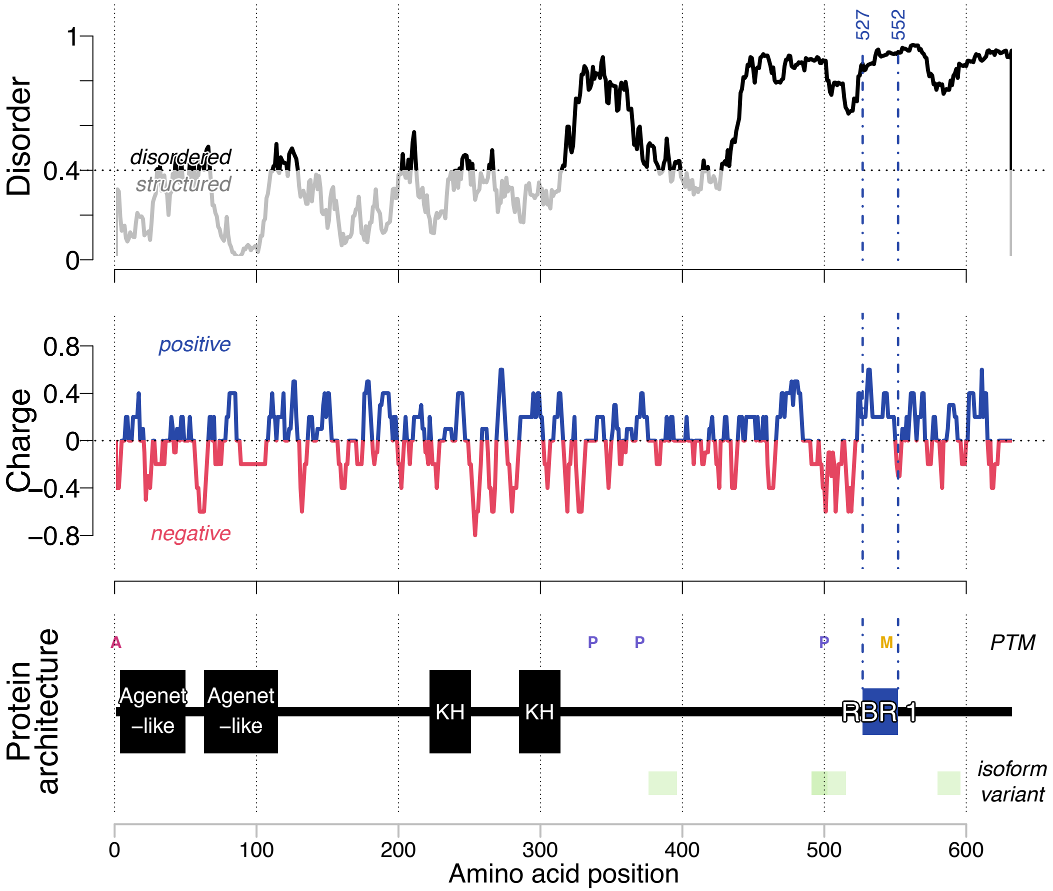

RNA BINDING DISORDERED PEPTIDE

RBR 1

527 RRGDGRRRGGGGRGQGGRGRGGGFKG 552

GO biological processes

central nervous system development, mRNA transport, negative regulation of translation, ne
gative regulation of translational initiation

GO molecular functions

mRNA binding, poly(A) RNA binding, RNA binding

GO cellular compartments

cytoplasm, cytoplasmic ribonucleoprotein granule, cytoplasmic stress granule, dendrite, me
mbrane, mRNA cap binding complex, nucleolus, nucleoplasm, nucleus, polysome

Note:
Different arginines are involved in contact with different RNAs. Central part of the RGG box may form a beta–turn. Target RNA may retain structural flexibility. Altered expression of this protein can lead to fragile X syndrome, fragile X tremor/ataxia syndrome, premature ovarian failure 1, and autism.

FUS

RNA–binding protein FUS

75 kDa DNA–pairing protein; Oncogene FUS; Oncogene TLS; POMp75; Translocated in liposarcoma protein
(P35637)

(Homo sapiens)

PROTEIN

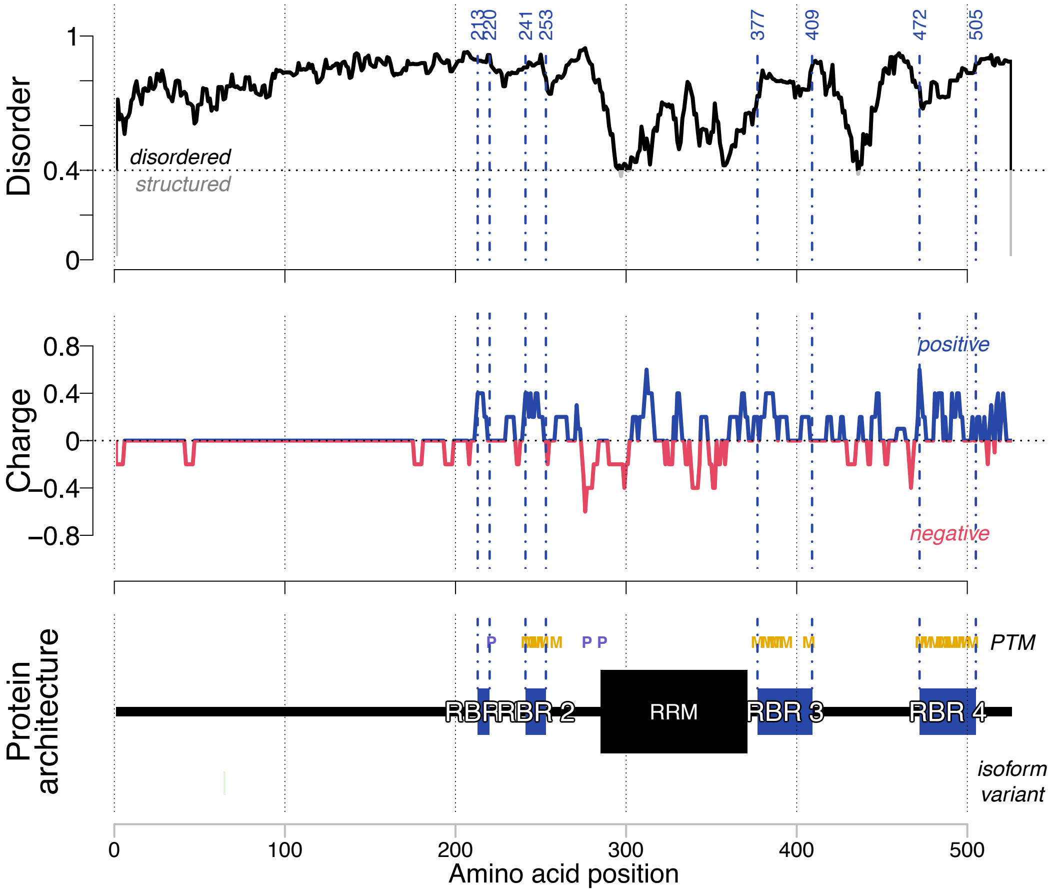

RNA BINDING DISORDERED PEPTIDE

RBR 1
213 RGGRGRGG 220

RBR 2
241 PRGRGGGRGGRGG 253

RBR 3
377 RGGGNGRGGRGRGGPMGRGGYGGGGSGGGGRGG 409

RBR 4
472 RRGGRGGYDRGGYRGRGGDRGGFRGGRGGGDRGG 505

GO biological processes

cellular response to calcium ion, gene expression, mRNA splicing, via spliceosome, regulat ion of nucleic acid–templated transcription, RNA splicing

GO functions and cellular compartments

FUNCTIONS:DNA binding, identical protein binding, nucleotide binding, poly(A) RNA binding, RNA binding, transcription coactivator activity, zinc ion binding. COMPARTMENTS:dendritic spine head, nucleoplasm, nucleus, perikaryon, perinuclear region of cytoplasm, polysome.

Note:
The highlighted RG–regions are estimates as specific sequence information could not be retrieved from original publications. This gene is linked to amyotrophic lateral sclerosis an d frontotemporal lobular degeneration.

hnRNPU

Heterogeneous nuclear ribonucleoprotein U

Scaffold attachment factor A; p120; pp120
(Q00839)

(Homo sapiens)

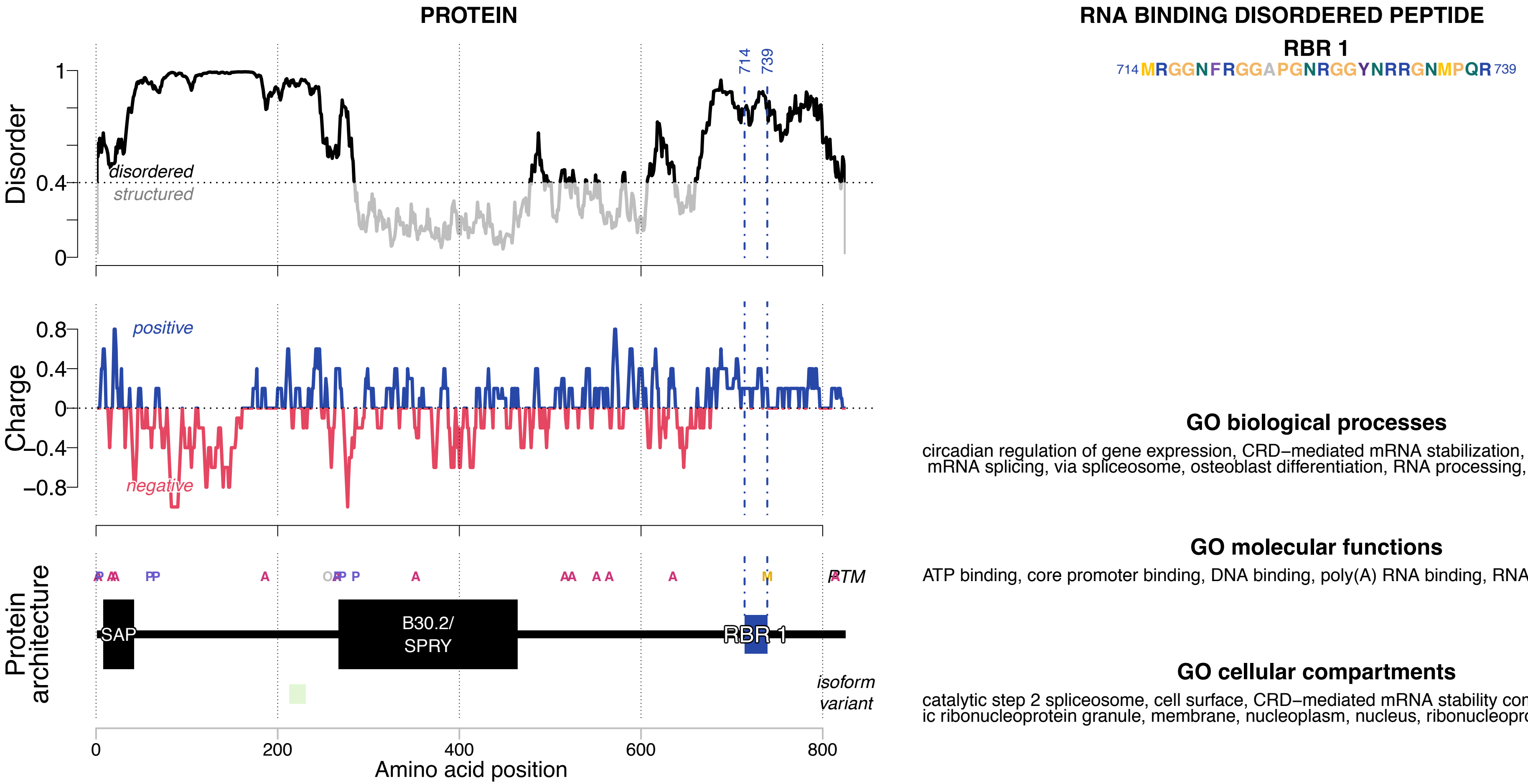

GO biological processes

circadian regulation of gene expression, CRD–mediated mRNA stabilization, gene expression, mRNA splicing, via spliceosome, osteoblast differentiation, RNA processing, RNA splicing

GO molecular functions

ATP binding, core promoter binding, DNA binding, poly(A) RNA binding, RNA binding

GO cellular compartments

catalytic step 2 spliceosome, cell surface, CRD–mediated mRNA stability complex, cytoplasmic ribonucleoprotein granule, membrane, nucleoplasm, nucleus, ribonucleoprotein complex

ICP27

mRNA export factor

Immediate−early protein IE63; Infected cell protein 27; VMW63
(P10238)

(Human herpesvirus 1 (strain 17))

PROTEIN

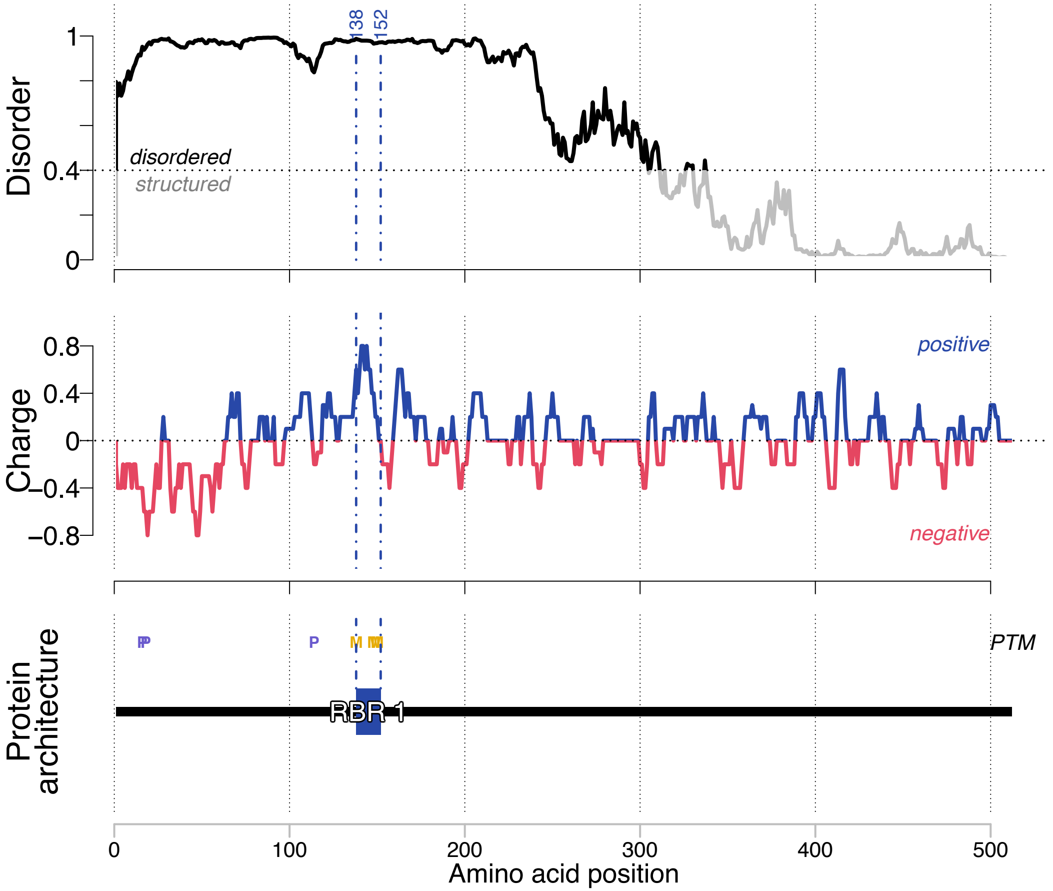

RNA BINDING DISORDERED PEPTIDE

RBR 1

138 RGGRRGRRRGRGRGG 152

GO biological processes

activation by virus of host NF−kappaB transcription factor activity, modulation by virus of host G1/S transition checkpoint, suppression by virus of host mRNA processing, transcription, DNA−templated

GO molecular functions

metal ion binding, RNA binding

GO cellular compartments

host cell cytoplasm, host cell nucleus

Note:
Remains unstructured when bound to RNA.

LAF1

LAF−1, isoform b; Putative DEAD−box RNA helicase

(D0PV95)

(Caenorhabditis elegans)

PROTEIN

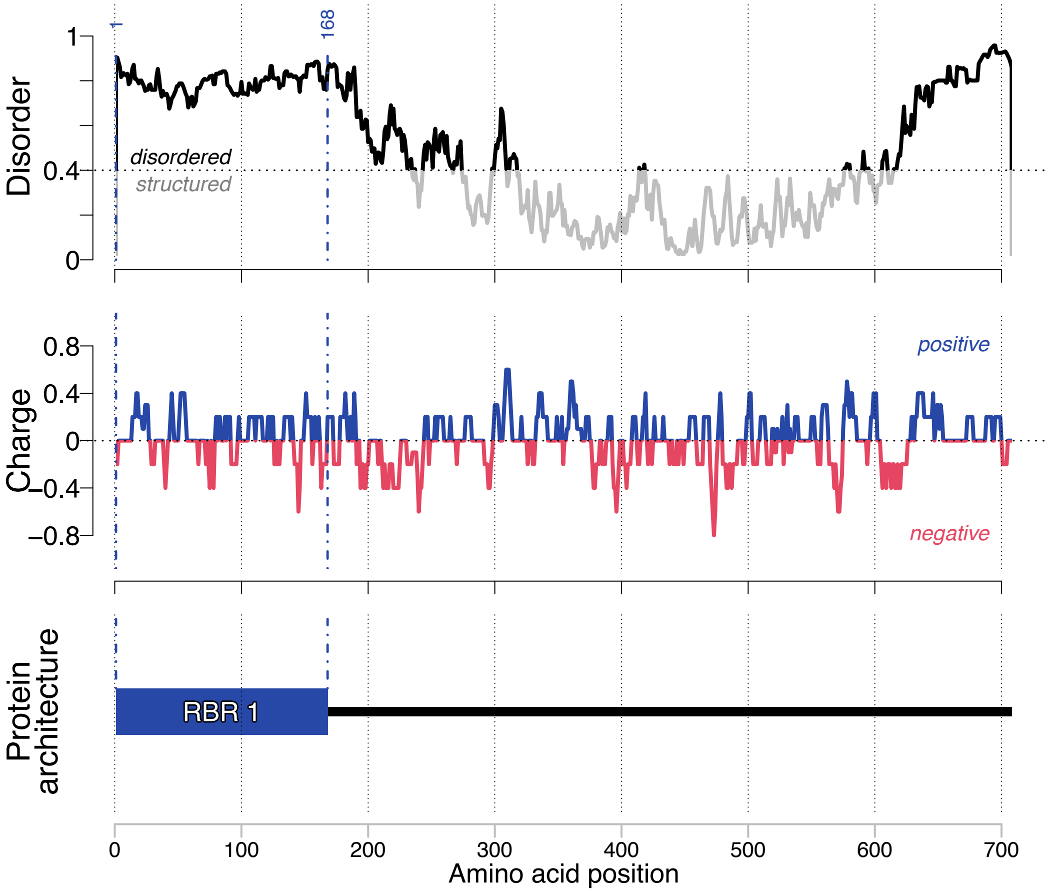

RNA BINDING DISORDERED PEPTIDE

RBR 1

1 MESNQSNNGGSGNAALNRGGRYVPPHLRGGDGGAAAAASAGGDDRRGGAGGGGYRRGGGN
SGGGGGGGYDRGYNDNRDDRDNRGGSGGYGRDRNYEDRGYNGGGGGGGNRGYNNNRGGGG
GGYNRQDRGDGGSSNFSRGGYNNRDEGSDNRGSGRSYNNDRRDNGGDG 168

GO biological processes

chromosome segregation, regulation of gene expression, RNA secondary structure unwinding, translational initiation

GO molecular functions

ATP binding, ATP−dependent RNA helicase activity, nucleic acid binding

GO cellular compartments

cytoplasm, P granule

Note:
The RNA binding, N−terminal region is also necessary and sufficient for formation of LAF−1 RNA granules. Note that GY and SS pairs are common in this sequence, as are stretches of G.

NXF1

Nuclear RNA export factor 1

Tip−associated protein; Tip−associating protein; mRNA export factor TAP

(Q9UBU9)

(Homo sapiens)

PROTEIN

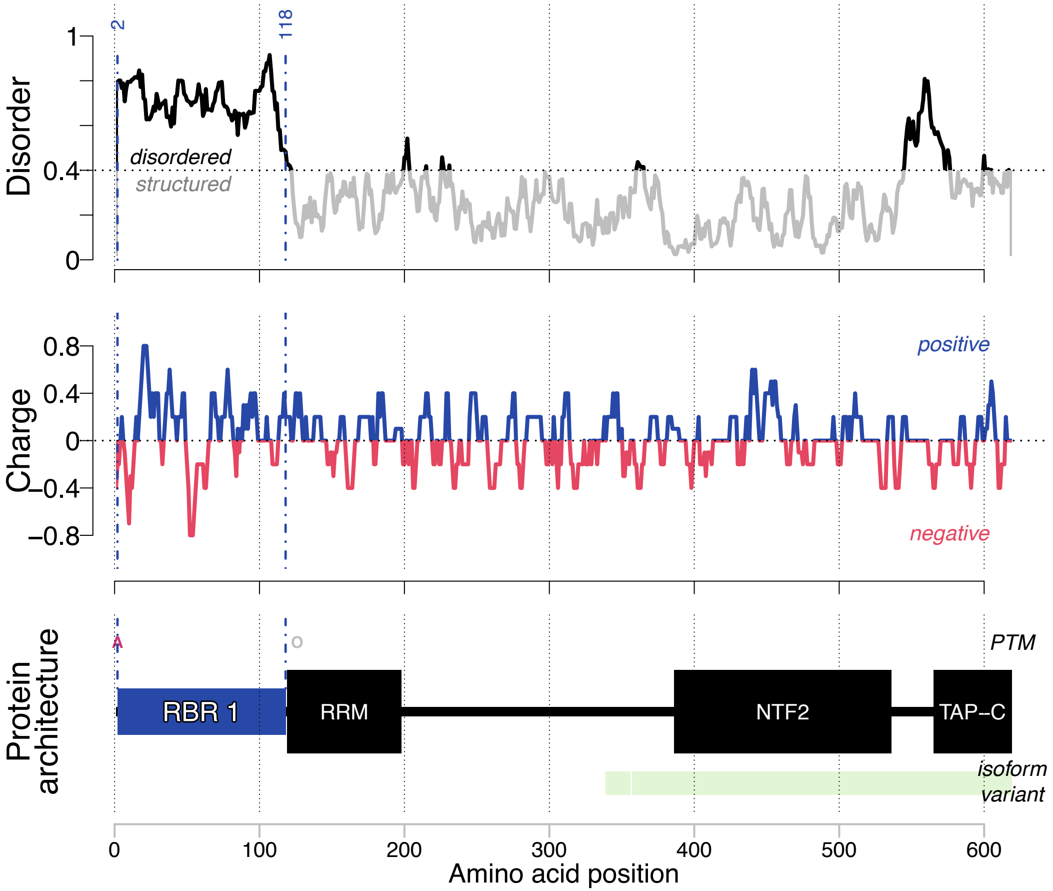

RNA BINDING DISORDERED PEPTIDE

RBR 1

2 ADEGKSYSEHDDERVNFPQRKKKGRGPFRWKYGEGNRRSGRGGSGIRSSRLEEDDGDVAM
SDAQDGPRVRYNPYTTRPNRRGDTWHDRDRIHVTVRRDRAPPERGGAGTSQDGTSKN 118

GO biological processes

gene expression, mRNA export from nucleus, viral process

GO molecular functions

nucleotide binding, poly(A) RNA binding, single−stranded RNA binding

GO cellular compartments

cytosol, nuclear speck, nucleoplasm, nucleus

Note:
The disordered RNA binding region is further divided into N−terminal 'minor RNA binding' and C−terminal 'major RNA binding' regions. Mutations of 10 Args in major region drasticall
y reduces RNA binding.

Nucleolin

Nucleolin

Protein C23
(P08199)

(Mesocricetus auratus)

PROTEIN

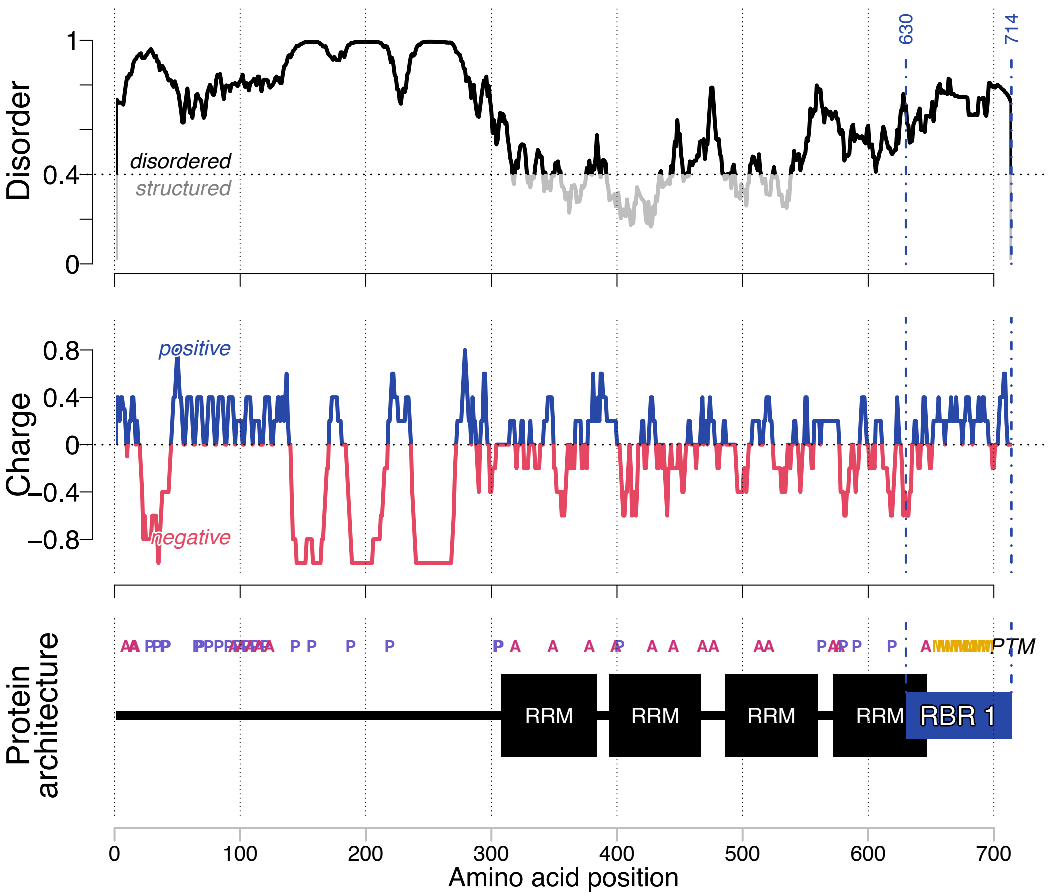

RNA BINDING DISORDERED PEPTIDE

RBR 1

630 MEDGEIDGNKVTLDWAKPKGEGGFGGRGGGRGGFGGRGGGRGGGRGGFGGRGRGGFGGRG
GFRGGRGGGGGGGDFKPQGKKTKFE 714

GO biological processes

–

GO molecular functions

nucleotide binding, RNA binding, telomeric DNA binding

GO cellular compartments

cytoplasm, nucleolus, ribonucleoprotein complex

RBMX

RNA–binding motif protein, X chromosome

Glycoprotein p43; Heterogeneous nuclear ribonucleoprotein G
(P38159)

(Homo sapiens)

PROTEIN

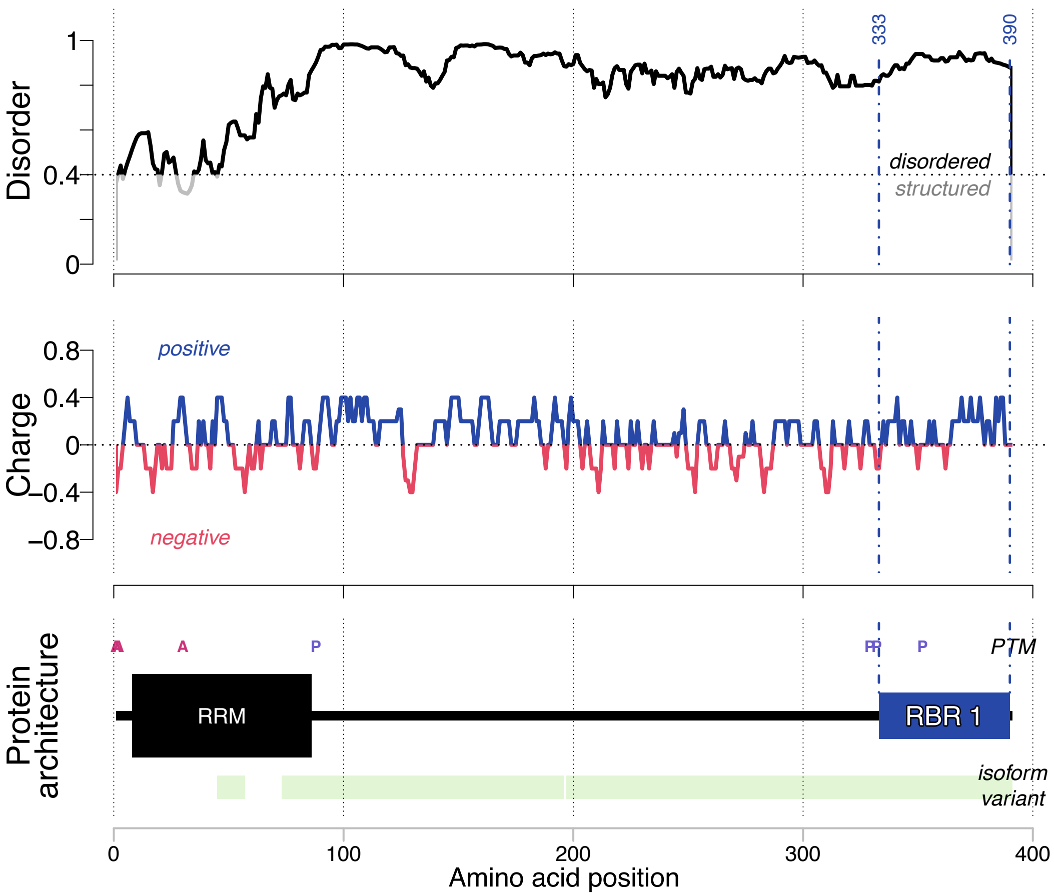

RNA BINDING DISORDERED PEPTIDE

RBR 1

333 DLYSSGRDRVGRQERGLPPSMERGYPPPRDSYSSSSRGAPRGGGRGGSRSDRGGGRSR 390

GO biological processes

cellular response to interleukin−1, gene expression, membrane protein ectodomain proteolysis, mRNA splice site selection, mRNA splicing, via spliceosome, negative regulation of mRNA splicing, via spliceosome, osteoblast differentiation, positive regulation of mRNA splicing, via spliceosome, positive regulation of transcription from RNA polymerase II promoter...

GO molecular functions

chromatin binding, core promoter binding, mRNA binding, nucleotide binding, poly(A) RNA binding, RNA binding, single−stranded RNA binding

GO cellular compartments

catalytic step 2 spliceosome, extracellular exosome, extracellular space, membrane, nuclear euchromatin, nucleoplasm, nucleus, ribonucleoprotein complex, supraspliceosomal complex

Note:
This protein is necessary for neural and muscle development in Xenopus and essential for brain development in zebrafish. It is required for resistance to DNA damage in human cell lines. May be linked with intellectual disability syndrome in humans.

Foamy virus Gag

Gag protein

(Q98826)

(Human foamy virus)

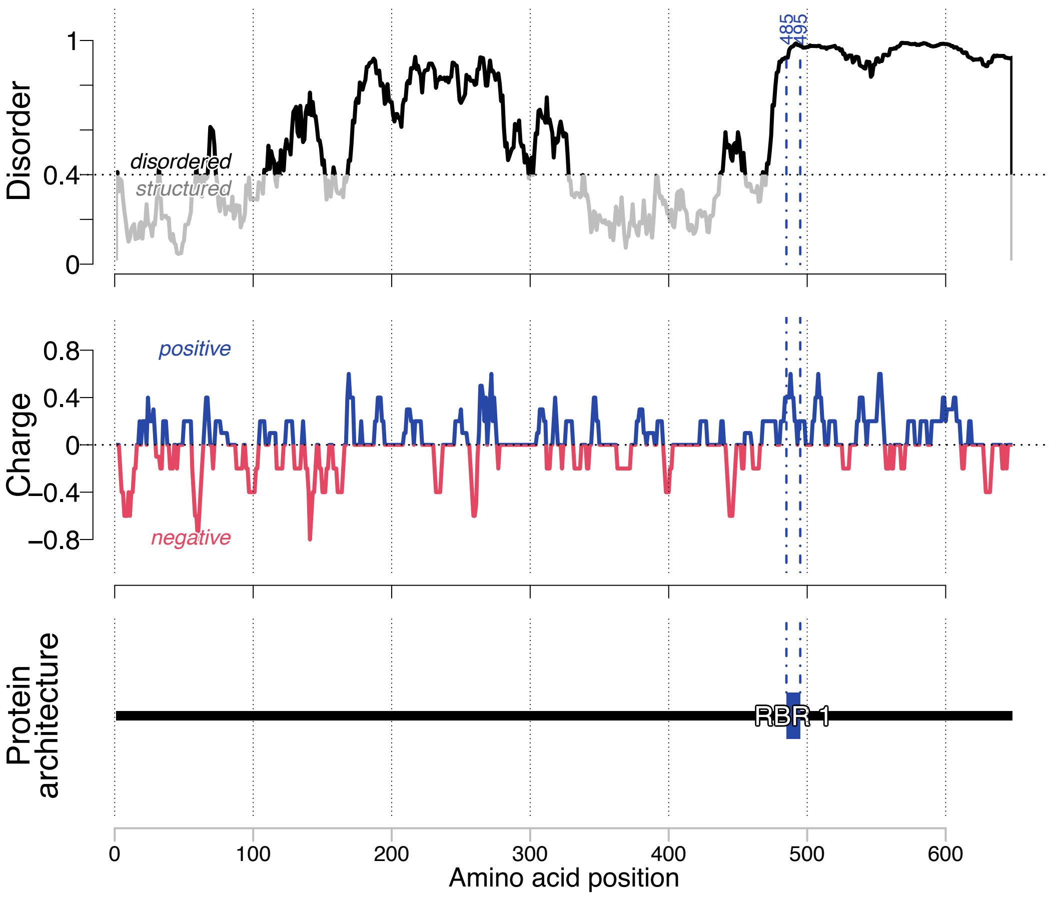

RNA BINDING DISORDERED PEPTIDE

RBR 1

485 RPSRGRGRGQN 495

GO biological processes

microtubule−dependent intracellular transport of viral material towards nucleus, viral entry into host cell, viral release from host cell

GO molecular functions

–

GO cellular compartments

host cell cytoplasm, host cell nucleus, host cytoskeleton, viral capsid

Note:
This protein harbours 4 additional RG regions but they do not contribute to RNA binding in vitro. All three GR regions are required for viral genomic RNA packaging.

TERF2

Telomeric repeat–binding factor 2

TTAGGG repeat–binding factor 2; Telomeric DNA–binding protein
(Q15554)

(Homo sapiens)

PROTEIN

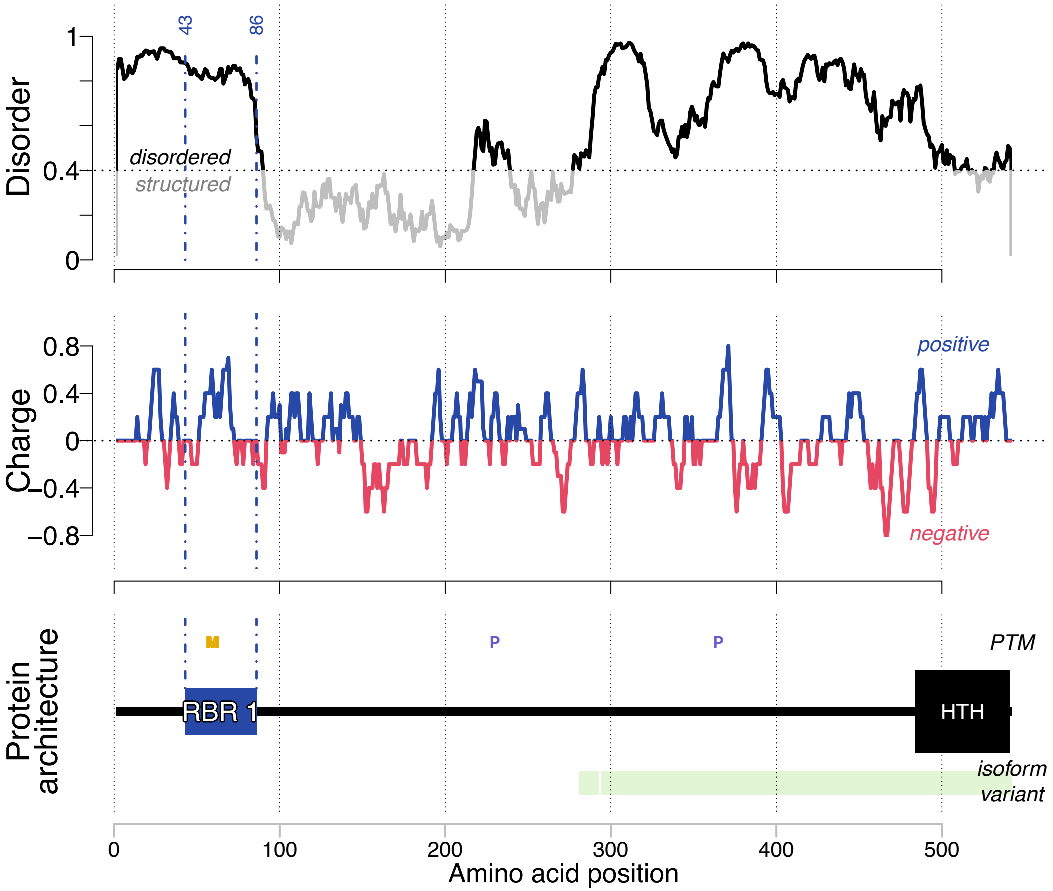

RNA BINDING DISORDERED PEPTIDE

RBR 1

43 MAGGGGSSDGSGRAAGRRASRSSGRARRGRHEPGLGGPAERGAG 86

GO biological processes

cell cycle, cellular senescence, in utero embryonic development, negative regulation of be ta–galactosidase activity, negative regulation of cell aging, negative regulation of cellu lar senescence, negative regulation of gene expression, negative regulation of t–circle fo rmation, negative regulation of telomere capping, negative regulation of telomere maintena...

GO molecular functions

double–stranded telomeric DNA binding, macromolecular complex binding, protein C–terminus binding, protein homodimerization activity, telomeric DNA binding

GO cellular compartments

chromosome, telomeric region, cytoplasm, Golgi apparatus, male germ cell nucleus, nuclear chromosome, telomeric region, nuclear telomere cap complex, nucleoplasm, nucleus, telosome

Note:
The isoform used in Zhang et al. (Nat Commun 2015; PMID: 26586091) is an N–terminally shortened (TRF2–S) version of the sequence displayed here. TRF2–S lacks the first 42 amino aci ds preceding the RNA–binding disordered region. As the short isoform is not annotated in UniProt at present, we display here the full protein.

XTUT7

TUT7/ZCCHC6

(R4IZH5)

(Xenopus laevis)

PROTEIN

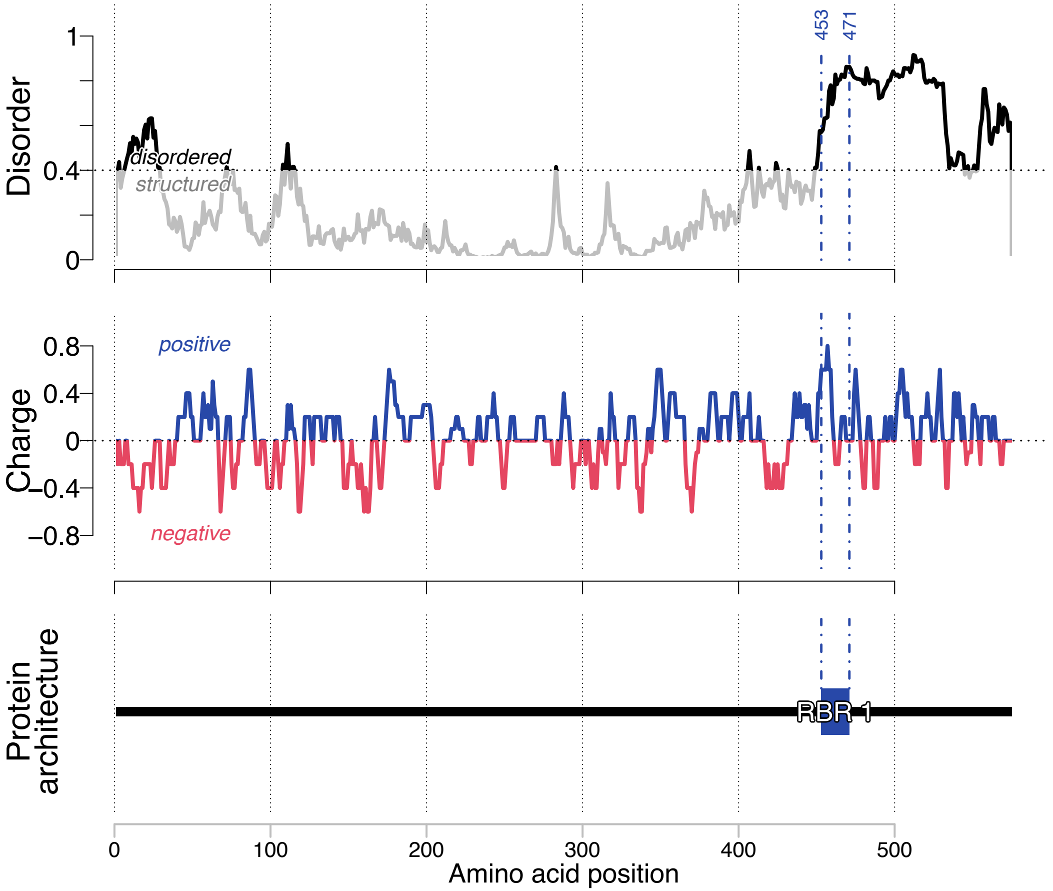

RNA BINDING DISORDERED PEPTIDE

RBR 1

453 MRRNRVRRRNNENAGNQRY 471

GO biological processes

–

GO molecular functions

–

GO cellular compartments

–

Tat

Protein Tat

Transactivating regulatory protein
(P04608)

(Human immunodeficiency virus type 1 group M subtype B (isolate HXB2))

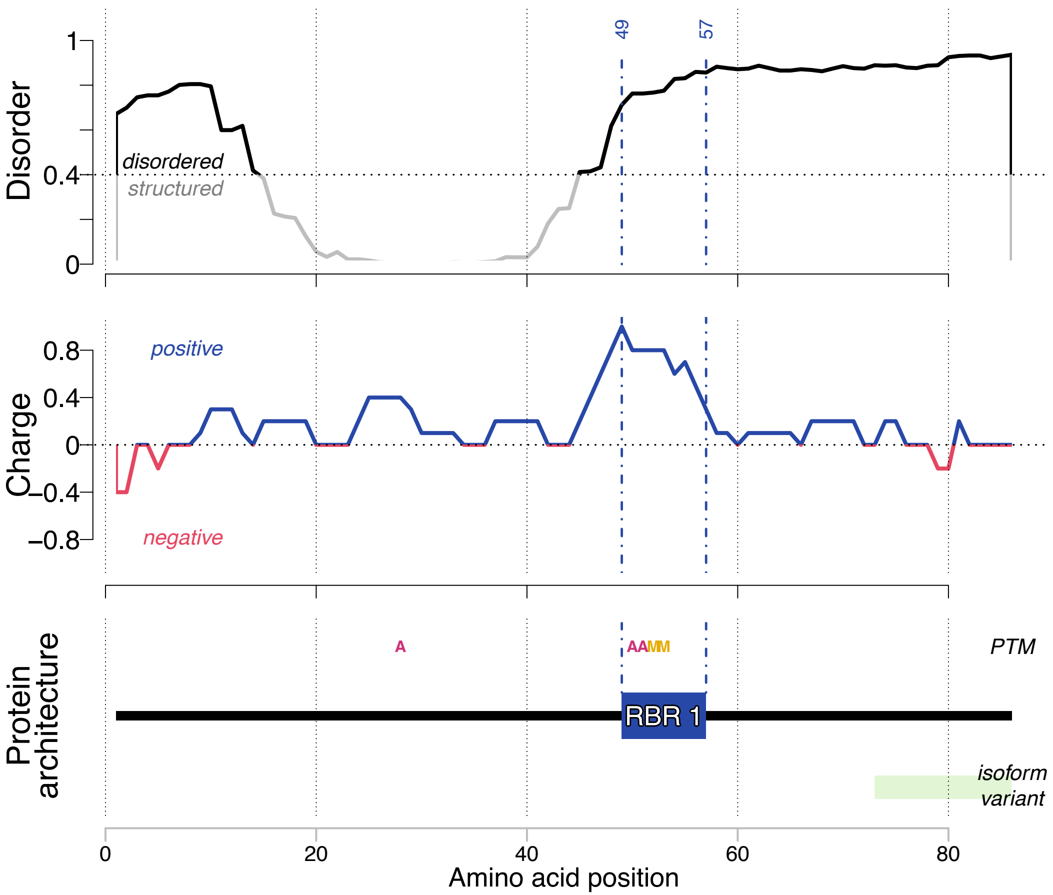

RNA BINDING DISORDERED PEPTIDE

RBR 1

49 **RKKRRQRRR** 57

GO biological processes

apoptotic process, modulation by virus of host chromatin organization, modulation by virus of host PP1 activity, positive regulation of viral transcription, suppression by virus of host type I interferon−mediated signaling pathway, transcription, DNA−templated, viral pr ocess

GO molecular functions

actinin binding, metal ion binding, RNA binding, sequence−specific DNA binding transcripti on factor activity

GO cellular compartments

extracellular region, host cell cytoplasm, host cell nucleolus, host cell nucleus

Note:
Remains flexible when bound to target RNA (TAR). The RNA−binding region overlaps with the nuclear localisation signal.

Rev

Protein Rev

ART/TRS; Anti−repression transactivator; Regulator of expression of viral proteins
(P69718)

(Human immunodeficiency virus type 1 group M subtype B (isolate HXB3))

PROTEIN

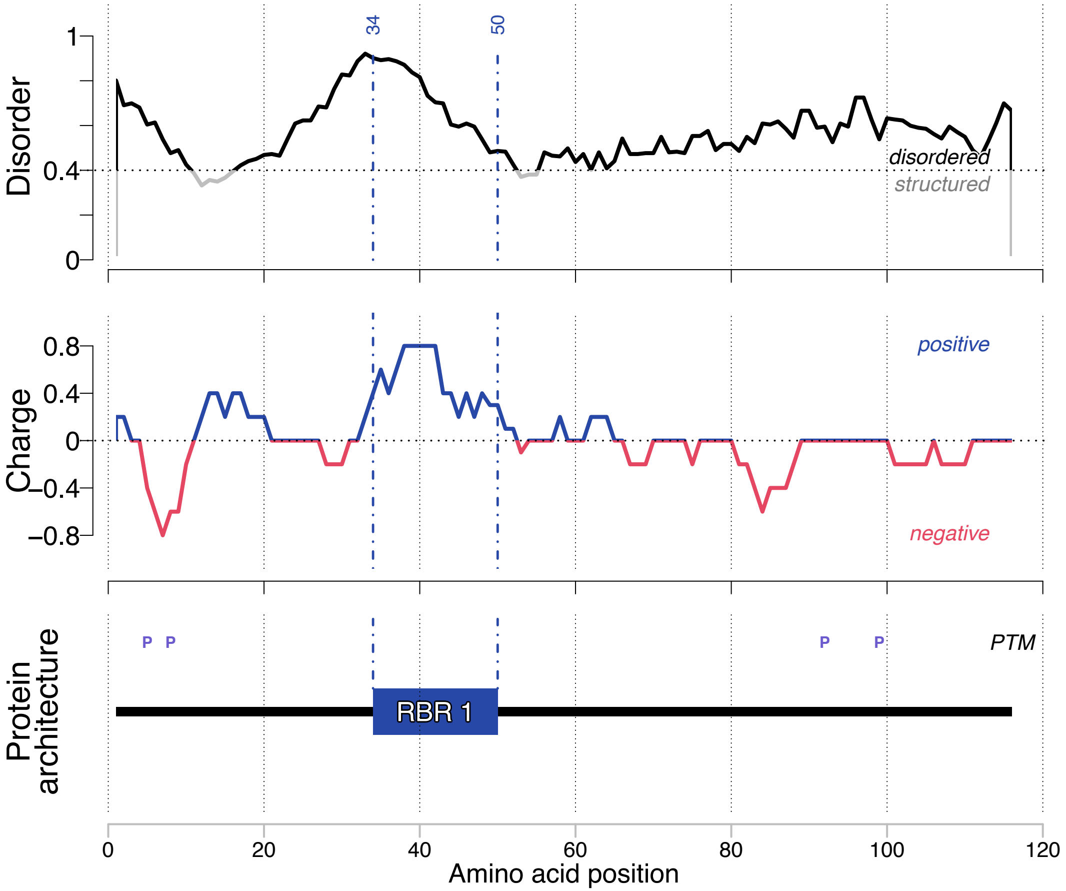

RNA BINDING DISORDERED PEPTIDE

RBR 1

34 TRQARRNRRRRWRERQR 50

GO biological processes

mRNA transport, viral process

GO molecular functions

RNA binding, sequence−specific DNA binding transcription factor activity

GO cellular compartments

host cell cytoplasm, host cell nucleolus

Note:
Forms an alpha−helical structured when RNA−bound, but retains some structural flexibility. Rev homomultimerises and forms a large Rev−RNA particle. Recent reports indicate also the target RNA (RRE) has structural flexibility. RNA−binding region overlaps with nuclear localisation signal.

Tat

Protein Tat

S ORF; Transactivating regulatory protein; bTat
(P19564)

(Bovine immunodeficiency virus (strain R29))

PROTEIN

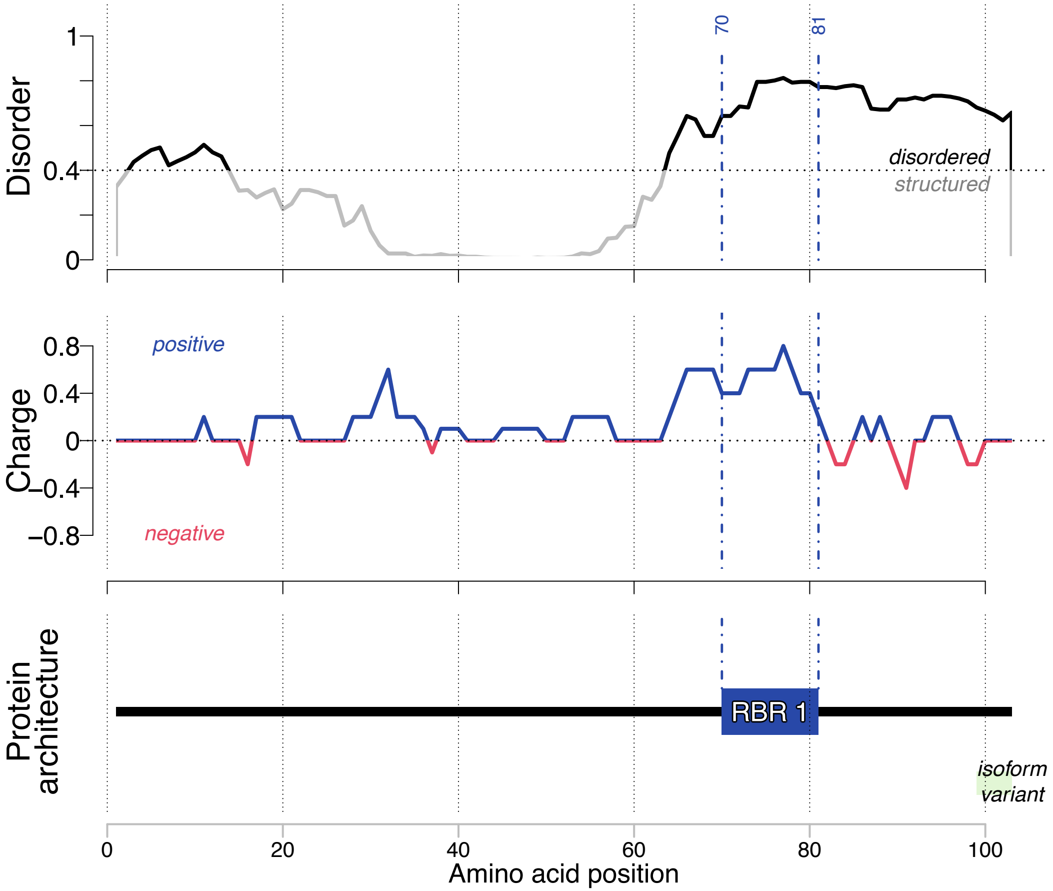

RNA BINDING DISORDERED PEPTIDE

RBR 1

70 RGTRGKGRRIRR 81

GO biological processes

transcription, DNA–templated, viral process

GO molecular functions

RNA binding, transcription factor activity, sequence–specific DNA binding

GO cellular compartments

host cell nucleolus

Coat protein

Capsid protein

Coat protein
(P03591)

(Alfalfa mosaic virus (strain 425 / isolate Leiden))

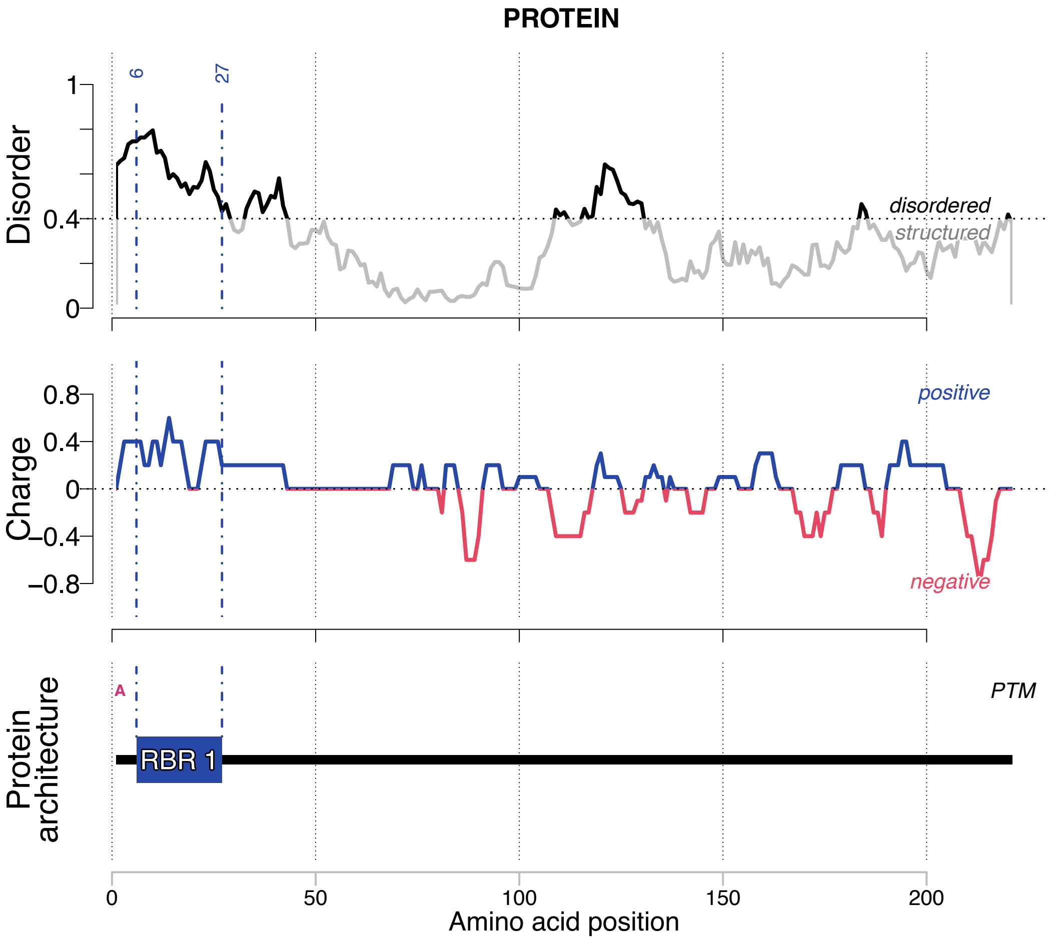

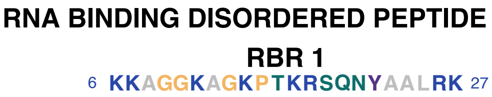

GO biological processes

translational initiation

GO molecular functions

RNA binding

GO cellular compartments

T=3 icosahedral viral capsid, viral nucleocapsid

Note:
This is a capsid protein in the plant infecting alfalfa mosaic virus.

PAPD5

Non–canonical poly(A) RNA polymerase PAPD5

PAP–associated domain–containing protein 5; Terminal uridylyltransferase 3; Topoisomerase–related function protein 4–2
(Q8NDF8)

(Homo sapiens)

PROTEIN

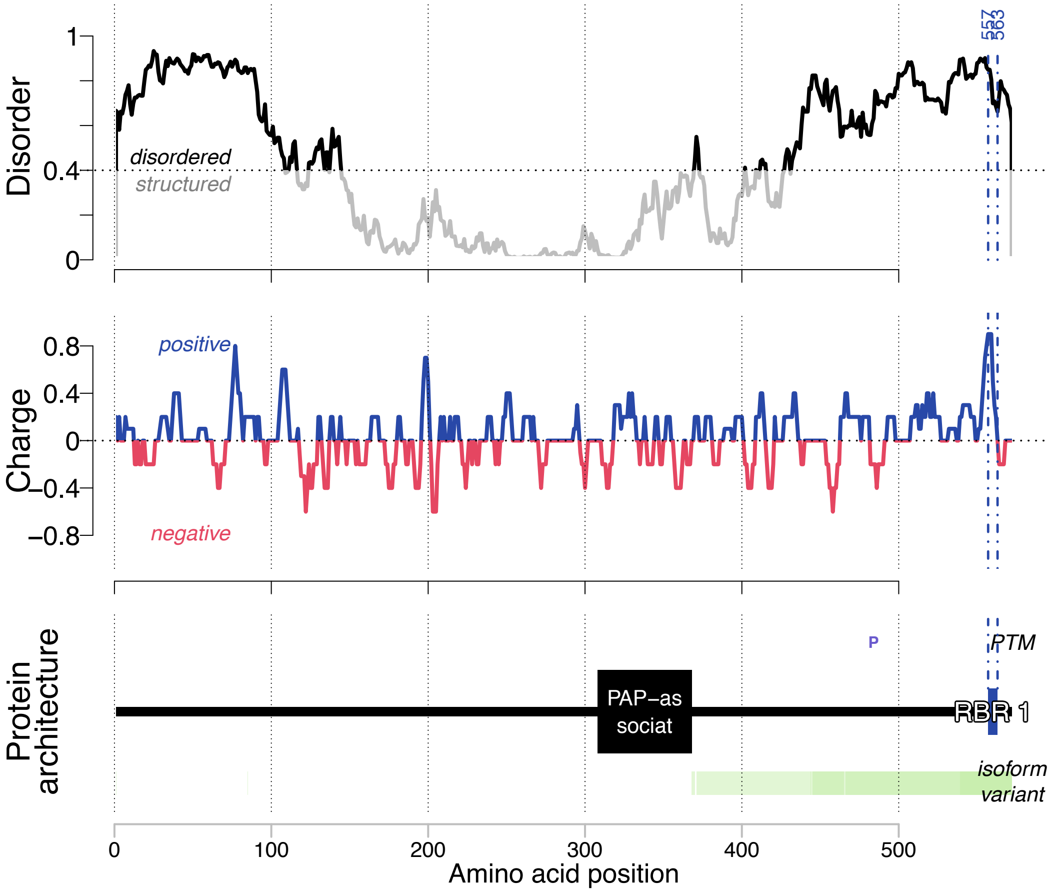

RNA BINDING DISORDERED PEPTIDE

RBR 1

557 **KKRKHKR** 563

GO biological processes

cell division, histone mRNA catabolic process, mitotic nuclear division, mRNA processing, rRNA processing

GO molecular functions

DNA binding, DNA–directed DNA polymerase activity, metal ion binding, poly(A) RNA binding, polynucleotide adenylyltransferase activity

GO cellular compartments

cytoplasm, nucleolus

SDAD1

Protein SDA1 homolog

Nucleolar protein 130; SDA1 domain–containing protein 1
(Q9NVU7)

(Homo sapiens)

PROTEIN

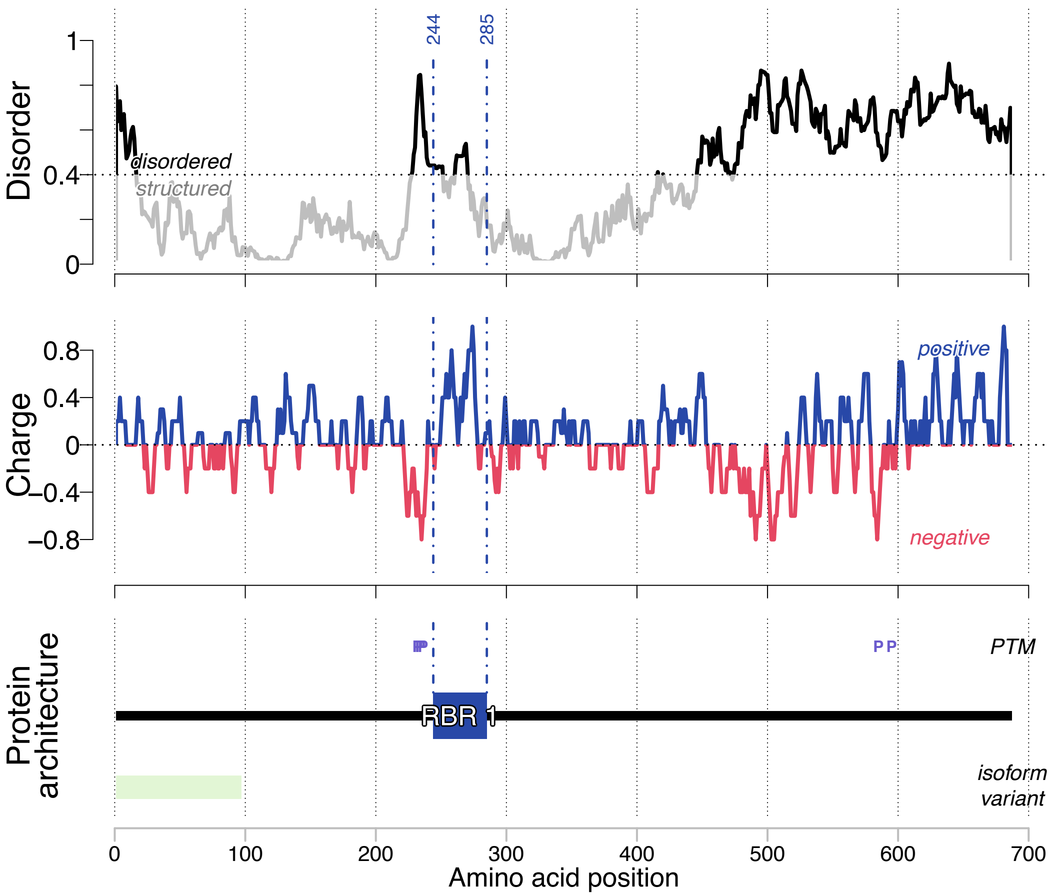

RNA BINDING DISORDERED PEPTIDE

RBR 1

244 RDLLVQYATGKKSSKNKKKLEKAMKVLKKQKKKKKPEVFNFS 285

GO biological processes

actin cytoskeleton organization, protein transport, ribosomal large subunit biogenesis, ri
bosomal large subunit export from nucleus

GO molecular functions

–

GO cellular compartments

nucleolus, nucleus

HMGA1

High mobility group protein HMG−I/HMG−Y

High mobility group AT−hook protein 1; High mobility group protein R
(P17096)

(Homo sapiens)

PROTEIN

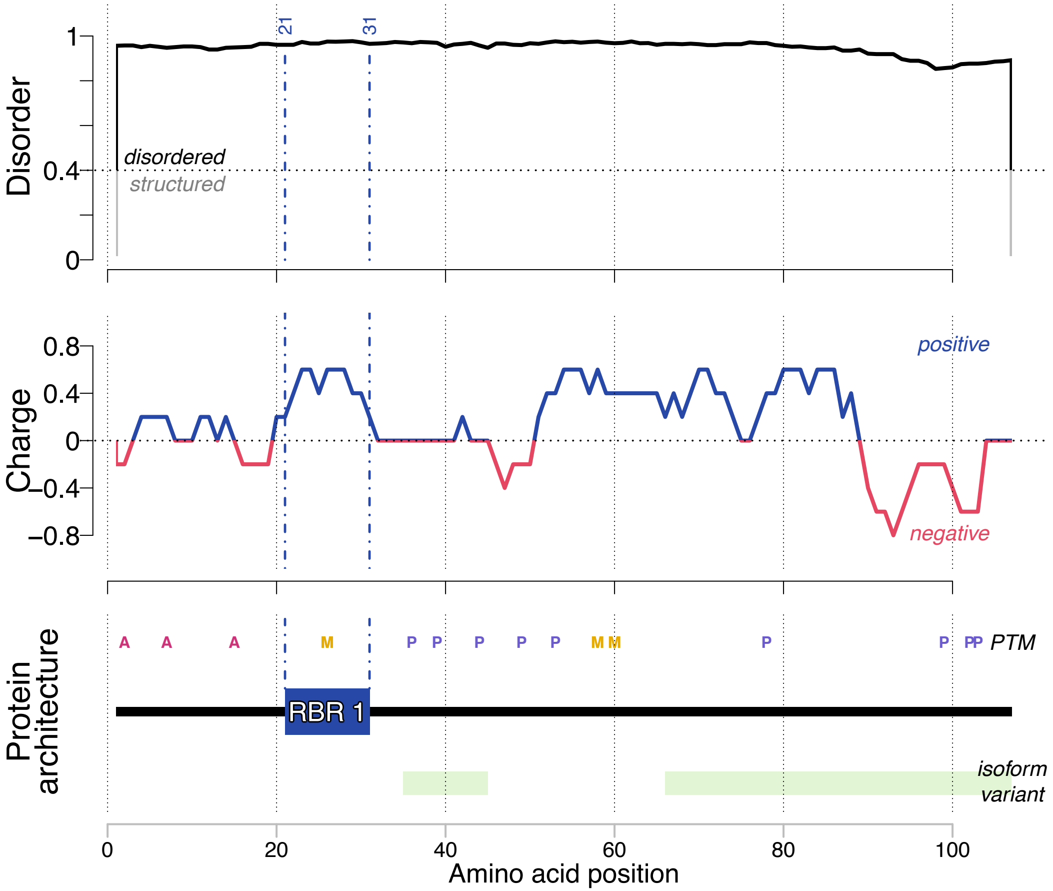

RNA BINDING DISORDERED PEPTIDE

RBR 1

21 TEKRGRGRPRK 31

GO biological processes

base−excision repair, DNA unwinding involved in DNA replication, establishment of integrat
ed proviral latency, negative regulation of cell proliferation, negative regulation of chr
omatin silencing, negative regulation of transcription, DNA−templated, nucleosome disassem
bly, oncogene−induced cell senescence, positive regulation of cellular senescence, positiv...

GO molecular functions

5'−deoxyribose−5−phosphate lyase activity, AT DNA binding, chromatin binding, DNA binding,
DNA−(apurinic or apyrimidinic site) lyase activity, enzyme binding, ligand−dependent nucl
ear receptor transcription coactivator activity, peroxisome proliferator activated recepto
r binding, retinoic acid receptor binding, retinoid X receptor binding, RNA polymerase II ...

GO cellular compartments

cytosol, focal adhesion, nucleoplasm, nucleus, RNA polymerase II transcription factor comp
lex, senescence−associated heterochromatin focus, transcription factor complex

Note:
This protein has three (e)AT−hooks, the first one is shown to be RNA binding. Region becomes structured when bound to DNA.

Tip5

Bromodomain adjacent to zinc finger domain protein 2A

Transcription termination factor I–interacting protein 5; hWALp3

(Q9UIF9)

(Homo sapiens)

PROTEIN

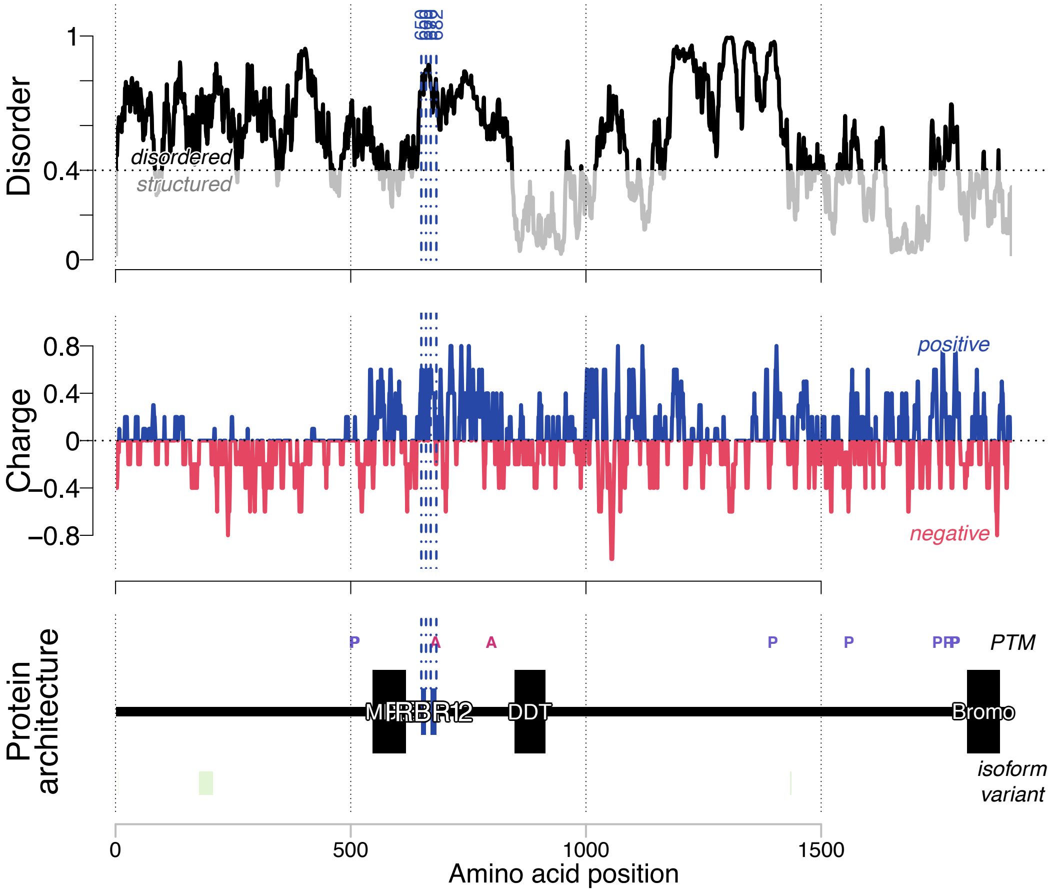

RNA BINDING DISORDERED PEPTIDE

RBR 1

650 GKRGRPRNTEK 660

RBR 2

670 KRGRGRPPKVKIT 682

GO biological processes

chromatin remodeling, chromatin silencing at rDNA, DNA methylation, gene expression, heterochromatin assembly involved in chromatin silencing, histone deacetylation, histone H3–K9 methylation, histone H4 deacetylation, histone H4–K20 methylation, negative regulation of gene expression, epigenetic, regulation of gene expression, epigenetic, regulation of tran...

GO molecular functions

DNA binding, ligand–dependent nuclear receptor binding, lysine–acetylated histone binding, RNA binding, zinc ion binding

GO cellular compartments

chromatin silencing complex, nucleolus, rDNA heterochromatin

Note:
Tip5 is involved in epigenetic alterations in prostate cancer and its overexpression predicts disease recurrence.

PTOV1

Prostate tumor–overexpressed gene 1 protein

Activator interaction domain–containing protein 2
(Q86YD1)

(Homo sapiens)

PROTEIN

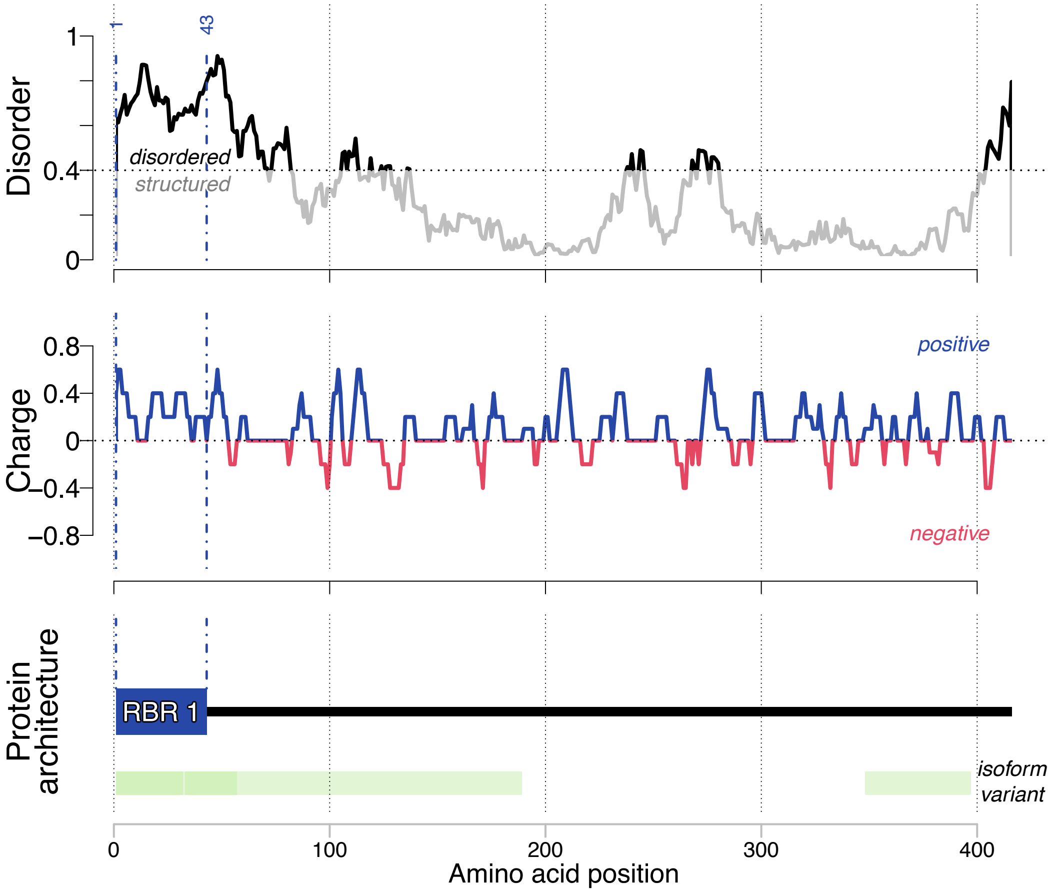

RNA BINDING DISORDERED PEPTIDE

RBR 1

1 MVRPRRAPYRSGAGGPLGGRGRPPRPLVVRAVRSRSWPASPRG 43

GO biological processes

regulation of transcription, DNA–templated, transcription, DNA–templated

GO molecular functions

–

GO cellular compartments

intracellular membrane–bounded organelle, nucleus, perinuclear region of cytoplasm, plasma membrane

Note:
PTOV1 promotes cell proliferation and has been linked to malignant tumours.

GPBP1

Vasculin

GC–rich promoter–binding protein 1; Vascular wall–linked protein
(Q86WP2)

(Homo sapiens)

PROTEIN

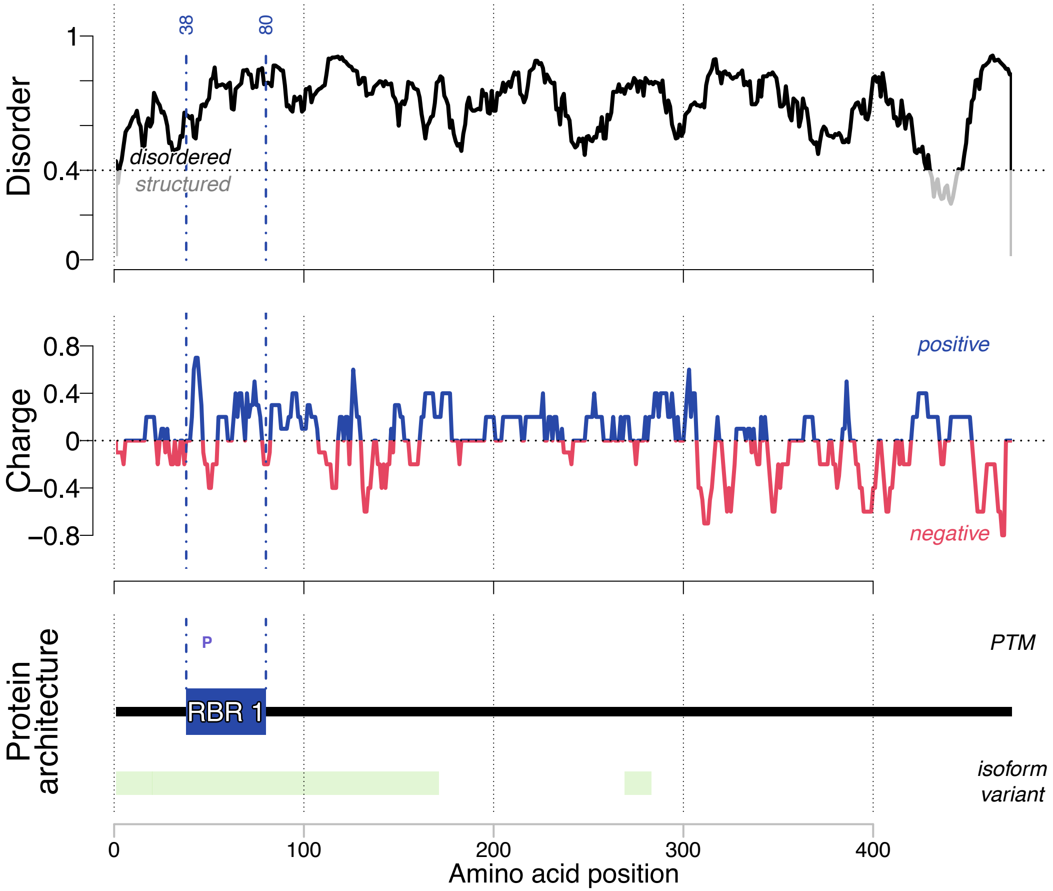

RNA BINDING DISORDERED PEPTIDE

RBR 1

38 NRYDVNRRRHNSSDGFDSAIGRPNGGNFGRKEKNGWRTHGRNG 80

GO biological processes

positive regulation of transcription, DNA–templated, transcription, DNA–templated

GO molecular functions

DNA binding, sequence–specific DNA binding transcription factor activity

GO cellular compartments

cytoplasm, intracellular membrane–bounded organelle, nucleolus, plasma membrane

SRSF2

Serine/arginine–rich splicing factor 2

Protein PR264; Splicing component, 35 kDa; Splicing factor SC35; Splicing factor, arginine/serine–rich 2
(Q01130)

(Homo sapiens)

PROTEIN

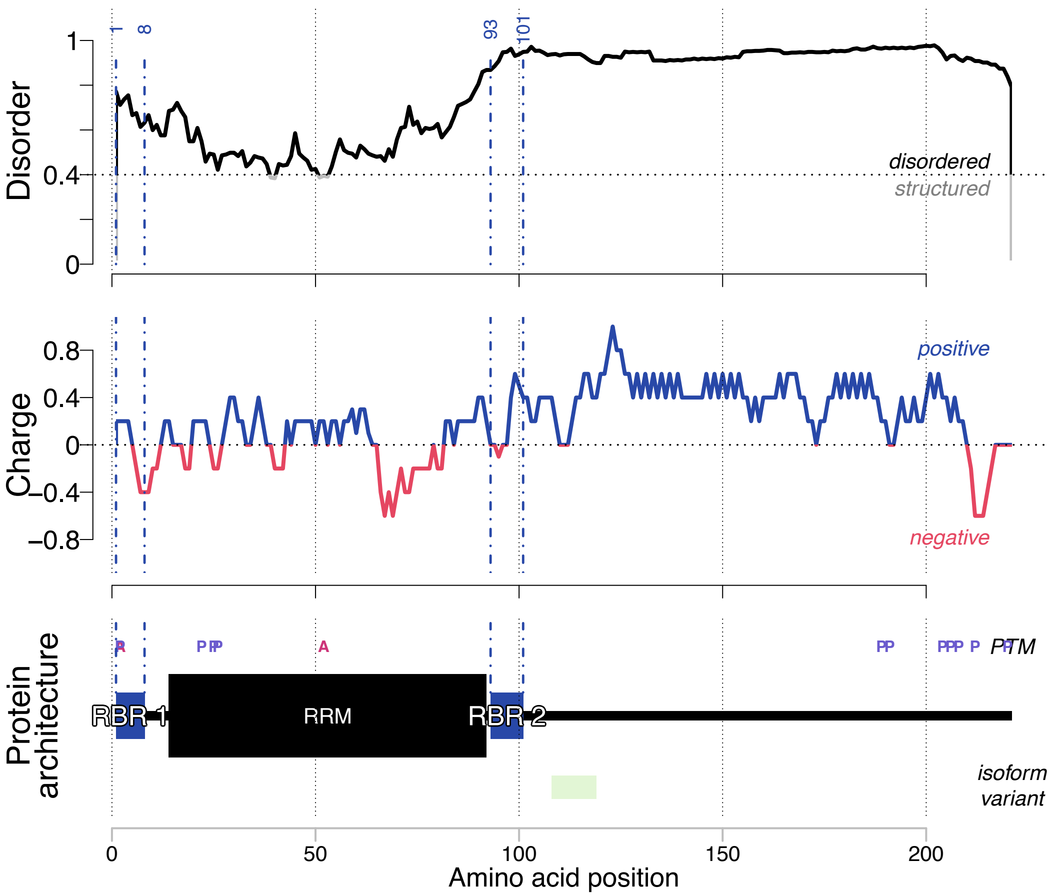

RNA BINDING DISORDERED PEPTIDE

RBR 1
1 MSYGRPPP 8

RBR 2
93 GRPPDSHHS 101

GO biological processes

gene expression, mRNA 3'–end processing, mRNA export from nucleus, mRNA processing, mRNA splicing, via spliceosome, negative regulation of nucleic acid–templated transcription, regulation of alternative mRNA splicing, via spliceosome, RNA splicing, termination of RNA polymerase II transcription, transcription from RNA polymerase II promoter

GO molecular functions

nucleotide binding, poly(A) RNA binding, pre–mRNA binding, transcription corepressor activity

GO cellular compartments

cytoplasm, extracellular exosome, nuclear speck, nucleoplasm, nucleus, spliceosomal complex

Note:
These two disordered GRP regions flank the RRM and become more structured upon RNA binding. Pro6 and especially Pro95 form extensive contacts with the RNA through stacking. Deletion of these regions results in a slight decrease in affinity. Mutations in disordered RNA binding region (which mainly occur at P95) are associated with myelodysplastic syndromes and chronic myelomonocytic leukemia. P95H alters RNA binding affinity and results in altered splicing.

Tra2B1

Transformer–2 protein homolog beta

Splicing factor, arginine/serine–rich 10; Transformer–2 protein homolog B
(P62995)

(Homo sapiens)

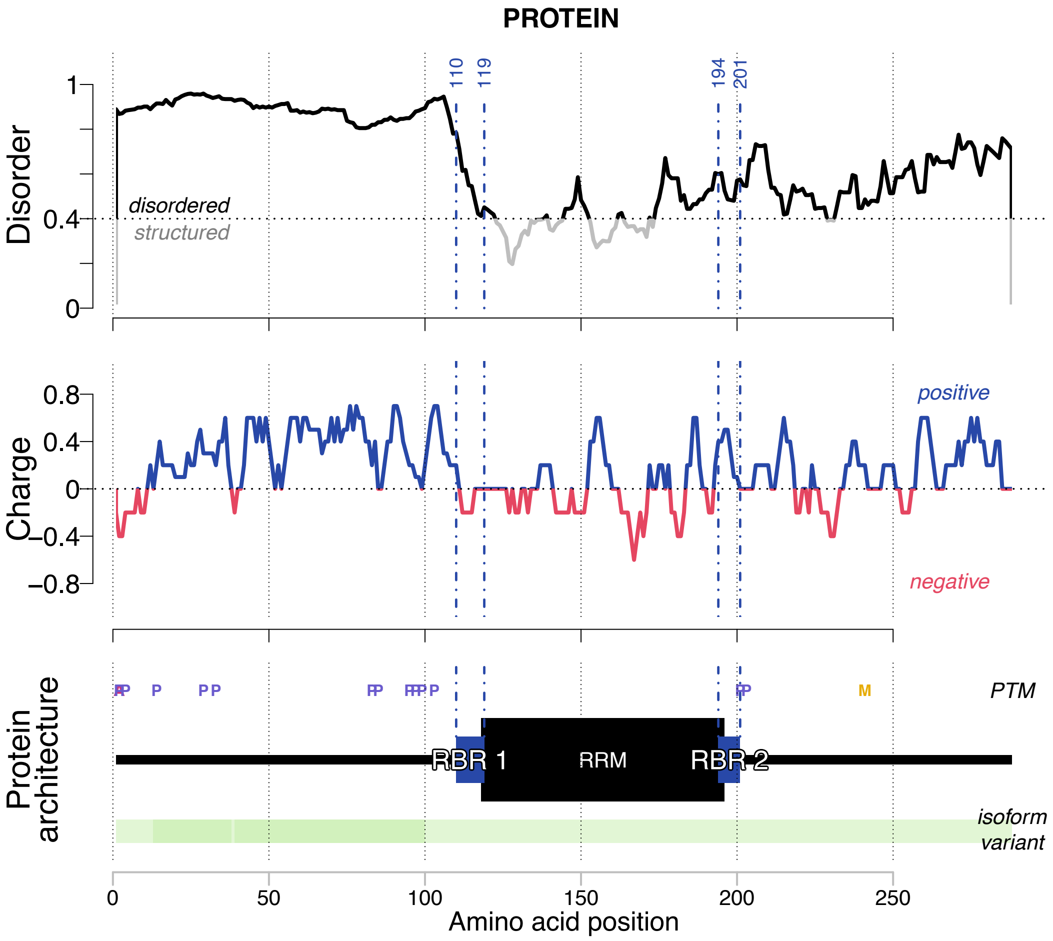

RNA BINDING DISORDERED PEPTIDE

RBR 1

110 NRANPDPNCC 119

RBR 2

194 SITKRPHT 201

GO biological processes

cerebral cortex regionalization, mRNA splicing, via spliceosome, positive regulation of mR NA splicing, via spliceosome, regulation of alternative mRNA splicing, via spliceosome, RN A splicing, via transesterification reactions

GO molecular functions

mRNA binding, nucleotide binding, poly(A) RNA binding

GO cellular compartments

nucleus

Note:

These two disordered regions flank the RRM and contribute to formation of a binding pocket. The regions become structured upon RNA binding. The Args on each flanking region are spe cifically involved in the interaction. Deletion of flanking regions (individually) decreases binding affinity. RNA binding induces positioning of the two RS regions in this protein relative to each other.

hnRNPA1

Heterogeneous nuclear ribonucleoprotein A1

Helix–destabilizing protein; Single–strand RNA–binding protein; hnRNP core protein A1
(P09651)

(Homo sapiens)

PROTEIN

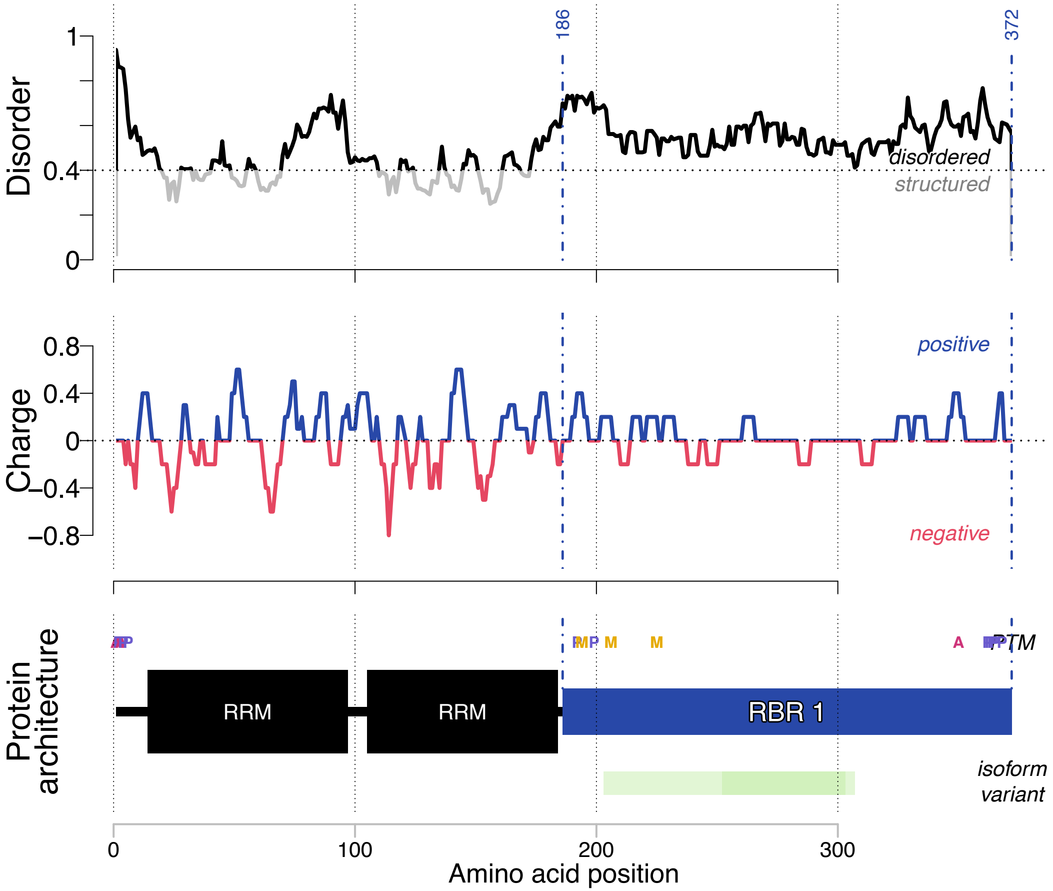

RNA BINDING DISORDERED PEPTIDE

RBR 1

186 MASASSSQRGRSGSGNFGGGRGGGFGGNDNFGRGGNFSGRGGFGGSRGGGGYGGSGDGYN
GFGNDGGYGGGGPGYSGGSRGYGSGGQGYGNQGSGYGGSGSYDSYNNGGGGGFGGGSGSN
FGGGGSYNDFGNYNNQSSNFGPMKGGNFGGRSSGPYGGGGQYFAKPRNQGGYGGSSSSSS
YGSGRRF 372

GO biological processes

alternative mRNA splicing, via spliceosome, gene expression, mRNA processing, mRNA splicin
g, via spliceosome, mRNA transport, nuclear export, nuclear import, RNA export from nucleu
s, RNA splicing, viral process

GO molecular functions

nucleotide binding, poly(A) RNA binding, RNA binding, single–stranded DNA binding, single–
stranded RNA binding

GO cellular compartments

catalytic step 2 spliceosome, cytoplasm, extracellular exosome, intermediate filament cyto
skeleton, membrane, nucleoplasm, ribonucleoprotein complex, spliceosomal complex

Note:

The low complexity region of hnRNPA1 promotes phase separation and granule assembly. The low complexity sequence contains an RGG region that has been proposed to bind RNA. Addition
al regions may also contribute to RNA binding. Note also the presence of FG–dipeptides in this sequence (similar to Nott et al.) These gene has been linked to amyotrophic lateral s
clerosis, frontotemporal dementia, myopathy, and multisystem proteinopathy.

LUZP4

Leucine zipper protein 4

Cancer/testis antigen 28; Tumor antigen HOM–TES–85

(Q9P127)

(Homo sapiens)

PROTEIN

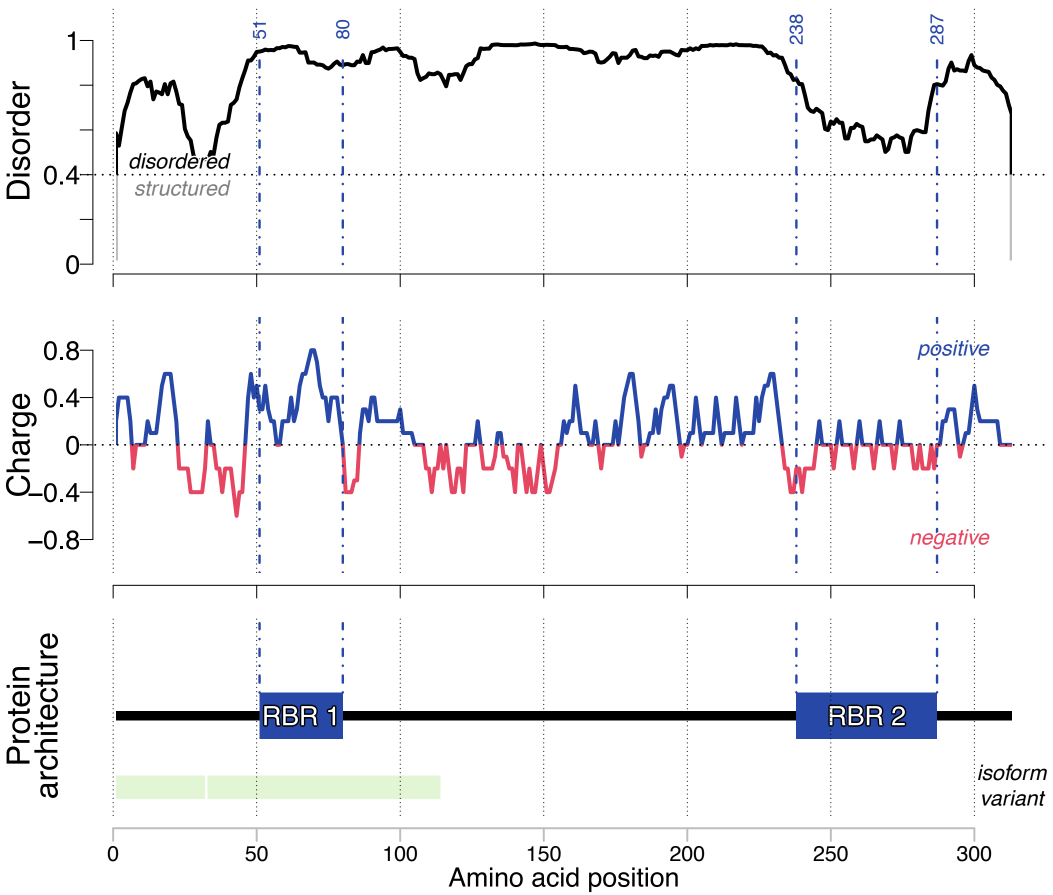

RNA BINDING DISORDERED PEPTIDE

RBR 1

51 RQNHSKKESPSRQQSKAHRHRHRRGYSRCR 80

RBR 2

238 LVDTQSDLIATQRDLIATQKDLIATQRDLIATQRDLIVTQRDLVATERDL 287

GO biological processes

cell growth, mRNA export from nucleus

GO molecular functions

poly(A) RNA binding, single–stranded DNA binding, single–stranded RNA binding

GO cellular compartments

cytoplasm, nucleus

Note:

The entire protein is predicted disordered. The precise boundaries of the two RNA–binding regions are not sharply defined. The second RNA–binding regions overlaps a predicred leuci ne zipper motif that may orient positively charged residues within this region.

ORF57

mRNA export factor ICP27 homolog

52 kDa immediate−early phosphoprotein; EB2 protein homolog
(P13199)

(Saimiriine herpesvirus 2 (strain 11))

PROTEIN

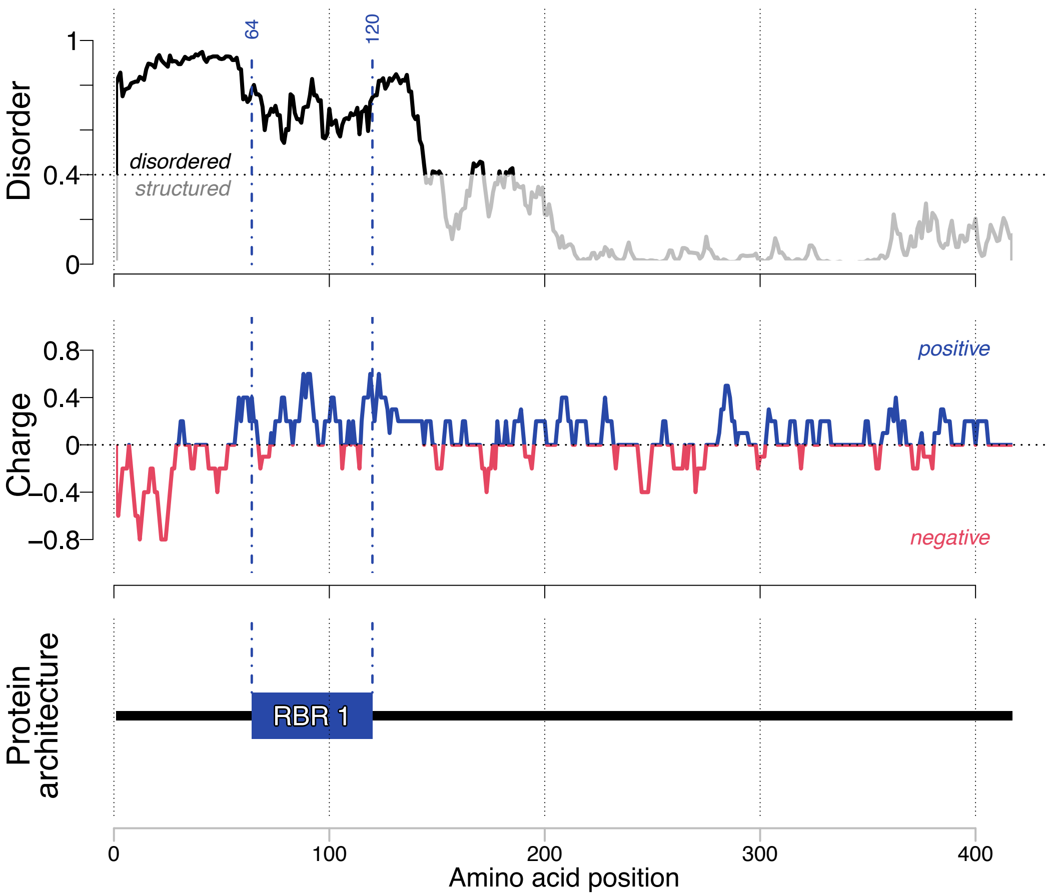

RNA BINDING DISORDERED PEPTIDE

RBR 1

64 RQRSPITWEHQSPLSRVYRSPSPMRFGKRPRISSNSTSRSCKTSWADRVREAAAQRR 120

GO biological processes

regulation of transcription, DNA−templated, transcription, DNA−templated

GO molecular functions

metal ion binding, RNA binding

GO cellular compartments

host cell cytoplasm, host cell nucleus

Note:
The last 15 amino acids of the highlighted sequece form an alpha−helical structure. A ternary complex may form between ORF57, ALYREF, and viral RNA.

APC

Adenomatous polyposis coli protein

(Q61315)

(Mus musculus)

PROTEIN

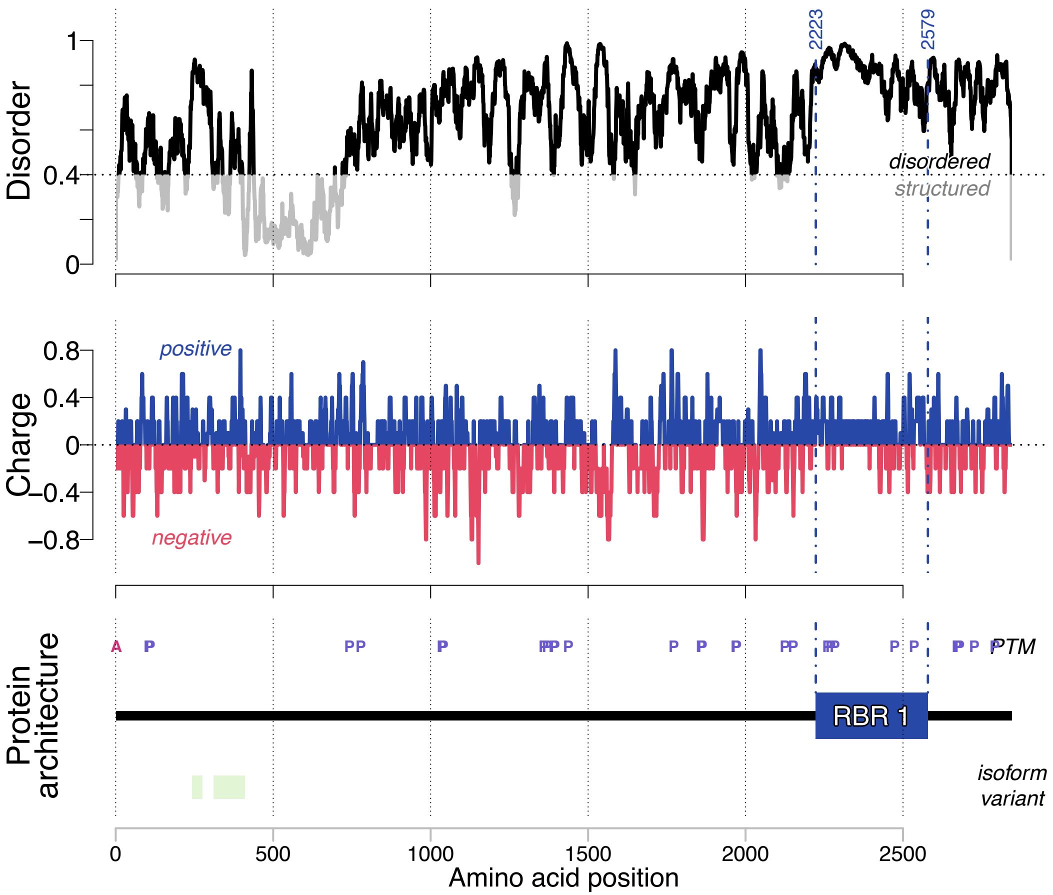

RNA BINDING DISORDERED PEPTIDE

RBR 1

2223SISRGRTMIHIPGLRNSSSSTSPVSKKGPPLKTPASKSPSEGPGATTSPRGTKPAGKSEL
SPITRQTSQISGSNKGSSRSGSRDSTPSRPTQQPLSRPMQSPGRNSISPGRNGISPPNKL
SQLPRTSSPSTASTKSSGSGKMSYTSPGRQLSQQNLTKQASLSKNASSIPRSESASKGLN
QMSNGNGSNKKVELSRMSSTKSSGSESDSSERPALVRQSTFIKEAPSPTLRRKLEESASF
ESLSPSSRPDSPTRSQAQTPVLSPSLPDMSLSTHPSVQAGGWRKLPPNLSPTIEYNDGRP
TKRHDIARSHSESPSRLPINRAGTWKREHSKHSSSLPRVSTWRRTGSSSSILSASSE2579

GO biological processes

anterior/posterior pattern specification, axis specification, axonogenesis, canonical Wnt signaling pathway, canonical Wnt signaling pathway involved in negative regulation of apop totic process, canonical Wnt signaling pathway involved in positive regulation of apoptoti c process, cell cycle arrest, cell migration, cellular response to DNA damage stimulus, ch...

GO molecular functions

beta−catenin binding, gamma−catenin binding, microtubule binding, microtubule plus−end bin ding, protease binding, protein kinase binding, protein kinase regulator activity

GO cellular compartments

adherens junction, axon, axonal growth cone, beta−catenin destruction complex, bicellular tight junction, catenin complex, cell projection, cell projection membrane, centrosome, cy toplasm, growth cone, kinetochore, lamellipodium, lateral plasma membrane, microtubule, nu cleus, plasma membrane, ruffle membrane, Scrib−APC−beta−catenin complex

Note:
Linked to hereditary desmoid disease, medulloblastoma, and Mismatch repair cancer syndrome.

CTCF

Transcriptional repressor CTCF

11–zinc finger protein; CCCTC–binding factor; CTCFL paralog
(P49711)

(Homo sapiens)

PROTEIN

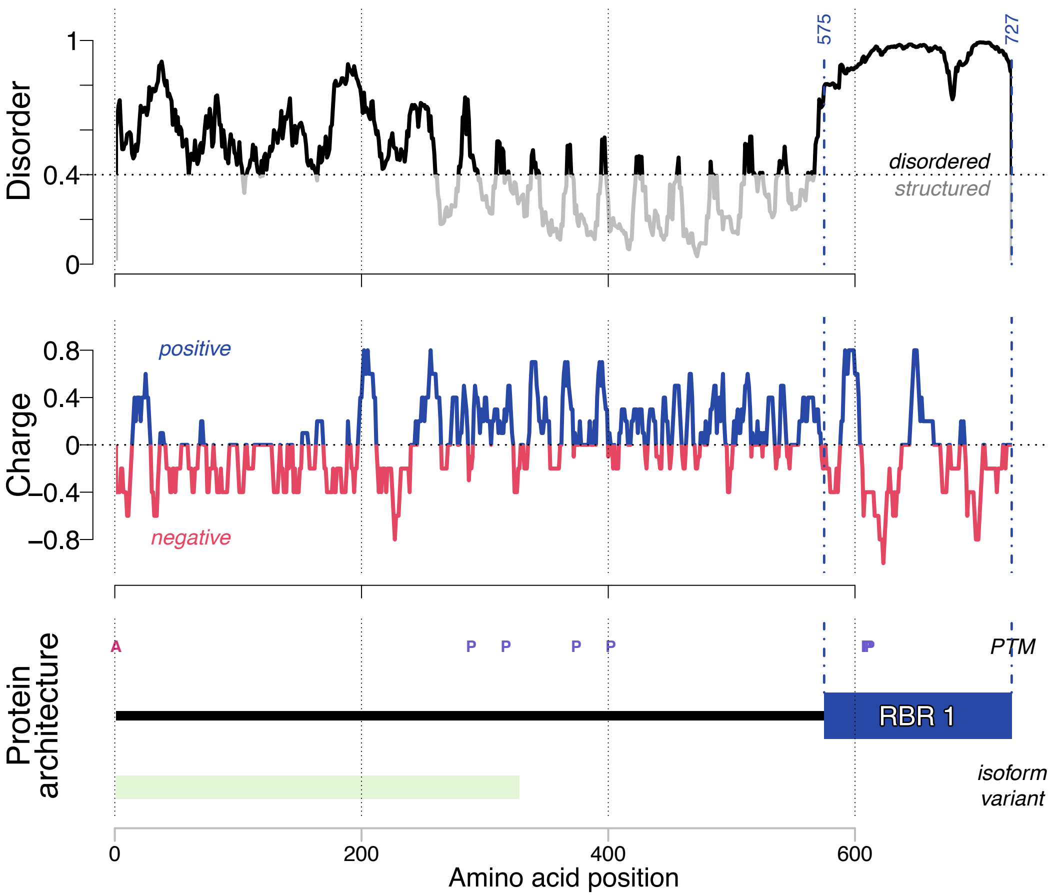

RNA BINDING DISORDERED PEPTIDE

RBR 1

575 DNCAGPDGVEGENGGETKKSKRGRKRKMRSKKEDSSDSENAEPDLDDNEDEEEPAVEIEP
EPEPQPVTPAPPPAKKRRGRPPGRTNQPKQNQPTAIIQVEDQNTGAIENIIVEVKKEPDA
EPAEGEEEEAQPAATDAPNGDLTPEMILSMMDR 727

GO biological processes

chromatin modification, chromosome segregation, DNA methylation, maintenance of DNA methyl
ation, negative regulation of transcription from RNA polymerase II promoter, negative regu
lation of transcription, DNA–templated, nucleosome positioning, positive regulation of gen
e expression, positive regulation of transcription, DNA–templated, regulation of centromer...

GO molecular functions

chromatin insulator sequence binding, RNA polymerase II core promoter proximal region sequ
ence–specific DNA binding, RNA polymerase II core promoter proximal region sequence–specif
ic DNA binding transcription factor activity involved in negative regulation of transcript
ion, RNA polymerase II transcription regulatory region sequence–specific DNA binding trans...

GO cellular compartments

chromosome, centromeric region, condensed chromosome, nucleolus, nucleoplasm, nucleus

Note:
Deletion of the basic patch containing region (NCAGPDGVEGENGGETKKSKRGRKRKMRSKKEDSSDSEN) drastically reduced RNA binding. CTCF has 11 annotated zinc finger domains between residues 266–577 not visualised here as they are not currently annotated in UniProt under domain feature key.

Df31

Anon1A4; CG2207–PA, isoform A; Decondensation factor 31, isoform B; Decondensation factor 31, isoform F; LD21289p

(O16043)

(Drosophila melanogaster)

PROTEIN

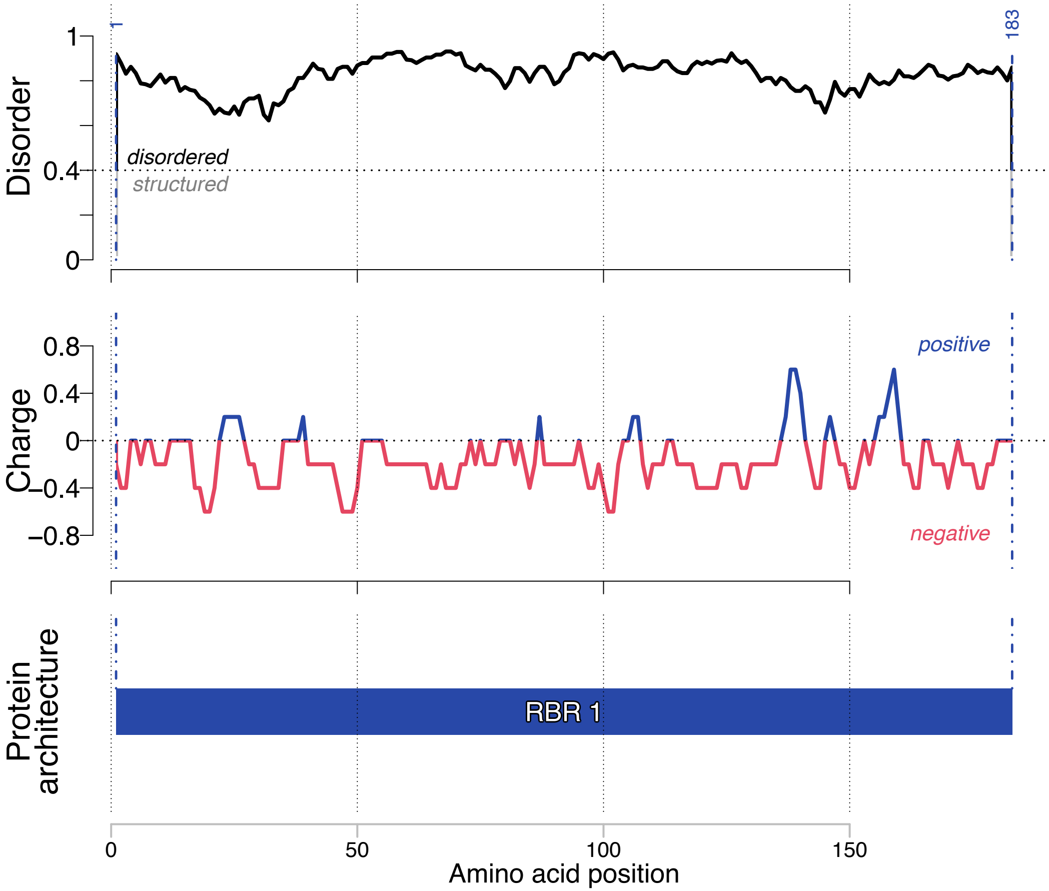

RNA BINDING DISORDERED PEPTIDE

RBR 1

1 MADVAEQKNETPVVEKVAAEEVDAVKKDAVAAEEVAAEKASITENGGAEEESVAKENGAA
DSSATEPTDAVDGEKASEPTVSFAADKDEKKDEDKKEDSAADGEDTKKESSEAVLPAVEN
GSEEVTNGDSTDAPAIEAVKRKVDEAAAKADEAVATPEKKAKLDEASTKDEVQNGAEASE
VAA 183

GO biological processes

chromatin organization, nucleosome assembly

GO molecular functions

histone binding

GO cellular compartments

chromatin, cytoplasm, nucleus, plasma membrane

Note:
This short protein is entirely disordered. The sequence provided is the full length protein sequence as region involved in RNA binding was not narrowed down in the study. Df31 bind s histones in a co–operative manner with RNA binding. Whether the binding sites overlap is not known.

Ezh2

Histone–lysine N–methyltransferase EZH2

ENX–1; Enhancer of zeste homolog 2
(Q61188)

(Mus musculus)

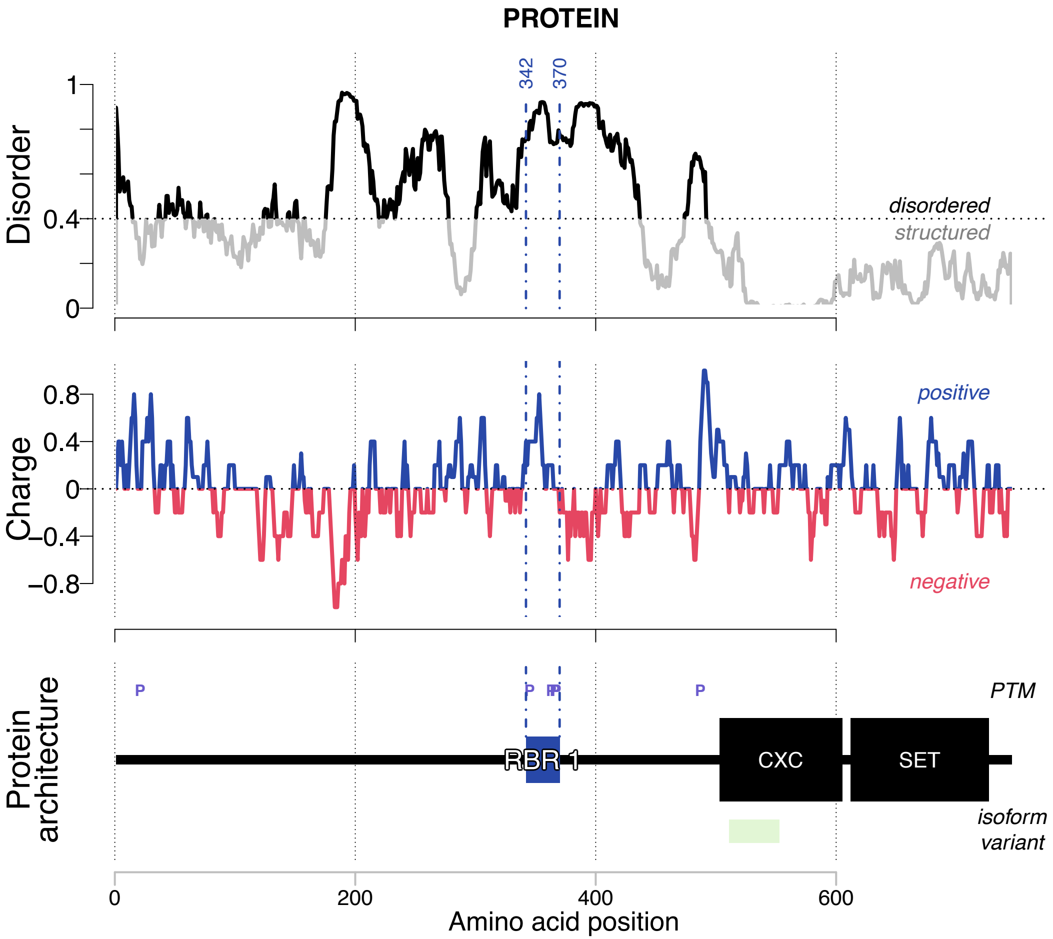

RNA BINDING DISORDERED PEPTIDE

RBR 1

342 R I KTPPKRPGGRRRGRLPNNSSRPSTPT I 370

GO biological processes

cellular response to hydrogen peroxide, cerebellar cortex development, DNA methylation, G1 to G0 transition, hippocampus development, histone H3–K27 methylation, histone methylation, negative regulation of epidermal cell differentiation, negative regulation of G1/S transition of mitotic cell cycle, negative regulation of gene expression, epigenetic, negative...

GO molecular functions

chromatin binding, chromatin DNA binding, core promoter binding, histone methyltransferase activity, histone–lysine N–methyltransferase activity, protein–lysine N–methyltransferase activity, RNA binding, sequence–specific DNA binding

GO cellular compartments

cytoplasm, ESC/E(Z) complex, nuclear chromatin, nucleoplasm, nucleus, pronucleus

Note:
Deletion is early embryonic lethal.

Nrep

Neuronal regeneration–related protein
Neuronal protein 3.1; Protein p311
(Q07475)

(Mus musculus)

PROTEIN

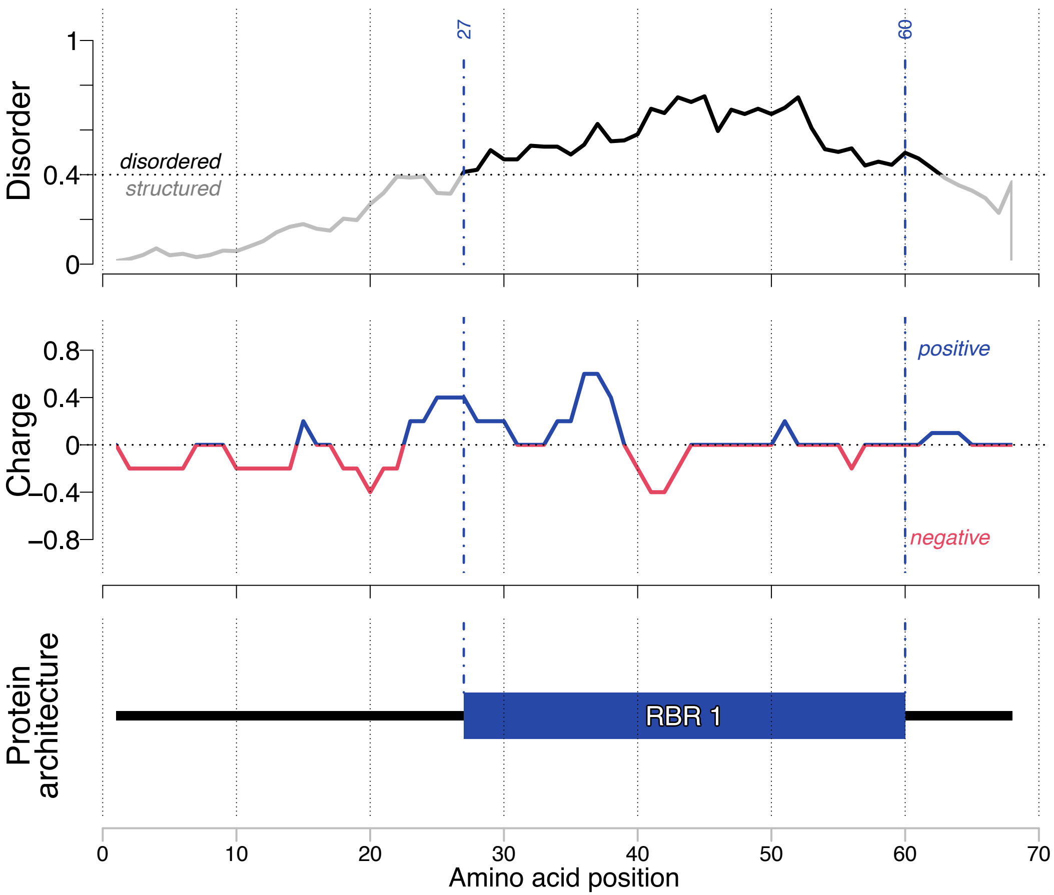

RNA BINDING DISORDERED PEPTIDE

RBR 1

27 KGRLPVPKEVNRKKMEETGAASLTPPGSREFTSP 60

GO biological processes

axon regeneration, regulation of neuron differentiation, regulation of transforming growth factor beta receptor signaling pathway

GO molecular functions

–

GO cellular compartments

cytoplasm, nucleus

Note:
Linked to glioma / brain cancer.

Gemin5

Gem–associated protein 5

(Q8TEQ6)

(Homo sapiens)

PROTEIN

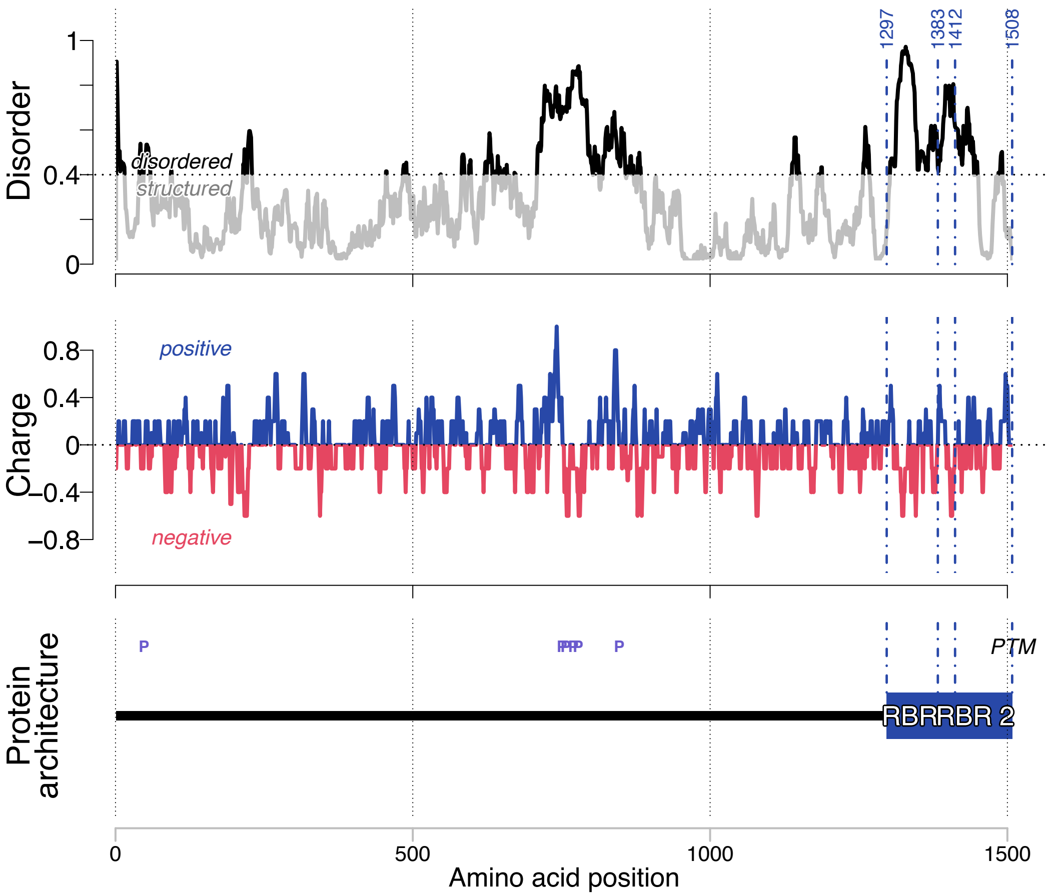

RNA BINDING DISORDERED PEPTIDE

RBR 1

1297PNSSVWVRAGHRTLSVEPSQQLDTASTEETDPETSQPEPNRPSELDLRLTEEGERMLSTF
KELFSEKHASLQNSQRTVAEVQETLAEMIRQHQKSQLCKSTANGPDKNEPEVEAEQ1412

RBR 2

1383EMIRQHQKSQLCKSTANGPDKNEPEVEAEQPLCSSQSQCKEEKNEPLSLPELTKRLTEAN
QRMAKFPESIKAWPFPDVLECCLVLLLIRSHFPGCLAQEMQQQAQELLQKYGNTKTYRRH
CQTFCM1508

GO biological processes

gene expression, mRNA splicing, via spliceosome, ncRNA metabolic process, protein complex assembly, spliceosomal snRNP assembly

GO molecular functions

poly(A) RNA binding, snRNA binding

GO cellular compartments

cytoplasm, cytosol, Gemini of coiled bodies, membrane, nuclear body, nucleoplasm, nucleus, SMN complex, SMN−Sm protein complex

Nup153

Nuclear pore complex protein Nup153

153 kDa nucleoporin; Nucleoporin Nup153
(P49790)

(Homo sapiens)

PROTEIN

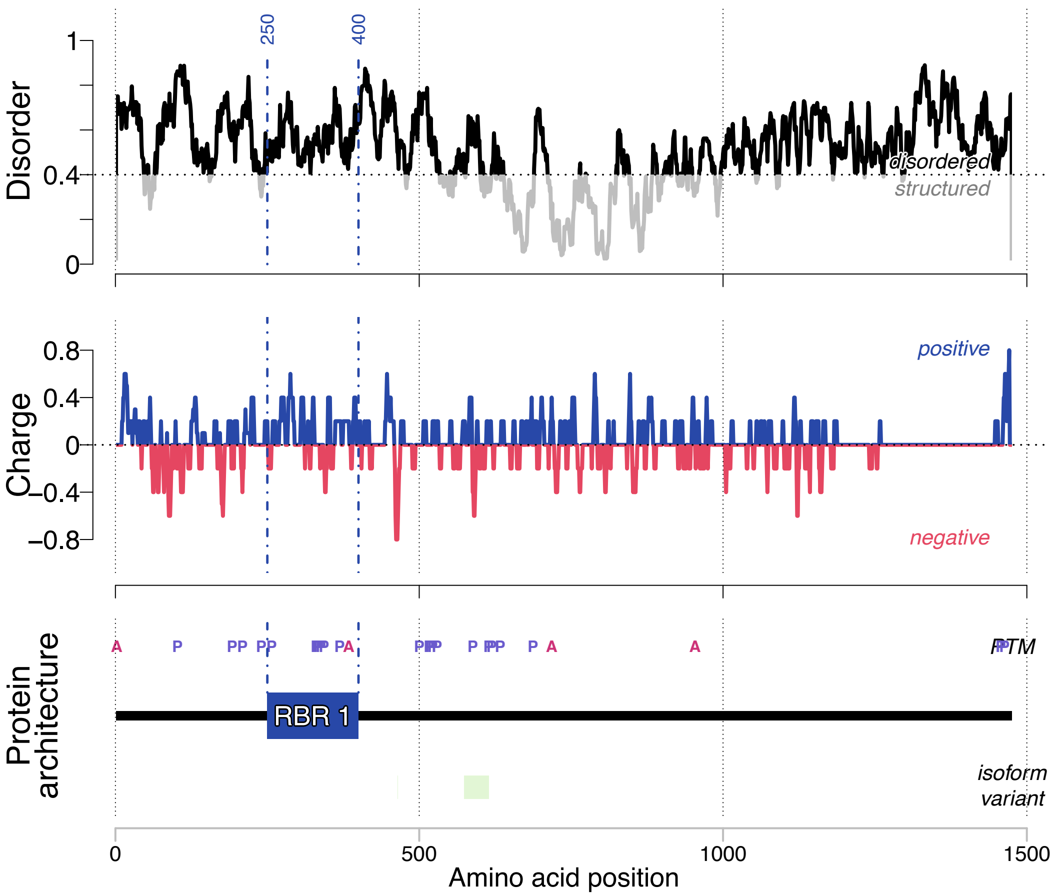

RNA BINDING DISORDERED PEPTIDE

RBR 1

250 KTSQLGDSPFYPGKTTYGGAAAAVRQSKLRNTPYQAPVRRQMKAKQLSAQSYGVTSSTAR
RILQSLEKMSSPLADAKRIPSIVSSPLNSPLDRSGIDITDFQAKREKVDSQYPPVQRLMT
PKPVSIATNRSVYFKPSLTPSGEFRKTNQRI 400

GO biological processes

carbohydrate metabolic process, cellular protein metabolic process, cellular response to h
eat, cytokine−mediated signaling pathway, gene expression, glucose transport, hexose trans
port, mitotic cell cycle, mitotic nuclear envelope disassembly, mRNA transport, negative r
egulation of RNA export from nucleus, nuclear pore complex assembly, post−translational pr...

GO molecular functions

DNA binding, identical protein binding, nucleocytoplasmic transporter activity, protein an
chor, structural constituent of nuclear pore, transporter activity, zinc ion binding

GO cellular compartments

cytoplasm, nuclear inclusion body, nuclear lamina, nuclear membrane, nuclear periphery, nu
clear pore, nuclear pore nuclear basket, nucleolus, nucleoplasm

SCML2

Sex comb on midleg–like protein 2

(Q9UQR0)

(Homo sapiens)

PROTEIN

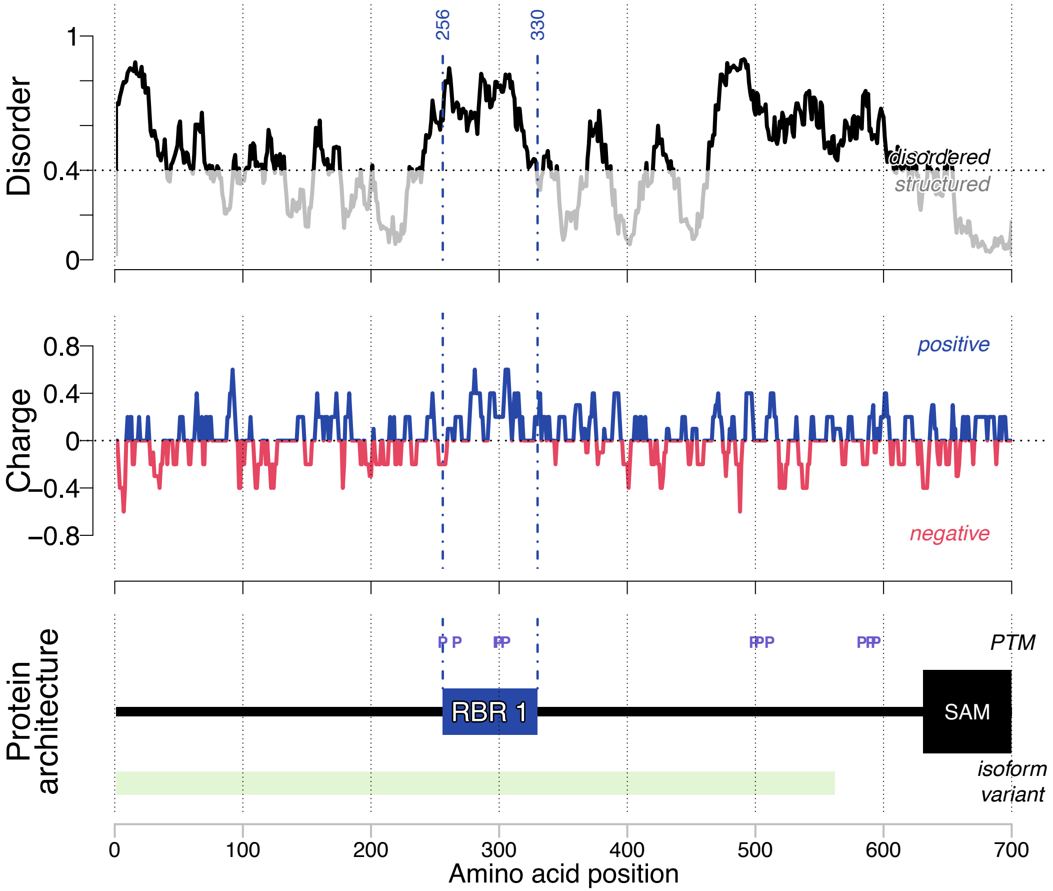

RNA BINDING DISORDERED PEPTIDE

RBR 1

256 SPSEASQHSMQSPQKTTLILPTQQVRRSSRIKPPGPTAVPKRSSSVKNITPRKKGPNSGK
KEKPLPVICSTSAAS 330

GO biological processes

anatomical structure morphogenesis, transcription, DNA−templated

GO molecular functions

DNA binding, sequence−specific DNA binding transcription factor activity

GO cellular compartments

nucleus, PcG protein complex

KDM4D

Lysine–specific demethylase 4D

JmjC domain–containing histone demethylation protein 3D; Jumonji domain–containing protein 2D

(Q6B0I6)

(Homo sapiens)

PROTEIN

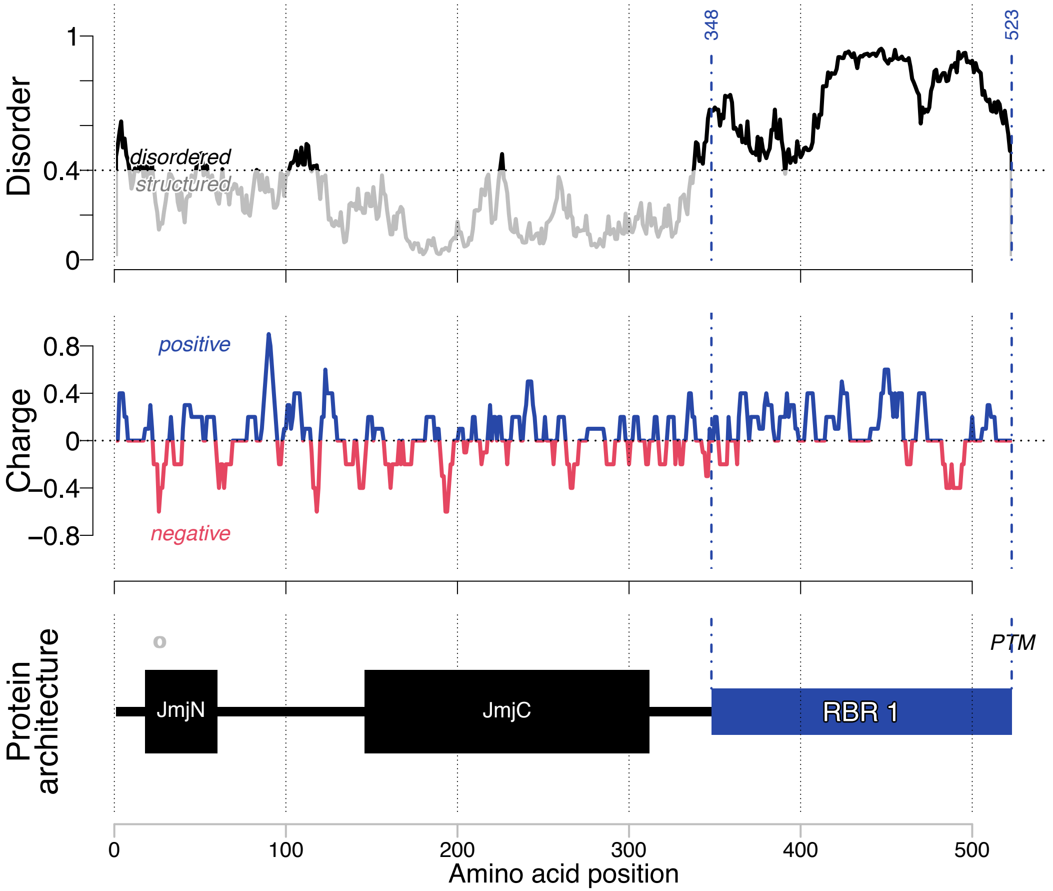

RNA BINDING DISORDERED PEPTIDE

RBR 1

348 MEPRVPASQELSTQKEVQLPRRAALGLRQLPSHWARHSPWPMAARSGTRCHTLVCSSLPR
RSAVSGTATQPRAAAVHSSKKPSSTPSSTPGPSAQIIHPSNGRRGRGRPPQKLRAQELTL
QTPAKRPLLAGTTCTASGPEPEPLPEDGALMDKPVPLSPGLQHPVKASGCSWAPVP 523

GO biological processes

chromatin organization, histone H3–K9 demethylation, regulation of transcription, DNA–temp
lated, transcription, DNA–templated

GO molecular functions

dioxygenase activity, histone demethylase activity (H3–K9 specific), metal ion binding

GO cellular compartments

blood microparticle, nucleoplasm

Note:
This region contains an RG–rich region (GRRGRGR) and di–R and di–K sequences that are good candidates residues for the reported interaction with RNA.
